# Developing quality indicators for Chronic Kidney Disease in primary care, extractable from the Electronic Medical Record. A Rand-modified Delphi method

**DOI:** 10.1186/s12882-020-01788-8

**Published:** 2020-05-05

**Authors:** Steve A. Van den Bulck, Patrik Vankrunkelsven, Geert Goderis, Gijs Van Pottelbergh, Jonathan Swerts, Karolien Panis, Rosella Hermens

**Affiliations:** 1 Academic Center for General Practice, Department of Public Health and Primary Care, KU Leuven, Kapucijnenvoer 33 blok J, 3000 Leuven, Belgium; 2 Radboud Institute for Health Sciences, Scientific Institute for Quality in Healthcare, Radboud University Medical Center, Radboud University Nijmegen, Nijmegen, Netherlands

## Abstract

**Background:**

Chronic kidney disease (CKD) is a common chronic condition and a rising public health issue with increased morbidity and mortality, even at an early stage. Primary care has a pivotal role in the early detection and in the integrated management of CKD which should be of high quality. The quality of care for CKD can be assessed using quality indicators (QIs) and if these QIs are extractable from the electronic medical record (EMR) of the general physician, the number of patients whose quality of care can be evaluated, could increase vastly. Therefore the aim of this study is to develop QIs which are evidence based, EMR extractable and which can be used as a framework to automate quality assessment.

**Methods:**

We used a Rand-modified Delphi method to develop QIs for CKD in primary care. A questionnaire was designed by extracting recommendations from international guidelines based on the SMART principle and the EMR extractability. A multidisciplinary expert panel, including patients, individually scored the recommendations for measuring high quality care on a 9-point Likert scale. The results were analyzed based on the median Likert score, prioritization and agreement. Subsequently, the recommendations were discussed in a consensus meeting for their in- or exclusion. After a final appraisal by the panel members this resulted in a core set of recommendations, which were then transformed into QIs.

**Results:**

A questionnaire composed of 99 recommendations was extracted from 10 international guidelines. The consensus meeting resulted in a core set of 36 recommendations that were translated into 36 QIs. This final set consists of QIs concerning definition & classification, screening, diagnosis, management consisting of follow up, treatment & vaccination, medication & patient safety and referral to a specialist. It were mostly the patients participating in the panel who stressed the importance of the QIs concerning medication & patient safety and a timely referral to a specialist.

**Conclusion:**

This study provides a set of 36 EMR extractable QIs for measuring the quality of primary care for CKD. These QIs can be used as a framework to automate quality assessment for CKD in primary care.

## Background

Chronic kidney disease (CKD) is a common chronic condition and a rising public health issue with increased morbidity and mortality, even at an early stage [1, 2]. CKD, defined as kidney damage or a glomerular filtration rate (GFR) <60ml/min for three months or more, has an estimated prevalence of around 11% [3, 4]. In people aged between 65 and 74 worldwide, it is estimated that one in five men and one in four women have CKD [5]. However, the concept of CKD is relatively new to patients and early disease is almost always asymptomatic [6]. The Australian Health Survey showed that only one in ten patients with evidence of kidney disease was actually aware of it [7], which illustrates how silent and under-recognized CKD is [8]. Furthermore, CKD is associated with reduced quality of life, early cardio-vascular disease and events, hospitalizations, progression to kidney failure and high healthcare cost [2, 9, 10].

The above findings emphasize the importance of identifying people with CKD at an early stage of the disease to take appropriate preventive measures as described in various evidence-based guidelines [11–13]. Primary care has a pivotal role in the early identification of CKD and the integrated management between primary and secondary CKD care, in collaboration with the patient, should be of high quality [14]. The implementation of chronic-care models have shown to improve renal and cardiovascular outcomes [15–20]. However, adherence to CKD guidelines is often low and CKD management in primary care could be improved [21–23]. The challenge for primary care is to screen the population at risk for CKD and to manage the disease appropriately [14, 24]. The electronic medical record (EMR) and more precisely, data extracted from the EMR, could be used for these purposes in an automated quality assessment [25–29]. However, in order to automate quality assessment for CKD, evidence-based and EMR-extractable quality indicators (QIs) covering all aspects of primary care for CKD are necessary.

In the past QIs for evaluating processes and outcomes of CKD care have been developed and although a systematic review identified 273 QIs, few QIs are available that focus on primary care, integrated management between primary and secondary care and EMR extractability to allow automatic audit and feedback [30–33]. Patients are also rarely involved in the design of the QIs even though a study which investigated the conservative management of kidney failure indicated that there is a discordance between QIs important for patients and for caregivers [34, 35].

The aim of our study is therefore to develop a set of EMR-extractable and evidence-based quality indicators using a multidisciplinary expert panel, including patients, which can be used as a framework to evaluate and improve the quality of primary care for patients with CKD.

## Methods

Our study was performed between November 2017 and June 2018.

### Study design

A RAND-modified Delphi method was used for the development of the QIs for CKD [35–39]. This consensus method consists of 5 steps: (1) Extraction of recommendations from international guidelines and inclusion in a written questionnaire (2) Individual assessment of the recommendations by an expert panel, including an analysis of these assessments and a feedback report (questionnaire round) (3) A consensus round with a face-to-face discussion with the panel based on the feedback report and assessment of the recommendations for their eligibility. (4) Final evaluation of the set of recommendations by the panelists (5) Transformation of the recommendations into the final set of QIs.

### Study population

The expert panel consisted of 11 members: 3 nephrologists, 3 general practitioners (GP), 1 dietician, 1 nurse and 3 patients with CKD. One of the GPs was an expert in the use of EMRs. All of the professionals worked in Belgium and were selected on the basis of their expertise with CKD. The patients had the diagnosis of CKD for at least 5 years.

### Data collection

#### Extraction of recommendations

We searched the Turning Research Into Practice (TRIP) database, which is a clinical search engine designed to allow users to quickly find high-quality research evidence [40], and MEDLINE for CKD guidelines. The search terms: “chronic kidney disease” OR “CKD” OR “chronic kidney failure” OR “renal insufficiency” AND “guideline” OR “quality indicator” OR “quality measure” OR “quality of health care” OR “quality of care” OR “recommendations” were used. The search was also extended to the World Wide Web. Both Dutch and English language guidelines were included. The quality of the guidelines was assessed using the AGREE II criteria [41]. A list of high-quality guidelines was obtained and from this list only guidelines or their updates published after 2008 were selected. Primary care guidelines were preferred since the goal of this study is the creation of quality indicators for primary care.

A list of all recommendations applicable to CKD was made. To select recommendations for inclusion in a written questionnaire, the SMART principle was used independently by two researchers (JS and KP). Disagreement was solved by discussion and if no consensus could be reached, a third researcher was consulted (SVdB). The SMART principle is known to guide the development of goals where each objective should be Specific, Measurable, Acceptable, Realistic and Time-bound [39, 42, 43]. For example, recommendations that were not specific or acceptable for primary care, were excluded because no useful indicators could be derived (e.g. ‘Patients with an estimated GFR < 20 mL/min/m2 may require initiation of renal replacement therapy if there are symptoms of uremia’). Since our goal was to develop EMR-extractable QIs, the SMART principle was also used to assess EMR-extractability.

Furthermore, if the questionnaire would remain too long for assessment by the expert panel in approximately 30-40 minutes, recommendations were selected by taking into account geography (country of origin closest to Belgium), demography (western European population or ancestry) and year of publication (most recent). The recommendations were also categorized according to subject/domain such as definition, classification, screening, diagnosis, management, treatment, medication & patient safety and referral to a specialist.

Because our expert panel included patients with CKD, a second questionnaire was prepared without the recommendations specifically aimed towards professionals. This resulted in a substantially shorter questionnaire for patients.

#### Questionnaire round

##### Completion

The panel members received the questionnaire by mail and they were asked to evaluate each recommendation with regards to its measurements of the quality of CKD care on a Likert scale ranging from 1 to 9, with 1 being the lowest score (poor recommendation to measure quality of care) and 9 being the highest score (excellent recommendation to measure the quality of care). For each recommendation, panel members were asked to confirm its relevance and its EMR-extractability. If a panel member could not assess the recommendation, there was an option to denote the recommendation as "not assessable". After each (sub) category, panel members were asked to assess the relevance of the recommendations in a top 5 (prioritization), which is explained in further detail below. The patients received assistance from one of the authors (KP or JS) to complete the questionnaire by going through each question and providing clarification when necessary.

Finally, all panel members were able to comment or suggest other recommendations.

##### Analysis

Before the consensus meeting the recommendations were divided into 3 categories: having a high, a low or an uncertain potential to measure the quality of primary care for CKD. Recommendations were allocated to these categories based on the median Likert scale scores, prioritization and the extent of consensus among the expert panel members [39].

##### Median Likert scale scores

The median of all panelists' scores for each recommendation, ranging from 1 to 9.

##### Prioritization

Prioritization was a percentage based on the score of the recommendation in a top-5. The first ranked recommendation received 5 points, the second 4 points, etc. If a recommendations was not included in the top-5 listing it received 0 points. Individual prioritization points were then added up and divided by the maximal possible points of the recommendation. For example, if 6 panelists ranked a recommendation first and 5 did not mention it in their top-5 score, the prioritization was 30/55 (11 x 5 = 55) or 54.5%.

##### Consensus

Consensus was determined as ≥70% of the panelists assigning a median Likert score of ≥7. Discussion was determined as ≥ 30% of the panelists assigning ≥7 AND ≥ 30% assigning ≤3. The other scores were determined as absence of consensus. (Table 1)

**Table 1:** Preselection and consensus criteria

Preselection	Median ≥ 7 and top percentage ≥ 20%	Selection
	Median ≥ 7 and top percentage between 1 – 20%	Discussion
	Median < 7 and top percentage ≥ 20%	Discussion
	Other	No selection
Consensus	≥ 70% in highest tertile	Consensus
	≥ 30% in highest tertile and ≥ 30% in lowest tertile	Discussion
	Other	No consensus

##### Classification of recommendations

The median Likert score and the prioritization determined the preselection of the recommendations. Based on the preselection and the consensus, the recommendations were categorized as having a high, low or uncertain potential to measure the quality of primary CKD care. (Table 1 and 2)

**Table 2:** Final classification of the recommendations into 3 categories

	Preselection	Consensus	Conclusion
Recommendation 1	Selection	Consensus	Selection
Recommendation 2	Selection	Discussion	Discussion
Recommendation 3	Selection	No consensus	Discussion
Recommendation 4	Discussion	Consensus	Discussion
Recommendation 5	Discussion	Discussion	Discussion
Recommendation 6	No selection	No consensus	No selection

#### Consensus round

A consensus round was organized to discuss the in- or exclusion of the recommendations based on the data in the feedback report. Each recommendation was awarded a color, rendering its potential for quality of care measurement: recommendations with a high, low or uncertain potential were awarded a green, red and orange color, respectively. Furthermore, the scores on the 9-point Likert scale awarded by all participants, the percentage of prioritization and the extent of consensus, were noted in the feedback report for each individual recommendation. The recommendations and comments that were added by the panelists finished the report. The high potential recommendations (green color) were briefly reviewed for inclusion, those with a low potential (red color) for exclusion. Uncertain recommendations (orange color) were discussed more extensively for in- or exclusion. Finally, the selected recommendations were discussed using the SMART principle to evaluate their EMR extractability and usefulness in primary care.

#### Final evaluation

The list of all selected recommendations was mailed to the panelists for their final evaluation.

#### Formulation of the final set QIs

The final set of recommendations was transformed into QIs. For example, the recommendation: “If a patient has CKD and the eGFR is ≥ 60 ml/min/1.73 m2 (stadium 1 and 2), test eGFR annually” was transformed into “the percentage of patients with CKD and a GFR ≥ 60 ml/min/1.73 m2 in whom the eGFR was tested annually”. The final set of QIs was approved by all authors.

### Ethical approval

This study was evaluated by the ethical committee of KU Leuven (SCONE) with number MP003409, which decided ethical approval was unnecessary. All panel members gave their written informed consent to participate in this study.

## Results

### Extraction of recommendations

**Fig. 1:**
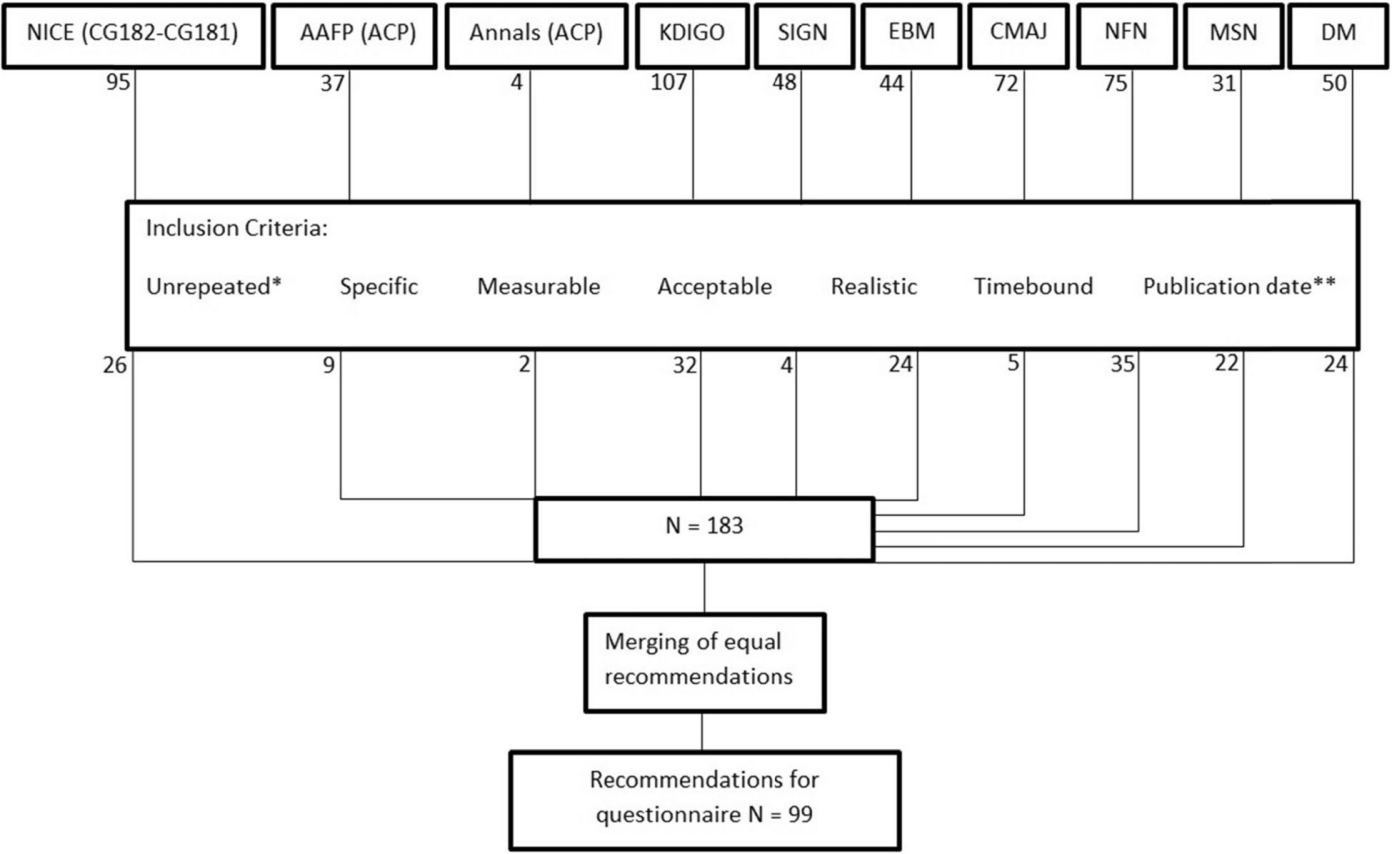
overview of recommendation extraction. *Unrepeated: repetitive recommendations were removed; ** Publication date: primary sources after 2008 were included; NICE: National Institute for health and Care Excellence; AAFP: American Association of Family Physicians; ACP: American College of Physicians; KDIGO: Kidney Disease Improving Global Outcomes; SIGN: Scottish Intercollegiate Guidelines Network; EBM: Evidence Based Medicine Practice Net; CMAJ: Canadian Medical Association Journal; NFN: Nederlandse Federatie van Nefrologen; MSN: Malaysian Society of Nephrology; DM: Domus Medica

Ten guidelines met the eligibility criteria and were screened with the AGREEII method [41]. [11, 12, 44–57] These ten guidelines were selected, based on their relevance, publication date and applicability to primary care. A total of 563 recommendations were obtained out of these guidelines. Thereafter, recommendations were classified into eight main categories (definition, classification, screening, diagnosis, etiology, management, treatment and referral to a specialist). During this process, identical recommendations and recommendations that involved specialized laboratory findings were removed. This resulted in a combined list of 390 recommendations (see Addendum 1). These 390 recommendations were evaluated and this (see Figure 1) resulted in a list of 183 recommendations, which were reduced to 99 by merging resembling recommendations. Discrepancies were solved by consensus.

### Questionnaire round

These 99 recommendations were included in the questionnaire and submitted for individual rating to the professionals. For patients with CKD, 47 recommendations were presented. These did not include the recommendations that were specifically aimed at professionals (see Addendum 2 and 3 for the questionnaires for professionals and patients, respectively).

The response rate of the questionnaire was 100%. Based on preselection and consensus results of both the professional and the patients’ questionnaire, 26 out of 99 recommendations were selected, 43 needed further discussion and 30 did not meet the selection criteria.

### Consensus round

A consensus meeting was organized with 7 panel members participating (1 general practitioner, 2 nephrologists, 1 dietician, 1 nurse and 2 patients). In this round, 24 out of 26 pre-panel selected recommendations remained selected; 23 out of 43 pre-panel recommendations, categorized as “discussion”, were also selected and 6 out of 30 non-selected pre-panel recommendations were selected (see Addendum 4). These 53 recommendations were then further discussed during the consensus meeting by applying the SMART principle to evaluate EMR-extractability or to improve measurability. Some recommendations were rejected, some needed minimal modification or were merged, which resulted in a final set of 36 recommendations.

### Final evaluation

After the consensus round, 36 recommendations were mailed to the panelists for final approval. All panelists approved the final set of recommendations in 2 rounds. After the first round, 6 recommendations were slightly adapted based on the response of the panelists who were unable to participate in the consensus meeting.

### Formulation of the final set QIs

The 36 recommendations were all transformed into QIs, resulting in 1 QI concerning definition/classification, 1 QI concerning screening/diagnosis, 8 QIs concerning management, 8 QIs concerning treatment, 6 QIs concerning medication and patient safety and 12 QIs concerning referral to a specialist. (see Table 3).

**Table 3:** Quality indicators for CKD

Nr	Definition and classification
1a	Percentage of patients with a GFR <60 ml/min/1.73 m^2^ for 3 months, diagnosed with CKD (= diagnosis of CKD present in the problem list of the EMR)
1b	Percentage of patients with a GFR < 60 ml/min/1.73 m^2^, in whom no other measurement was found, who were tested within 3 months
	Screening and diagnosis
2	Percentage of patients with hypertension, diabetes mellitus, chronic NSAID use or a history of heart- and blood vessel disease, in whom eGFR and albuminuria is determined once a year
	Management: Follow-up
3	Percentage of patients with CKD with a GFR ≥ 60 ml/min/1.73 m^2^ , whose eGFR is tested annually
4	Percentage of patients with CKD with a GFR between 30-59 ml/min/1.73 m^2^, who are tested every six months
5	Percentage of patients with CKD with a GFR between 15-29 ml/min/1.73 m^2^,who are tested every three months
6	Percentage of patients with CKD, in whom the GFR, albuminuria and total protein is determined at least once a year
7a	Percentage of patients with CKD with: - macroalbuminuria or - microalbuminuria in combination with an eGFR <60 or - eGFR < 45 ml/min/1.73 m^2^ in whom serum potassium (on a fresh blood sample), calcium, phosphate, PTH and bicarbonate levels, and Hb is measured at least once a year.
7b	Percentage of patients with CKD with macroalbuminuria or microalbuminuria and eGFR <60 or eGFR < 45 ml/min/1.73 m^2^ and an increased PTH, in whom vitamin D is measured at least once a year.
7c	Percentage of patients with CKD with macroalbuminuria or microalbuminuria and eGFR <60 or eGFR < 45 ml/min/1.73 m^2^ and a reduced Hb, in whom ferritin and transferrin saturation is measured at least once a year.
	Management: Vaccination
8	Percentage of patients with CKD who are vaccinated with an influenza vaccine annually
9	Percentage of patients with CKD who are vaccinated with a pneumococcal vaccine
10	Percentage of patients with CKD with progressive disease (= increasing proteinuria and/or decreasing eGFR) who are vaccinated with a hepatitis B vaccine
	Treatment CKD
11	Percentage of patients with CKD , who are encouraged to undertake physical activity compatible with cardiovascular health and tolerance (aiming for at least 30 minutes 5 times per week), to stop smoking and obtain or maintain a healthy weight (BMI 20 to 25; waist circumference ≤ 94 cm in men or ≤ 80 cm in women) and to limit alcohol intake
12	Percentage of patients with CKD with a normal albuminuria (<30mg/g or <30 mg/24hours) and an office blood pressure consistently >140 mm Hg systolic or >90 mm Hg diastolic, who are treated with blood pressure-lowering drugs to maintain a blood pressure that is consistently ≤140 mm Hg systolic and ≤90 mm Hg diastolic
13	Percentage of patients with CKD with a strongly increased albuminuria (> 300 mg/g or > 300 mg/24 hours) or moderately increased albuminuria (30-300 mg /g or 30-300 mg / 24 hours), who are treated with an ARB or an ACE inhibitor
14	Percentage of patients with CKD with an albuminuria ≥300 mg/g creatinine (or ≥300 mg/24 hours or proteinuria ≥0.5 g/24 hours), who are treated by increasing ACE inhibitor or ARB dosage if the maximum dose has not yet been reached.
15	Percentage of patients with CKD in whom a one-time lipid profile (total cholesterol, LDL cholesterol, HDL cholesterol, triglycerides) is determined
16	Percentage of patients with CKD with - proteinuria ≥1 g/day or - diabetes mellitus or - strongly increased albuminuria (ACR >300 mg/g creatinine or >300 mg/24 hours) or - ACR ≥700 mg/g in whom the target BP of < 130/80 mmHg (SBP range 120 - 129 mmHg) is achieved by using the first choice antihypertensive drugs (ACE inhibitors or ARBs)
17	Percentage of patients with CKD with a reduced serum bicarbonate (<20 mmol / L), who are treated with oral bicarbonate to keep the serum bicarbonate level within the normal range
18	Percentage of patients with CKD with a vitamin D deficiency (<15 ng/ml), who are substituted with vitamin D
	Medication and safety of the patient
19	Percentage of patients with CKD with a RAAS blocker or spironolactone treatment, in whom potassium level and GFR are measured before initiating this therapy and controlled within 1 week after initiation and after each dose increase
20	Percentage of patients with CKD who need an examination with contrast medium and of whom no recent value (last 12 months) of eGFR is known, in whom eGFR is determined
21	Percentage of patients with CKD with a GFR < 30 ml/min./1.73m^2^, in whom metformin is avoided/ is not prescribed
22	Percentage of patients with CKD with a GFR < 30 ml/min/1.73 m^2^, in whom bisphosphonate treatment is avoided/ is not prescribed
23	Percentage of patients with CKD with a GFR < 60 ml/min/1.73 m^2^, in whom oral phosphate-containing bowel preparations are avoided/ are not prescribed
24	Percentage of patients with CKD who are referred to a specialist, and for whom the referral letter includes active diagnosis of CKD and renal function
	Referral to a specialist
25	Percentage of patients with a GFR <30 ml/min/1,73 m^2^ , who are referred to a specialist
26	Percentage of patients with CKD with hypertension who do not respond to medical treatment with 4 or more antihypertensive drugs, who are referred to a specialist
27	Percentage of patients with CKD who are pregnant or are planning pregnancy, who were referred to a specialist
28	Percentage of patients with CKD with: - a chronic eGFR <30 ml/min./1,73 m^2^ - an eGFR between 30-45 ml/min./1,73 m^2^ and ACR >200 mg/g for men or 300 mg/g for women , and/or proteinuria >1000 mg/24h or a protein-creatininratio (PCR) > 1 000 mg/g who are referred to a specialist for inclusion in a care program
29	Percentage of patients with a confirmed significant albuminuria (ACR ≥300 mg/g or AER ≥ 300 mg/ 24 hours), who are referred to a specialist
30	Percentage of patients with hematuria in combination with proteinuria (urine protein ≥0.5 g/day or PCR ≥500mg/g or ACR ≥ 300 mg/g creatinin), who are referred to a specialist
31	Percentage of patients with progression of CKD (confirmed decline in GFR category accompanied by a 25% or greater drop in eGFR from baseline or a sustained decline in eGFR of more than 5 ml/min/1.73 m^2^ /year), who are referred to a specialist
32	Percentage of patients with persistent microscopic hematuria of unknown origin, who are referred to a specialist
33	Percentage of patients with persistent serum potassium abnormalities (potassium <4.0 mmol / l or potassium >5.5 mmol / l ), who are referred to a specialist
34	Percentage of patients with recurrent or extensive nephrolithiasis, who are referred to a specialist
35	Percentage of patients with hereditary kidney disease, who are referred to a specialist
36	Percentage of patients with an arteria renalis stenosis, who are referred to a specialist

CKD Chronic Kidney Disease, *GFR* Glomerular Filtration Rate, *PTH* Parathyroid hormone, *Hb* Hemoglobin, *ARB* Angiotensin II receptor blockers, *ACEi* angiotensin converting enzyme inhibitor, *RAAS* renin–angiotensin–aldosterone system, *PCR* Protein-Creatinine Ratio, *ACR* albumin to creatinine ratio, *AER* albumin excretion rate, *NSAID* non-steroidal anti-inflammatory drug

## Discussion

### Principal findings

We used a RAND-modified Delphi method to develop a set of 36 quality indicators which are EMR- extractable and which can be used to evaluate primary care for patients with CKD. This set of QIs is based on 10 (inter) national guidelines and includes a wide range of aspects of care for patients with CKD, namely definition & classification, screening, management: follow-up & vaccination, treatment of CKD, medication & safety of the patient and referral to a specialist. These EMR- extractable QIs can be used as a framework to improve the quality of primary care for patients with CKD.

The first QI with regard to definition & classifications is important because a correct diagnosis in the problem list of the EMR will be the basis for the utilization of many other QIs. After all, if the diagnosis is not correctly recorded in the EMR, a quality measurement evaluating the care of CKD will produce unreliable results, since patients who do not have a correct diagnosis in their problem list will not be recognized. Applying this QI in an EMR system is thus important and can be a part of the implementation of the so-called electronic CKD phenotype, as described by other authors [13, 58]. Furthermore, the quality indicators we developed are mainly process indicators, rather than outcome indicators. This is because we based our QIs on recommendations extracted from guidelines. For the purpose of quality assessment, process indicators are also more useful than outcome indicators [59]. Outcome indicators require long-term follow-up and are influenced by many other factors that are beyond the control of professionals (i.e. patient’s age, lifestyle choices, risk factors, compliance, and health status) [60]. In addition, the QIs we developed are EMR-extractable, which makes it possible to develop an automated quality assessment. This in turn could lead to an increase in the number of patients with CKD whose quality of care can be evaluated.

The inclusion of 12 QIs concerning referral to a specialist is not surprising, given the importance of integrated care pathways [15]. At the consensus meeting, the panel’s nephrologists expressed an opinion regarding the minimal acceptable level of information and investigations required for a referral to secondary care. Despite diabetes mellitus being an important cause of CKD, many recommendations about diabetes mellitus did not make it to the final set of quality indicators. The panel members agreed that most of them were more specific for the quality of care for patients with diabetes mellitus. NSAID use was only part of the QI about screening and not a QI on its own although it was the topic of 7 recommendations in the primary list we based our questionnaire on. The recommendations concerning the use of NSAIDs were not applicable to the SMART principle and were therefore not included in the questionnaire. However, during the consensus meeting the panel stressed the importance of mentioning NSAID use in our final list of recommendations. We do not provide any QI for the target value of LDL-cholesterol, which was the topic of 5 recommendations in the primary list, but which was not included in the questionnaire. These recommendations did not reach the final QI list because they were either not considered applicable to the SMART principle, or were not included at the consensus meeting. Nutritional guidance was not very prominent among the primary list of recommendations we based our questionnaire on. This can explain why previous work indicated a lack of QIs concerning lifestyle management [32]. Our final list of QIs included 1 indicator concerning lifestyle management. However, different aspects of lifestyle management are part of this QI, namely physical activity, smoking cessation, weight control and limiting alcohol intake.

QIs regarding the care of patients with CKD have been identified in previous work [30–32, 61, 62]. The differences we noticed between our work and that of other authors are mainly EMR- extractability of our list of QIs and the focus on referral to secondary care, emphasizing the importance of a timely referral to a specialist in the case of progressive CKD. We also included patients in the development of this set of QIs to avoid the discordant priorities that might exist in QIs between those receiving the care and those providing the care for CKD [34]. Patients were in agreement with clinical staff regarding the importance of a timely referral to a specialist, which also explains the higher number of QIs on this topic. Finally, the patients also stressed the importance of the QIs on medication and patient safety.

In addition, this study provides a set of QIs that cover all aspects of primary care for CKD. To this end, we used many different (inter) national guidelines to be as complete as possible. Use of the SMART principle for the development of QIs as in earlier work, has enabled us to incorporate our goals in the development of this set of QIs, namely EMR-extractability and the possibility to use this set as a framework to evaluate the primary care for CKD [39, 43]. We thus created the opportunity to automate quality assessment for CKD in primary care, which may help improve the care for CKD [26].

### Strengths and limitations

One of the strengths of our study is that we developed our QIs based on 10 (inter) national guidelines, which provides our set of QIs with an extensive basis and a high validity. These QIs may therefore be used to evaluate care in other countries [35, 37, 39]. Furthermore, our panel included health professionals from the different disciplines involved in CKD care. Besides general physicians, nephrologists, a nurse and a dietician, 3 patients were also engaged in the design of this set of QIs, which has been lacking in many studies to date [31, 61]. After all, to provide good quality of care, it is important that the needs and preferences of the patients are taken into account [63]. Applying the SMART principle to develop this set of QIs made it possible to incorporate our main goals, namely extractability out of the EMR of the general physician and the creation of a framework to evaluate primary care for CKD. Taken together, this may pave the way for automated quality assessment.

Our study also has several limitations. We have not yet conducted practice testing of this set of QIs to confirm operational validity. Using a practice test prior to usage of QIs is an integral part of the implementation strategy and an essential step of the quality loop [35]. In a future study, these tests should be conducted in Belgium, using a clinical practice test. However, major difficulties are not expected because of the EMR-extractability of this set of QIs. We also did not test the EMR- extractability of these QI’s in practices outside Belgium since it is challenging to overlook international disparities between EMR systems. Thus, if these QIs are used outside of Belgium, EMR-extractability needs to be confirmed and a practice test needs to be performed. In addition, since these QIs are based on different recommendations with varying levels of evidence, it is not clear that an external group of experts would come to the same conclusions. Furthermore, there is a lack of a consistent relationship between process and outcome measures, where there is not always evidence that implementation of certain processes leads to improved outcomes. Although, in view of quality improvements, process indicators should remain central [64]. Finally, an important aspect of using data stored in the EMR is the completeness. Incomplete data could hamper the implementation of these EMR- extractable QIs. Potential sources of bias when using EMR data also need to be considered [65].

## Conclusion

This study provides a set of 36 EMR-extractable quality indicators for CKD primary care, based on international guidelines and approved by medical professionals and patients. These quality indicators can be used as a framework to measure and improve the quality of primary care for CKD.

## Supplementary information

**Additional file 1.** Addendum 1: Exhaustive list of recommendations

**Additional file 2.** Addendum 2: Questionnaire for the professionals

**Additional file 3.** Addendum 3: Questionnaire for the patients

**Additional file 4.** Addendum 4: Table of recommendations switched from category

## Data Availability

The materials are available as additional files and the datasets (individual feedback reports) are available from the corresponding author on request.

## Abbreviations

CKD: Chronic Kidney Disease
EMR: Electronic Medical Record
QIs: Quality Indicators
