# Supplementary material for: Developing quality indicators for Chronic Kidney Disease in primary care, extractable from the Electronic Medical Record. A Rand-modified Delphi method
Source: BMC Nephrol. 2020 May 5;21:161. doi: 10.1186/s12882-020-01788-8 (PMC7201612; doi:10.1186/s12882-020-01788-8)
Supplement: Supplementary file 1 — Additional file 1. Addendum 1: Exhaustive list of recommendations [file 12882_2020_1788_MOESM1_ESM.docx]

**Addendum 1: Exhaustive list of recommendations**

**Table of Contents**

1. Definition and classification

2. Sampling

3. CKD and Outcome

4. CKD Screening, diagnosis and etiology

5. CKD Management: follow-up

6. CKD Management: Vaccination, risk of hospitalization and mortality

7. CKD Treatment: General

8. CKD Treatment: Patient education and information

9. CKD Treatment: Lifestyle and diet

10. CKD Treatment: Uricemia

11. CKD Treatment: Diabetes and glycemic control

12. CKD Treatment: Dyslipidemia

13. CKD Treatment: Hypertension

14. CKD Treatment: Cardiovascular disease

15. CKD Treatment: Anemia

16. CKD Treatment mineral metabolism abnormalities

17. Alternations in medication and safety of the patient

18. Safety: Imaging and contrast agents

19. Referral to specialist

20. Renal replacement therapy

21. Role of the GP and care program

| 1. **Definition and classification** | | | | |
| --- | --- | --- | --- | --- |
|  | **Recommended indicator** | **Source** | **Date** | **Evidence grading** |
| 1.1 | CKD is defined as abnormalities of kidney structure or function, present for >3 months, with implications for health. (Not Graded) | KDIGO  NfN | 2012  2015 | None  None |
| 1.2 | In people with GFR < 60 ml/min/1.73 m^2^ (GFR categories G3a-G5) or markers of kidney damage, review past history and previous measurements to determine duration of kidney disease. (Not Graded)   - If duration is >3 months, CKD is confirmed. Follow recommendations for CKD. - If duration is not >3 months or unclear, CKD is not confirmed. Patients may have CKD or acute kidney diseases (including AKI) or both and tests should be repeated accordingly. | KDIGO  AAFP  DM | 2012  2004  2012 | None  C  Consensus |
| 1.3 | We recommend that CKD is classified based on cause, GFR category, and albuminuria category (CGA). (1B) | KDIGO  NfN | 2012  2015 | 1B  1B |
| 1.4 | Classification of chronic kidney disease (CKD) should be based on the existing NKF-KDOQI* staging (refer to Table 3). (Grade C) (the KDOQI exists out of 6 GFR categories and 3 albuminuria categories) | MSN  SIGN 103  NfN | 2011  2008  2015 | C  GPP None |
| 1.5 | The suffix (p) should be added to denote the presence of proteinuria when staging CKD. (Grade C) | MSN | 2011 | C |
| 1.6 | Assign cause of CKD based on presence or absence of systemic disease and the location within the kidney of observed or presumed pathologic-anatomic findings. (Not Graded) | KDIGO | 2012 | None |
| 1.7 | Use the person's GFR and ACR categories (see table 1) to indicate their **risk of adverse outcomes** (for example, CKD progression, acute kidney injury, all-cause mortality and cardiovascular events) and discuss this with them | NICE CG182 | 2014 (2015 update) | None |

| 1. **Sampling** | | | | |
| --- | --- | --- | --- | --- |
| **Recommended indicator** | | **Source** | **Date** | **Evidence grading** |
| 2.1 | Advise people not to eat any meat in the 12 hours before having a blood test for eGFR creatinine. Avoid delaying the dispatch of blood samples to ensure that they are received and processed by the laboratory within 12 hours of vena puncture | NICE CG182 | 2014 (2015 update) | None |
| 2.2 | Depending on the creatinine method used, staging of chronic kidney disease should not be based on blood samples which have been separated 16 hours or more after collection. | SIGN 103 | 2008 | GPP |
| 2.3 | Staging of chronic kidney disease (see section 2.5.1) should not be based on samples collected after consumption of meals containing cooked meat. Confirmatory samples should be taken in the fasting state. | SIGN 103 | 2008 | GPP |

| 1. **CKD and Outcome** | | | | | |
| --- | --- | --- | --- | --- | --- |
|  | **Recommended indicator** | **Source** | **Date** | **Evidence grading** | |
| 3.1 | For patients with CKD with a mild to intermediate risk: Although symptoms usually present in patients with CKD who have kidney damage with a strongly increased risk (code red), patients also have an increased risk of developing cardiovascular disease and progressive loss of renal function with kidney damage in case of a mild (code yellow) to moderate (code orange) risk. (Not graded) | NfN | 2015 | | None |
| 3.2 | The life expectancy of people with CKD decreases with increasing severity of kidney damage (not graded). | NfN | 2015 | | None |
| 3.3 | In patients with CKD with a high risk (code red) symptoms and complications of multiple organ systems occur (not graded). | NfN | 2015 | | None |
| 3.4 | We recommend that all people with CKD be considered at increased risk for cardiovascular disease. | KDIGO | 2012 | | 1A |
| 3.5 | Do not use a risk assessment tool to assess CVD risk in people with an estimated glomerular filtration rate (eGFR) less than 60 ml/min/1.73 m2 and/or albuminuria[[4](https://www.nice.org.uk/guidance/cg181/chapter/1-Recommendations#ftn.footnote_4)]. These people are at increased risk of CVD. | NICE CG181 | 2014 (2016 update) | | None |
| 3.6 | We recommend that all people with CKD are considered to be at increased risk of AKI. | KDIGO | 2012 | | 1A |

| 1. **CKD Screening, diagnosis and etiology** | | | | |
| --- | --- | --- | --- | --- |
|  | **Recommended indicator** | **Source** | **Date** | **Evidence grading** |
| 4.1 | ACP recommends against screening for chronic kidney disease in asymptomatic adults without risk factors for chronic kidney disease. | ACP/AAFP  Annals  AAFP | 2013  2013  2014 | (Grade: weak recommendation, low-quality evidence) |
| 4.2 | ACP recommends against testing for proteinuria in adults with or without diabetes who are currently taking an angiotensin-converting enzyme inhibitor or an angiotensin II–receptor blocker. | ACP/AAFP  Annals  AAFP | 2013  2013  2014 | (Grade: weak recommendation, low-quality evidence) |
| 4.3 | A ratio of greater than 30 mg of albumin to 1 g of creatinine on untimed (spot) urine testing is abnormal and merits further evaluation. | AAFP | 2004(I) | C |
| 4.4 | Regard a confirmed ACR of 3 mg/mmol or more as clinically important proteinuria | NICE CG182 | 2014 (2015 update) | None |
| 4.5 | In most circumstances, untimed (spot) urine samples, rather than 24-hour urine collections, should be used to detect and monitor proteinuria. | AAFP | 2004 (II) | C |
| 4.6 | Instead of a timed urine collection, a random urine sample for the microalbumin-creatinine or protein-creatinine ratio should be used to quantify proteinuria. | AAFP | 2005 | C |
| 4.7 | Confirm a positive test strip (1+ or more) with a quantitative measurement and express it as a ratio to creatinine (ACR or PCR). | NfN  KDIGO  AAFP  MSN | 2015  2012  2004 (II)  2011 | None  None  C  C |
| 4.8 | Urine dipstick testing cannot be used reliably in isolation to diagnose the presence or absence of proteinuria | SIGN 103 | 2008 | GPP |
| 4.9 | Urine dipsticks should be used to screen for proteinuria. (Grade C) | MSN | 2011 | C |
| 4.10 | Dipstick proteinuria (≥1+) can be used to identify patients at risk of subsequent end stage renal disease and cardiovascular disease. | SIGN 103 | 2008 | GPP |
| 4.11 | In patient groups with a high prevalence of proteinuria without diabetes, protein/creatinine ratio may be used to exclude chronic kidney disease | SIGN 103 | 2008 | B |
| 4.12 | Do not use reagent strips to identify proteinuria unless they are capable of specifically measuring albumin at low concentrations and expressing the result as an ACR.) (GRADE 1B). | EBM  NICE CG182 | 2017 2014 (2015 update) | 1B None |
| 4.13 | Screening for proteinuria should be performed for all patients who are at high risk of kidney disease (patients with diabetes, hypertension, vascular disease, autoimmune disease, estimated glomerular filtration rate < 60 mL/min/1.73m2 or edema) (grade D, opinion). | CMAJ | 2008 | D |
| 4.14 | Screening should be performed by random urine samples to measure the ratio of protein to creatinine or of albumin to creatinine. For patients with diabetes, testing of the ratio of albumin to creatinine should be performed to screen for kidney disease (grade B). | CMAJ | 2008 | B |
| 4.15 | A ratio of protein to creatinine > 100 mg/mmol or a ratio of albumin to creatinine > 60 mg/mmol should be considered as thresholds to indicate high risk of progression to end-stage renal disease (grade D). | CMAJ | 2008 | D |
| 4.16 | Evaluation of albuminurie: We suggest using the following measurements for initial testing of proteinuria (in descending order of preference, in all cases an early morning urine sample is preferred) (2B):  1) urine albumin-to-creatinine ratio (ACR);  2) urine protein-to-creatinine ratio (PCR);  3) reagent strip urinalysis for total protein with automated reading;  4) reagent strip urinalysis for total protein with manual reading. | NfN  KDIGO | 2015  2012 | 2B  2B |
| 4.17 | To detect and identify proteinuria, use urine ACR in preference to protein:creatinine ratio (PCR), because it has greater sensitivity than PCR for low levels of proteinuria. For quantification and monitoring of levels of proteinuria of ACR 70 mg/mmol or more, PCR can be used as an alternative. ACR is the recommended method for people with diabetes | NICE CG182 | 2014 (2015 update) | None |
| 4.18 | For the initial detection of proteinuria, if the ACR is between 3 mg/mmol and 70 mg/mmol, this should be confirmed by a subsequent early morning sample. If the initial ACR is 70 mg/mmol or more, a repeat sample need not be tested .(Grade 1B) | EBM  NICE CG182  NfN  KDIGO | 2017  2014 (2015 update)  2015  2012 | 1B  None  None  None |
| 4.19 | If significant non-albumin proteinuria is suspected, use assays for specific urine proteins (e.g., a1-microglobulin,  monoclonal heavy or light chains, [known in some countries as ‘‘Bence Jones’’ proteins]). | EBM  KDIGO | 2017  2012 | 1B  None |
| 4.20 | Determine the corrected proteinuria in non-diabetics  Determine the corrected albuminuria in diabetics. | DM | 2012 | 1B Consensus |
| 4.21 | Determine the corrected albuminuria or corrected proteinuria in patiënts with an eGFR <60 ml / min / 1.73 m2 (Grade 1C). | DM | 2012 | 1C |
| 4.22 | Quantify by laboratory testing the urinary albumin or urinary protein loss of people with a GFR of 60 ml/min/1.73 m^2^ or more if there is a strong suspicion of CKD | NICE CG182 | 2014 (2015 update) | None |
| 4.23 | If a more accurate estimate of albuminuria or total proteinuria is required, measure albumin excretion rate or total protein excretion rate in a timed urine sample. | NfN  KDIGO | 2015  2012 | None  None |
| 4.24 | During treatment, aim to keep albumin concentrations <30 mg / mmol (or <300 mg / 24 hours, or proteinuria concentrations ​​<0.5 g / 24 hours), independent of blood pressure. This can be achieved by increasing the dose renin–angiotensin system antagonists or Angiotensin receptor Blockers, or combining these with dietary salt restriction of 3 to 5 gram and / or a (thiazide) diuretic. In this case, determine feasibility individually (grade 2D). | NfN | 2015 | 2D |
| 4.25 | Offer testing for CKD using eGFR creatinine and ACR to people with any of the following risk factors:   - diabetes - hypertension - acute kidney injury (see [recommendation 1.3.9](http://www.nice.org.uk/guidance/cg182/chapter/recommendations#acute-kidney-injury-and-ckd)) - cardiovascular disease (ischaemic heart disease, chronic heart failure, peripheral vascular disease or cerebral vascular disease) - structural renal tract disease, recurrent renal calculi or prostatic hypertrophy - multisystem diseases with potential kidney involvement – for example, systemic lupus erythematosus - family history of end-stage kidney disease (GFR category G5) or hereditary kidney disease - opportunistic detection of haematuria. | NICE CG182 | 2014 (2015 update) | None |
| 4.26 | Screen for renal insufficiency in patients with:   - diabetes (Grade 1C); - hypertension (Grade 1C); - ischemic heart disease and / or cardiac decompensation and / or peripheral vascular disease and / or cerebral vascular disease (Grade 1C); - a family history of renal insufficiency stage 5 or kidney disease that runs in the family (Grade 2C). | DM | 2012 | 1C 1C 1C 2C |
| 4.27 | Screening can be considered for patients with:   - Age >65 years old - Family history of stage 5 CKD or hereditary kidney disease - Structural renal tract disease, renal calculi or prostatic hypertrophy - Opportunistic (incidental) detection of haematuria or proteinuria - Chronic use of non-steroidal anti-inflammatory drugs (NSAIDs) or other nephrotoxic drugs - Cardiovascular disease (CVD) - Multisystem diseases with potential kidney involvement such as systemic lupus erythematosus. (Grade C) | MSN | 2011 | C |
| 4.28 | In patients with known renal disease (eg autosomal dominant polycystic kidney disease) or systemic disease in whom glomerular abnormalities may occur (eg SLE) and in patients with urological problems (recurrent pyelonephritis, spina bifida, reflux), in whom eGFR and albuminuria are still normal, it is desirable to examine (not graded) blood pressure, eGFR, albuminuria / proteinuria and erythrocyturia at least once a year. | NfN | 2015 | None |
| 4.29 | In patients with risk factors for developing CKD, such as hypertension or diabetes mellitus or a history of cardiovascular disease, it is desirable to measure eGFR and albuminuria once a year (not graded). | NfN  MSN  SIGN 103 | 2015  2011 2008 | None  C  GPP |
| 4.30 | Adults with cardiovascular disease should be screened for CKD. | AAFP | 2011 | C |
| 4.31 | Physicians should screen at-risk populations for CKD using serum creatinine levels and random urine testing for albuminuria | AAFP | 2011 | C |
| 4.32 | All adults with risk factors for chronic kidney disease should be screened with a serum creatinine determination for GFR estimation and analysis of a random urine sample for proteinuria. | AAFP | 2005 | C |
| 4.33 | All patients with diabetes should have regular surveillance of renal function. | SIGN 103 | 2008 | D |
| 4.34 | In patients with diabetes, albumin/creatinine ratio may be used to exclude diabetic nephropathy. | SIGN 103 | 2008 | B |
| 4.35 | Albumin/creatinine ratio is recommended for detecting and monitoring diabetic nephropathy | SIGN 103 | 2008 | C |
| 4.36 | In patients with diabetes, albumin: creatinine ratio (ACR) on an early morning spot urine sample should be performed at least annually to screen for microalbuminuria if urine dipstick is negative. (Grade C) | MSN | 2011 | C |
| 4.37 | Do not use age, gender or ethnicity as risk markers to test people for CKD. In the absence of metabolic syndrome, diabetes or hypertension, do not use obesity alone as a risk marker to test people for CKD | NICE CG182 | 2014 (2015 update) | None |
| 4.38 | Patients with persisting isolated microscopic haematuria should be initially evaluated for urinary tract infection and malignancy. | SIGN 103  MSN  NICE CG182 | 2008  2011  2014( 2015 update) | D  C  None |
| 4.39 | Persistent invisible haematuria in the absence of proteinuria should be followed up annually with repeat testing for haematuria, proteinuria or albuminuria, GFR and blood pressure monitoring as long as the haematuria persists. | NICE CG182 | 2014 (2015 update) | None |
| 4.40 | Ultrasound is the imaging modality of choice in the evaluation of patients with suspected chronic kidney disease. | SIGN 103 | 2008 | GPP |
| 4.41 | Do not diagnose CKD in people with:   - an eGFRcreatinine of 45–59 ml/min/1.73 m2 and - an eGFRcystatinC of more than 60 ml/min/1.73 m2 and - no other [marker of kidney disease](http://www.nice.org.uk/guidance/cg182/chapter/recommendations#markers-of-kidney-disease). | NICE CG182 | 2014 (2015 update) | None |
| 4.41 | Evaluate the clinical circumstances, including personal and family history, social and environmental factors, medications, physical examination, laboratory measures, imaging, and pathologic diagnosis to determine the causes of kidney disease. (Not Graded) | NfN  KDIGO | 2015  2012 | None  None |
| 4.42 | It is recommended to use the CKD-EPI formula to estimate kidney function (glomerular filtration rate) (grade 1B) | NfN | 2015 | 1B |
| 4.43 | Determine creatinine with eGFR (calculated according to the MDRD formule) in screening for CKD (Grade 1A). | DM  MSN | 2012  2011 | 1A  C |
| 4.44 | We recommend using serum creatinine and a GFR estimating equation for initial assessment. (1A) | KDIGO  MSN | 2012  2011 | 1A  C |
| 4.45 | We recommend that clinicians use a GFR estimating equation to derive GFR from serum creatinine (eGFRcreat) rather than relying on the serum creatinine concentration alone. | KDIGO  SIGN 103 | 2012  2008 | 1B  C |
| 4.46 | Clinicians need to understand the clinical settings in which eGFRcreat is less accurate. | NfN  KDIGO | 2015  2012 | 1B 1B |
| 4.47 | We suggest using additional tests (such as cystatin C or a clearance measurement) for confirmatory testing in specific circumstances when eGFR based on serum creatinine is less accurate. (2B) | KDIGO | 2012 | 2B |
| 4.48 | We suggest measuring cystatin C in adults with eGFRcreat 45–59 ml/min/1.73 m2 who do not have markers of kidney damage if confirmation of CKD is required.   - If eGFR cys /eGFRcreat-cys is also < 60 ml/min/1.73 m2, the diagnosis of CKD is confirmed. - If eGFRcys/eGFR creat-cys is >/= 60 ml/min/1.73 m2, the diagnosis of CKD is not confirmed. | KDIGO | 2012 | 2C |
| 4.49 | In specific circumstances, eg if the result is used to support a treatment option, it is suggested to estimate the GFR using another method, such as an estimation with exogenous markers (such as iothalamate or iohexol), cystatin C or 24 hour creatinin and / or urea clearance ( grade 2B). | NfN | 2015 | 2B |
| 4.50 | In predicting risk for outcome of CKD, identify the following variables:  1) cause of CKD;  2) GFR category;  3) albuminuria category;  4) other risk factors and comorbid conditions. (Not Graded) | KDIGO | 2012 | None |
| 4.51 | We suggest measuring cystatin C in adults with eGFRcreat 45–59 ml/min/1.73 m2 who do not have markers of kidney damage if confirmation of CKD is required.   - If eGFR cys /eGFRcreat-cys is also < 60 ml/min/1.73 m2, the diagnosis of CKD is confirmed. - If eGFRcys/eGFR creat-cys is >/= 60 ml/min/1.73 m2, the diagnosis of CKD is not confirmed. | KDIGO | 2012 | 2C |

| 1. **CKD Management: follow-up** | | | | |
| --- | --- | --- | --- | --- |
|  | **Recommended indicator** | **Source** | **Date** | **Evidence grading** |
| 5.1. | Identify the rate of progression of CKD:   - Obtain a minimum of 3 GFR estimations over a period of not less than 90 days. | NICE CG182  DM | 2014 (2015 update)  2012 | None  Consensus |
| 5.2. | Identify the rate of progression of CKD in people with a new finding of reduced GFR, repeat the GFR within 2 weeks to exclude causes of acute deterioration of GFR – for example, acute kidney injury or starting [renin–angiotensin system antagonist](http://www.nice.org.uk/guidance/cg182/chapter/recommendations#terms-used-in-this-guideline) therapy | NICE CG182 | 2014 (2015 update) | None |
| 5.3. | Assess GFR and albuminuria at least annually in people with CKD. Assess GFR and albuminuria more often for individuals at higher risk of progression, and/or where measurement will impact therapeutic decisions (see figure below). | KDIGO | 2012 | None |
| 5.4. | Acknowledge that small fluctuations in GFR are common and are not necessarily indicative of progression. | NfN  KDIGO | 2015  2012 | None  None |
| 5.5. | Agree the frequency of monitoring (eGFRcreatinine and ACR) with the person with, or at risk of, CKD; bear in mind that CKD is not progressive in many people | NICE CG182 | 2014 (2015 update) | None |
| 5.6. | Use table 2 (cfr infra) to guide the frequency of GFR monitoring for people with, or at risk of, CKD, but tailor it to the person according to:   - the underlying cause of CKD - past patterns of eGFR and ACR (but be aware that CKD progression is often non-linear) - comorbidities, especially heart failure - changes to their treatment (such as [renin–angiotensin–aldosterone system [RAAS] antagonists](http://www.nice.org.uk/guidance/cg182/chapter/recommendations#terms-used-in-this-guideline), NSAIDs and diuretics) - intercurrent illness - whether they have chosen conservative management of CKD | NICE CG182 | 2014 (2015 update) | None |
| 5.7. | Determine the eGFR annually in all risk patients (Grade 2C) | DM | 2012 | 2C |
| 5.8. | Use table to guide the frequency of GFR monitoring for people with, or at risk of, CKD  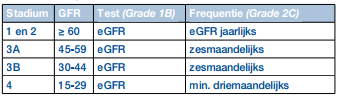   - Tailor the frequency depending on the presence of other risk factors on the development of terminal renal failure and the progression of the eGFR - Let the frequency of detecting proteinuria and complications suspend upon the eGFR, the progression of the eGFR and drug therapy | DM | 2012 | Consensus |
| 5.9. | Determine the corrected albuminuria annually in diabetics (Grade2C). | DM | 2012 | 2C |
| 5.10. | Determine the corrected proteinuria (Grade 2C) in risk patients without diabetes. The frequency of testing remains unclear, but we suggest a maximum of once a year. | DM | 2012 | 2C |
| 5.11. | Define accelerated progression of CKD as and be aware that people with CKD are at increased risk of progression to end-stage kidney disease if they have either of the following:   - a sustained decrease in GFR of 25% or more and a change in GFR category within 12 months or - a sustained decrease in GFR of 15 ml/min/1.73 m2 per year | NICE CG182 | 2014 (2015 update) | None |
| 5.12. | When assessing CKD progression, extrapolate the current rate of decline of GFR and take this into account when planning intervention strategies, particularly if it suggests that the person might need renal replacement therapy in their lifetime | NICE CG182 | 2014 (2015 update) | None |
| 5.13. | Work with people who have any of the following risk factors for CKD progression to optimise their health:   - cardiovascular disease - proteinuria - acute kidney injury - hypertension - diabetes - smoking - African, African-Caribbean or Asian family origin - chronic use of NSAIDs - untreated urinary outflow tract obstruction | NICE CG182 | 2014 (2015 update) | None |
| 5.14. | Monitor people for the development or progression of CKD for at least 2–3 years after acute kidney injury, even if serum creatinine has returned to baseline | NICE CG182 | 2014 (2015 update) | None |
| 5.15. | If the eGFR change is 25% or more, or the change in serum creatinine is 30% or more:   - investigate other causes of a deterioration in renal function, such as volume depletion or concurrent medication (for example, NSAIDs) - if no other cause for the deterioration in renal function is found, stop the renin–angiotensin system antagonist or reduce the dose to a previously tolerated lower dose, and add an alternative antihypertensive medication if required. | NICE CG182 | 2014 (2015 update) | None |
| 5.16. | Identify factors associated with CKD progression to inform prognosis. These include cause of CKD, level of GFR, level of albuminuria, age, sex, race/ethnicity, elevated BP, hyperglycemia, dyslipidemia, smoking, obesity, history of cardiovascular disease, ongoing exposure to nephrotoxic agents, and others. | KDIGO | 2012 | None |
| 5.17. | In patients with established chronic kidney disease and without diabetes, measurement of protein/creatinine ratio may be used to predict risk of progressive disease. | SIGN 103 | 2008 | D |
| 5.18. | Quality of life and psychosocial stressors should be routinely assessed and actively managed psychologically, where indicated. | SIGN 103 | 2008 | GPP |
| 5.19 | Measure GFR and albuminuria once a year or more than once a year in people with CKD. The frequency depends on the degree of kidney damage (cfr table) (not graded). | NfN | 2015 | None |
| 5.20 | Rapid progression is defined as a sustained decline in eGFR of more than 5 ml/min/1.73 m2/yr. | NfN  KDIGO | 2015  2012 | None  None |
| 5.21 | Metabolic complications of kidney failure: Measure serum potassium, calcium, phosphate, PTH and bicarbonate levels, and Hb in patiënts with CKD with a moderate (code orange) to strongly (code red) increased risk. In case of increased PTH, also measure vitamin D, and in the case of reduced Hb also measure ferritin and transferrin saturation. The frequency of these measurements depends on the degree of kidney damage (not graded). | NfN | 2015 | None |
| 5.22 | Sleep apnea is common in patients with CKD (grade 1A). | NfN | 2015 | 1A |
| 5.23 | Because of the beneficial effect of treating sleep apnea syndrome (SAS) on the quality of life in patiënt with CKD, a low threshold in favor of a directed anamnesis and clinical examination for SAS is suggested. | NfN | 2015 | None |
| 5.24 | Offer a renal ultrasound scan to all people with CKD who (grade 1C):   - have accelerated progression of CKD (eGFR decrease of more than 5 ml / min / 1.73 m2 in one year or more than 10 ml / min / 1.73 m2 in five years); - have visible or persistent invisible haematuria - have symptoms of urinary tract obstruction - have a family history of polycystic kidney disease and are aged over 20 years - have a GFR of less than 30 ml/min/1.73 m2 (GFR category G4 or G5) - are considered by a nephrologist to require a renal biopsy. | DM  NICE CG182 | 2012  2014 (2015 update) | 1C  None |
| 5.25 | Advise people with a family history of inherited kidney disease about the implications of an abnormal result before a renal ultrasound scan is arranged for them | NICE CG182 | 2014 (2015 update) | None |

| 1. **CKD Management: Vaccination, risk of hospitalization and mortality** | | | | |
| --- | --- | --- | --- | --- |
|  | **Recommended indicator** | **Source** | **Date** | **Evidence grading** |
| 6.1. | We recommend that all adults with CKD are offered annual vaccination with influenza vaccine, unless  contraindicated. (1B) | KDIGO NfN | 2012  2015 | 1B  1B |
| 6.2. | We recommend that all adults with eGFR < 30 ml/min/1.73 m2 (GFR categories G4-G5) and those at high risk  of pneumococcal infection (e.g., nephrotic syndrome, diabetes, or those receiving immunosuppression) receive  vaccination with polyvalent pneumococcal vaccine unless contraindicated. (1B) | KDIGO | 2012 | 1B |
| 6.3. | We recommend that all adults with CKD who have received pneumococcal vaccination are offered revaccination within 5 years. (1B) | KDIGO | 2012 | 1B |
| 6.4. | We recommend that all adults who are at high risk of progression of CKD and have GFR < 30 ml/min/1.73 m2 (GFR categories G4-G5) be immunized against hepatitis B and the response confirmed by appropriate serological testing. (1B) | KDIGO  NfN | 2012  2015 | 1B  1B |
| 6.5. | Consideration of live vaccine should include an appreciation of the patient’s immune status and should be in  line with recommendations from official or governmental bodies. (Not Graded) | KDIGO | 2012 | None |
| 6.6. | Considering the use of live attenuated vaccine should include an appreciation of the patient’s immune status and drug use (eg. the use of corticosteroids). This should conform to recommendations from official or governmental organs. (Not Graded) | NfN | 2015 | None |
| 6.7. | CKD disease management programs should be developed in order to optimize the community management of  people with CKD and reduce the risk of hospital admission. (Not Graded) | KDIGO | 2012 | None |
| 6.8. | Interventions to reduce hospitalization and mortality for people with CKD should pay close attention to the  management of associated comorbid conditions and cardiovascular disease in particular. (Not Graded) | KDIGO | 2012 | None |

| 1. **CKD Treatment: general** | | | | |
| --- | --- | --- | --- | --- |
|  | **Recommended indicator** | **Source** | **Date** | **Evidence grading** |
| 7.1 | Patients with chronic kidney disease and proteinuria should be treated to reduce proteinuria. | SIGN 103 | 2008 | A |
| 7.2 | Interventions proved to slow the progression of chronic kidney disease include blood pressure control, glycemic control, and reduction of proteinuria with an angiotensin-converting enzyme inhibitor or angiotensin-II receptor blocker. | AAFP | 2005 | A |
| 7.3 | There is insufficient evidence to recommend combining an ACE-I with ARBs to prevent progression of CKD | KDIGO | 2012 | None |
| 7.4 | We suggest that an ARB or ACE-I be used in diabetic adults with CKD and urine albumin excretion 30–300 mg/24 hours (or equivalent*) | KDIGO | 2012 | 2D |
| 7.5 | We recommend that an ARB or ACE-I be used in both diabetic and non-diabetic adults with CKD and urine albumin excretion > 300 mg/24 hours (or equivalent*) | KDIGO | 2012 | 1B |
| 7.6 | Adults with diabetes and persistent albuminuria (ratio of albumin to creatinine > 2.0 mg/mmol for men, > 2.8 mg/mmol for women) should receive an ACE inhibitor or an angiotensin-receptor blocker to delay the progression of chronic kidney disease (grade A). | CMAJ | 2008 | A |
| 7.7 | Patients with nondiabetic kidney disease and a random urine total protein-to-creatinine ratio  > 200 mg /g, and those with diabetic kidney disease, should be treated with an ACE inhibitor or an angiotensin II receptor blocker. | AAFP | 2012 | A |
| 7.8 | Concurrent use of ACE inhibitors and angiotensin II receptor blockers should be avoided because of symptomatic hypotension and worsening kidney function. | AAFP  NICE CG 182 | 2012  2014 (2015 update) | A  None |
| 7.9 | There is insufficient evidence to support a treatment combining an ACE inhibitor with an ARB or combining an ACE inhibitor/ARB with an aldosterone antagonist to reduce progression of CKD. (Not graded). | NfN | 2015 | None |
| 7.10 | Offer a low‑cost [renin–angiotensin system antagonist](http://www.nice.org.uk/guidance/cg182/chapter/recommendations#terms-used-in-this-guideline) to people with CKD and:   - diabetes and an ACR of 3 mg/mmol or more (ACR category A2 or A3) - hypertension and an ACR of 30 mg/mmol or more (ACR category A3) - an ACR of 70 mg/mmol or more (irrespective of hypertension or cardiovascular disease) | NICE CG182 | 2014 (2015 update) | None |
| 7.11 | Treat all patients with a corrected proteinuria > 900 mg / g (100 mg / mmol) with an ACE-I regardless of blood pressure (Grade 1B). | DM | 2012 | 1B |
| 7.12 | An ACE inhibitor (ACE-I) is the preferred antihypertensive agent in all diabetic patients with CKDI and in all patients with a corrected proteinuria > 270 mg / g (30 mg / mmol) (Grade 2B). | DM | 2012 | 2B |
| 7.13 | Treat all diabetic patients with a corrected albuminuria > 20 mg / g (2.5 mg / mmol) in men and > 30 mg / g (3.5 mg / mmol) in women with an ACE-I regardless of blood pressure (Grade 2B). | DM | 2012 | 2B |
| 7.14 | According to the evidence, ACE inhibitors are more effective than other antihypertensive drugs in preventing the progression of kidney disease in diabetic and nondiabetic patients. | AAFP | 2004(b) | C |
| 7.15 | Angiotensin-II receptor antagonists have been shown to reduce proteinuria and the occurrence of kidney failure. | AAFP | 2004(b) | C |
| 7.16 | ACE inhibitors and angiotensin-receptor blockers are the drugs of choice for reducing proteinuria (grade A). | CMAJ | 2008 | A |
| 7.17 | In carefully selected patients, aldosterone-receptor antagonists may decrease proteinuria (grade D). | CMAJ | 2008 | D |
| 7.18 | Patients with chronic kidney disease and type 1 diabetes with microalbuminuria should be treated with an angiotensin converting enzyme inhibitor irrespective of blood pressure. | SIGN 103 | 2008 | A |
| 7.19 | Patients with chronic kidney disease and type 2 diabetes with microalbuminuria should be treated with an angiotensin converting enzyme inhibitor or an angiotensin receptor blocker irrespective of blood pressure | SIGN 103 | 2008 | A |
| 7.20 | Angiotensin converting enzyme inhibitors and/or angiotensin receptor blockers should be used as agents of choice in patients (with or without diabetes) with chronic kidney disease and proteinuria (≥0.5 g/day, approximately equivalent to a protein/creatinine ratio of 50 mg/mmol) in order to reduce the rate of progression of chronic kidney disease and reduce proteinuria | SIGN 103 | 2008 | A |
| 7.21 | Non-dihydropyridine calcium channel blockers should be considered in patients with chronic kidney disease and proteinuria who are intolerant of angiotensin converting enzyme inhibitors or angiotensin receptor blockers. | SIGN 103 | 2008 | A |
| 7.22 | In patients with CKD, an ARB or an ACE inhibitor should be used in case of a strongly increased albuminuria (> 30 mg / mmol or> 300 mg / 24 hours) (grade 1B) and preferably also in case of moderately increased albuminuria (3-30 mg / mmol or 30-300 mg / 24 hours) (grade 2D). | NfN | 2015 | 1B- 2D |
| 7.23 | Angiotensin-Converting Enzyme Inhibitor (ACEi)/Angiotensin Receptor Blocker (ARB) should be used as first-line agent in:   - non-diabetic CKD with urinary protein excretion ≥0.5 g/day in the presence of hypertension. (Grade A) - non-diabetic CKD when urinary protein excretion ≥1.0 g/day irrespective of the presence of hypertension. (Grade A) - all diabetes patients with albuminuria (micro- or macroalbuminuria) irrespective of the CKD stage and presence of hypertension. (Grade A) | MSN | 2011 | A  A  A |
| 7.24 | Monitor serum potassium before and after initiating treatment with an ACE-I or ARB. In case of hyperpotassemia, first rule out any medical cause and then consider a diet to limit potassium intake. | DM | 2012 | 1C |
| 7.25 | Do not routinely offer a renin–angiotensin system antagonist to people with CKD if their pretreatment serum potassium concentration is greater than 5.0 mmol/litre | NICE CG182 | 2014 (2015 update) | None |
| 7.26 | When hyperkalaemia precludes use of renin–angiotensin system antagonists, assessment, investigation and treatment of other factors known to promote hyperkalaemia should be undertaken and the serum potassium concentration rechecked | NICE CG182 | 2014 (2015 update) | None |
| 7.27 | Concurrent prescription of drugs known to promote hyperkalaemia is not a contraindication to the use of renin–angiotensin system antagonists, but be aware that more frequent monitoring of serum potassium concentration may be required | NICE CG182 | 2014 (2015 update) | None |
| 7.28 | Stop renin–angiotensin system antagonists if the serum potassium concentration increases to 6.0 mmol/litre or more and other drugs known to promote hyperkalaemia have been discontinued | NICE CG182 | 2014 (2015 update) | None |
| 7.29 | It is suggested to prevent serum potassium from exceeding >5.5 mmol / l to prevent the occurrence of cardiac arrhythmia. Both increased potassium (> 5.5 mmol / l) and reduced / low normal potassium (<4.0 mmol / l) are associated with mortality and cardiovascular endpoints (grade 2B). | NfN | 2015 | 2B |
| 7.30 | To reduce the potassium levels the following measures are advised (grade 1C):   - a dietary potassium limitation between 2000-3000 mg [50-75 mmol] per day - adjustment of potassium-increasing medication - correction of metabolic acidosis - the use of potassium binders (preferably non-sodium binders) (if necessary) | NfN | 2015 | 1C |
| 7.31 | We suggest that in people with CKD and serum bicarbonate concentrations < 22 mmol/l treatment with oral bicarbonate supplementation be given to maintain serum bicarbonate within the normal range, unless contraindicated. | KDIGO | 2012 | 2B |
| 7.32 | In patients with CKD and a serum bicarbonate <20 mmol / L treatment should be initiated using an oral bicarbonate to keep the serum bicarbonate level within the normal range (grade 2B) | NfN | 2015 | 2B |
| 7.33 | Consider oral sodium bicarbonate supplementation for people with a GFR less than 30 ml/min/1.73 m^2^ (GFR category G4 or G5) and a serum bicarbonate concentration of less than 20 mmol/litre. | NICE CG182 | 2014 (2015 update) | None |
| 7.34 | Consider oral sodium bicarbonate supplementation for patients with metabolic acidosis (Grade 1B). | DM | 2012 | 1B |

| 1. **CKD Treatment: Patient education and information** | | | | |
| --- | --- | --- | --- | --- |
|  | **Recommended indicator** | **Source** | **Date** | **Evidence grading** |
| 8.1 | The patient plays an important role in the self-management of CKD. | EBM | 2017 | None |
| 8.2 | Involve patients with CKD in the elaboration of information- and education programs to (GPP). | EBM | 2017 | GPP |
| 8.3 | Ofer tailored education and support programs in the self-management of CKD patients (GPP). Referral to a specialized nurse is recommended in order to ameliorate understanding of their condition, to ameliorate compliance to lifestyle changes and drug treatment (GRADE 1C). These measures are taken to stabilize parametres and to preserve renal function as long as possible (GPP). | EBM | 2017 | GPP 1C  GPP |
| 8.4 | Provide the following support: information for patients with CKD about their condition, a program for 'shared decision making', support for self-management (eg blood pressure, smoking cessation, exercise, diet and medication) and support in making a well informed choice (Grade2B). | EBM | 2017 | 2B |
| 8.5 | Ensure that systems are in place to:   - inform people with CKD of their diagnosis - enable people with CKD to share in decision-making about their care - support self-management (this includes providing information about blood pressure, smoking cessation, exercise, diet and medicines) and enable people to make informed choices | NICE CG182 | 2014 (2015 update) | None |
| 8.6 | Offer people with CKD education and information tailored to the severity and cause of CKD, the associated complications and the risk of progression | NICE CG182 | 2014 (2015 update) | None |
| 8.7 | When developing information or education programmes, involve people with CKD in their development from the outset. The following topics are suggested.  What is CKD and how does it affect people?  What questions should people ask about their kidneys?  What treatments are available for CKD, what are their advantages and disadvantages and what complications or side effects may occur as a result of treatment/medication?   - What can people do to manage and influence their own condition? - In what ways could CKD and its treatment affect people's daily life, social activities, work opportunities and financial situation, including benefits and allowances available? - How can people cope with and adjust to CKD and what sources of psychological support are available? - When appropriate, offer information about renal replacement therapy (such as the frequency and length of time of dialysis treatment sessions or exchanges and pre-emptive transplantation) and the preparation required (such as having a fistula or peritoneal catheter). - Conservative management and when it may be considered | NICE CG182 | 2014 (2015 update) | None |
| 8.8 | Provide patients with CKD information about the following topics (based on consensus):   - What is CKD and how does it affect the patient?   - What is the function of the kidneys?   - How do you check the kidneys?   - Why should the kidneys be checked?   - What does the result of the kidney test mean?   - Which products and situations can damage the kidneys? - How to prepare for a consultation with the general practitioner or nephrologist? - What are the possible treatments for CKD? - What can the patient do to monitor and influence his/her condition (with(out) the help of a doctor) ? | DM | 2012 | consensus |
| 8.9 | Offer people with CKD high-quality information or education programmes as appropriate to the severity of their condition to allow time for them to fully understand and make informed choices about their treatment | NICE CG182 | 2014 (2015 update) | None |
| 8.10 | Healthcare professionals working with people with CKD should take account of the psychological aspects of coping with the condition and offer access to appropriate support – for example, support groups, counselling or a specialist nurse. | NICE CG182 | 2014 (2015 update) | None |
| 8.11 | Give people access to their medical data (including diagnosis, comorbidities, test results, treatments and correspondence) through information systems, such as [Renal PatientView](https://www.patientview.org/), to encourage and help them to self-manage their CKD | NICE CG182 | 2014 (2015 update) | None |
| 8.12 | The delivery of a psychologically informed, pre-dialysis psychoeducation programme is recommended for all patients with progressive chronic kidney disease at any stage who will eventually require renal replacement therapy. | SIGN 103 | 2008 | B |
| 8.13 | Healthcare professionals providing information and education programmes should ensure they have specialist knowledge about CKD and the necessary skills to facilitate learning (GPP). | EBM NICE CG182 | 2017 2014 (2015) | GPP None |

| 1. **CKD Treatment: Lifestyle and diet** | | | | |
| --- | --- | --- | --- | --- |
|  | **Recommended indicator** | **Source** | **Date** | **Evidence grading** |
| 9.1 | Patients with CKD should be encouraged to (GRADE 1B):   - undertake physical activity compatible with cardiovascular health and tolerance (aiming for at least 30 minutes 5 times per week) - stop smoking - obtain or maintain a healthy weight (BMI 20 to 25, depending on country-specific demographics). - limit the alcohol intake (only mentioned in NfN) | EBM  NICE CG182  SIGN 103  KDIGO  NfN  MSN | 2017  2014 (2015 update)  2008  2012  2015  2011 | 1B  None  GPP  1D  1D  B |
| 9.2 | Encourage patients with CKD to exercise (Grade 1B), to maintain a healthy weight (Grade 1C) and to stop smoking (Grade 1B). | DM | 2012 | 1B-1C-1B |
| 9.3 | Nutritional status (height, weight, body mass index, percentage weight loss) should be monitored in all patients with chronic kidney disease at stage 3 or higher. | SIGn 103 | 2008 | D |
| 9.4 | People with chronic kidney disease with a waist circumference ≥94 cm in men or ≥80 cm in women should be considered for weight management with the involvement of an appropriately qualified dietitian. | SIGN 103 | 2008 | GPP |
| 9.5 | Smoking cessation should be encouraged to reduce the risk of developing chronic kidney disease and end-stage renal disease, and to reduce the risk of cardiovascular disease (grade D). | CMAJ | 2008 | D |
| 9.6 | Weight reduction   - Obese (BMI > 30.0 kg/m2 ) and overweight (BMI 25.0–29.9 kg/m2 ) people should be encouraged to reduce their BMI to lower their risk of chronic kidney disease and end-stage renal disease (grade D). - Maintenance of a health body weight (BMI 18.5–24.9 kg/m2 ; waist circumference < 102 cm for men, < 88 cm for women) is recommended to prevent hypertension (grade C) or to reduce blood pressure in those with hypertension (grade B). All overweight people with hypertension should be advised to lose weight (grade B). | CMAJ | 2008 | D |
| 9.7 | Exercise   - People without hypertension (to reduce the possibility of becoming hypertensive) or those with hypertension (to lower their blood pressure) should be encouraged to accumulate 30–60 minutes of moderate-intensity dynamic exercise (walking, jogging, cycling or swimming) 4–7 days per week (grade D). Higher intensities of exercise are no more effective. | CMAJ | 2008 | D |
| 9.8 | Alcohol intake   - To reduce blood pressure, alcohol consumption in both normotensive and hypertensive people should be in accordance with Canadian guidelines for low-risk drinking. Healthy adults should limit alcohol consumption to 2 drinks or less per day, and consumption should not exceed 14 standard drinks per week for men and 9 standard drinks per week for women (grade B). | CMAJ | 2008 | - B |
| 9.9 | Protein-controlled diet, as well as weight reduction (for patients with an elevated body mass index), may provide some benefit in decreasing proteinuria (grade D). | CMAJ | 2008 | D |
| 9.10 | If patients experience a reduction in exercise capacity which impacts on their daily life, they should have access to an appropriately qualified physiotherapist. | SIGN 103 | 2008 | GPP |
| 9.11 | All patients with chronic kidney disease who have problems with the activities of daily living should have access to an occupational therapist. | SIGN 103 | 2008 | GPP |
| 9.12 | If dietary advice on potassium, phosphate, protein, calorie and salt seems necessary in patients with advanced CKD, consult the nephrologist and refer to a specialized dietitian (Grade 2C). | DM | 2012 | 2C |
| 9.13 | For CNI stage 1 to 3, no specific dietary advice other than the healthy diet recommended to the general population is required, except for patients with hypertension (low salt) or hypercholesterolemia (low saturated fatty acids) (GPP). | EBM | 2017 | GPP  GPP |
| 9.14 | Offer dietary advice about potassium, phosphate, calorie and salt intake appropriate to the severity of CKD | NICE CG182 | 2014 (2015 update) | None |
| 9.15 | Where dietary intervention is agreed this should occur within the context of education, detailed dietary assessment and supervision to ensure malnutrition is prevented | NICE CG182 | 2014 (2015 update) | None |
| 9.16 | We recommend that individuals with CKD receive expert dietary advice and information in the context of an  education program, tailored to severity of CKD and the need to intervene on salt, phosphate, potassium, and  protein intake where indicated. (1B) | NfN  KDIGO | 2015  2012 | 1B  1B |
| 9.17 | Complex diets for some patients with severe CKD require specialized guidance by a dietician to prevent dietary errors and/or malnutrition. Dietary advice about potassium and phosphate intake tailored to CKD stage is made by an appropriately qualified dietitian (Grade 1B). | EBM | 2017 | 1B |
| 9.18 | Patients exhibiting signs of malnutrition (body mass index <20 kg/m2 or >30 kg/m2 or unintentional weight loss of >10% in six months) should be referred to an appropriately qualified dietitian. | SIGN 103 | 2008 | GPP |
| 9.19 | In the absence of other recognised medical causes patients with chronic kidney disease and consistently raised serum potassium levels should be managed with the involvement of an appropriately qualified dietitian. | SIGN 103 | 2008 | GPP |
| 9.20 | If dietary advice for patients with CKD is needed, including energy intake, protein, sodium/salt, potassium and phosphor, it is indicated to consult a nephrologist and refer the patient to an appropriately qualified dietitian (Grade 1B). Giving dietary advice to patients with CKD is tailored to the patient and depends on the CKD stage and blood levels of the patient at that time. | EBM | 2017 | 1B |
| 9.21 | Do not offer low-protein diets (dietary protein intake less than 0.6–0.8 g/kg/day) to people with CKD | NICE CG182 | 2014 (2015 update) | None |
| 9.22 | We suggest lowering protein intake to 0.8 g/kg/day in adults with diabetes (2C) with appropriate education. | KDIGO | 2012 | 2C |
| 9.23 | We suggest lowering protein intake to 0.8 g/kg/day in adults without diabetes (2B) and GFR < 30 ml/min/ 1.73 m2 (GFR categories G4-G5), with appropriate education. | KDIGO  NfN | 2012  2015 | 2B  2B |
| 9.24 | A protein-controlled diet (0.80–1.0 g/kg/d) is recommended for adults with chronic kidney disease (grade D). | CMAJ | 2008 | D |
| 9.25 | Dietary protein restrictions (<0.8 g/kg/day) are not recommended in patients with early stages of chronic kidney disease (stages 1-3). | SIGN 103 | 2008 | A |
| 9.26 | Bij CNI-stadium 3 tot en met 5 is een voeding met adequate energie-inname (30-35kcal/kg/dag) en een eiwit-inname van 0,8 g/kg/dag aanbevolen (GRADE 2B). Een eiwitinname lager dan 0,6 g/kg/dag is af te raden vanwege het risico op energieeiwitondervoeding (GRADE 2B); | EBM | 2017 | 2B |
| 9.27 | In stage 4 chronic kidney disease patients high protein intake (>1.0 g/kg) is not recommended. | SIGN 103 | 2008 | GPP |
| 9.28 | Low protein diet (0.6 - 0.8 g/kg/day) with adequate energy intake (30 - 35 kcal/kg/day) may be given to patients with chronic kidney disease Stage 3 - 5. (Grade B) | MSN | 2011 | B |
| 9.29 | We suggest avoiding high protein intake (> 1.3 g/kg/day) in adults with CKD at risk of progression. | KDIGO  NfN | 2012  2015 | 2C  2C |
| 9.30 | Dietary protein restriction of < 0.70 g/kg/day should include careful monitoring of clinical and biochemical markers of nutritional deficiencies (grade D). | CMAJ | 2008 | D |
| 9.31 | Dietary protein restriction should be supervised by a dietitian. (Grade B) | MSN | 2011 | B |
| 9.32 | For patients with stage 1-4 chronic kidney disease and hypertension a reduction in sodium (<2.4 g/day or <100 mmol/day which is equivalent to <6 g of salt) is recommended as part of a comprehensive strategy to lower blood pressure and reduce cardiovascular risk. | SIGN 103 | 2008 | B |
| 9.33 | Dietary salt intake   - To prevent hypertension, a dietary sodium intake of < 100 mmol/day is recommended, in addition to a well-balanced diet (grade B). - Patients with hypertension should limit their dietary sodium intake to 65–100 mmol/day (grade B). | CMAJ | 2008 | B |
| 9.34 | Sodium restriction (total intake <2,400 mg/day) should be initiated in patients with chronic kidney disease. (Grade C) | MSN | 2011 | C |
| 9.35 | We recommend lowering salt intake to < 90mmol (< 2 g) per day of sodium (corresponding to 5 g of sodium  chloride) in adults, unless contraindicated (see rationale). (1C) | NfN  KDIGO  EBM | 2015  2012  2017 | 1C  1C  1C |
| 9.36 | Salt substitutes that contain high amounts of potassium salts should not be used in patients with chronic kidney disease. | SIGN 103 | 2008 | GPP |

| 1. **CKD Treatment: uricemia** | | | | |
| --- | --- | --- | --- | --- |
|  | **Recommended indicator** | **Source** | **Date** | **Evidence grading** |
| 10.1 | There is insufficient evidence to support or refute the use of agents to lower serum uric acid concentrations in people with CKD and either symptomatic or asymptomatic hyperuricemia in order to delay progression of CKD. | (Not Graded)  . | 2015  2012 | None  None |

| 1. **CKD Treatment: diabetes and glycemic control** | | | | |
| --- | --- | --- | --- | --- |
|  | **Recommended indicator** | **Source** | **Date** | **Evidence grading** |
| 11.1 | We suggest that target HbA1c be extended > 7.0% (53 mmol/mol) in individuals with comorbidities or limited life expectancy and risk of hypoglycemia. | KDIGO | 2012 | 2C |
| 11.2 | We recommend a target hemoglobin A1c (HbA1c) of ~7.0% (53 mmol/mol) to prevent or delay progression of  the microvascular complications of diabetes, including diabetic kidney disease. (1A) | NfN  KDIGO | 2015  2012 | 1A  1A |
| 11.3 | We recommend not treating to an HbA1c target of <7.0% (<53 mmol/mol) in patients at risk of hypoglycemia. (1B) | NfN  KDIGO | 2015  2012 | 1B  AB |
| 11.4 | We recommend not striving for a HbA1c target of <7.0% (< 53 mmol/mol) in patients with co-mobidity or limited life expectancy and risk of hypoglycemia (grade 2C). | NfN | 2015 | 2C |
| 11.5 | The target HbA1c should be ≤7% in patients with diabetes but this should be individualised according to co-morbidities. (Grade A) | MSN | 2011 | A |
| 11.6 | To prevent progression of nephropathy in patients with diabetes mellitus, the American Diabetes Association recommends glycemic control, with the goal being an HbA1C concentration <7%, fasting plasma glucose 4–7 mmol/L) | AAFP  CMAJ | 2004(b)  2008 | C  B |
| 11.7 | Use of metformin in type 2 diabetes mellitus:   - Metformin is recommended for most patients with type 2 diabetes with stage 1 or 2 chronic kidney disease who have stable renal function that has been unchanged over the past 3 months (grade A). - Metformin may be continued in patients with stable stage 3 chronic kidney disease (grade B). - Metformin should be stopped if there are acute changes in renal function or during periods of illnesses that could precipitate such changes (e.g., gastrointestinal upset or dehydration) or cause hypoxia (e.g., cardiac or respiratory failure). Particular care should be taken for patients also taking ACE inhibitors, angiotensin-receptor blockers, nonsteroidal anti-inflammatory drugs or diuretics, or after intravenous contrast administration because the risk of acute renal failure, and thus accumulation of lactic acid, is greatest for these patients | CMAJ | 2008 | A  B  Clinical recommendation |
| 11.8 | In the context of drug management and patient safety, we recommend the use of metformin in patients with an eGFR >45ml/min./1.73m²; Metformin use has to be evaluated if eGFR is between 30-44ml/min./1.73m². Metformin has to be avoided with an eGFR <30 ml/min./1.73m² (GRADE 1C); | DM | 2012 | 1C |
| 11.9 | Metformin and sulfonylurea are used with caution in patients with CKD. (Grade 1C) | DM | 2012 | 1C |
| 11.10 | Choice of other glucose-lowering agents   - Tailor the choice of other glucose-lowering agents (including insulin) to the individual patient, the level of renal function and comorbidity (grade D opinion). - Risk of hypoglycemia should be assessed regularly for patients taking insulin or insulin secretagogues. These patients should be taught how to recognize, detect and treat hypoglycemia (grade D opinion). - Short-acting sulfonylureas (e.g., gliclazide) are preferred over long- acting agents for patients with chronic kidney disease | CMAJ | 2008 | D  D |
| 11.11 | Be extra vigilant for the risk of hypoglycemia at initiation or augmenting insulin dosage (Grade 1C). | DM | 2012 | 1C |
| 11.12 | In people with CKD and diabetes, glycemic control should be part of a multifactorial intervention strategy addressing blood pressure control and cardiovascular risk, promoting the use of angiotensin-converting enzyme inhibition or angiotensin receptor blockade, statins, and antiplatelet therapy where clinically indicated | KDIGO  CMAJ | 2012  2008 | None  A |
| 11.13 | We suggest that adults with CKD and diabetes are offered regular podiatric assessment. | KDIGO | 2012 | 2B |

| 1. **CKD Treatment: dyslipidemia** | | | | |
| --- | --- | --- | --- | --- |
|  | **Recommended indicator** | **Source** | **Date** | **Evidence  grading** |
| 12.1 | Screening for dyslipidemie:   - A fasting lipid profile (total cholesterol, LDL cholesterol, HDL cholesterol and triglyceride) should be measured in adults with stage 1–3 chronic kidney disease (grade A). - A fasting lipid profile should be measured in adults with stage 4 chronic kidney disease only if the results would influence the decision to initiate or alter lipid- modifying treatment (grade D). | CMAJ | 2008 | A  D |
| 12.2 | Frequency of lipid-profile measurement   - Lipid profiles should be measured after an overnight fast (ideally ≥ 12 h duration) (grade A). - Total cholesterol, LDL cholesterol, HDL cholesterol and triglycerides should be measured (grade A). - Fasting lipid profiles should be measured no sooner than 6 weeks after initiation or change in pharmacologic therapy. Thereafter, lipid profiles should be monitored every 6–12 months if the results could influence subsequent therapeutic decisions (grade D). | CMAJ | 2008 | A  A  D |
| 12.3 | In adults with CKD it is recommended to determine a one time lipid profile (total cholesterol, LDL cholesterol, HDL cholesterol, triglycerides) (graad 1C). | NfN | 2015 | 1C |
| 12.4 | In the majority of adults with CKD repeating measurement of lipid profile is unnecessary (Not graded). | NfN | 2015 | None |
| 12.5 | Offer atorvastatin 20 mg for the primary or secondary prevention of CVD to people with CKD.   - Increase the dose if a greater than 40% reduction in non‑HDL cholesterol is not achieved (see recommendation 1.3.28) and eGFR is 30 ml/min/1.73 m^2^ or more. - Agree the use of higher doses with a renal specialist if eGFR is less than 30 ml/min/1.73 m^2^ | NICE CG181 | 2014 (2016 update) | None |
| 12.6 | In patients with stage 1–3 chronic kidney disease, clinicians should consider titrating the dose of statin according to lipid guidelines for the general population (grade B). | CMAJ | 2008 | B |
| 12.7 | Clinicians should consider initiating statin therapy for patients with stage 4 chronic kidney disease and titrating the dose to achieve an LDL cholesterol level < 2.0 mmol/L and a ratio of total cholesterol to HDL cholesterol < 4.0 mmol/L (grade B). | CMAJ | 2008 | B |
| 12.8 | Gemfibrozil (1200 mg daily) may be considered as an alternative to statin treatment for patients with chronic kidney disease (stage 1–3) who are at intermediate or high cardiovascular risk with concomitant low levels of HDL cholesterol (< 1.0 mmol/L) (grade B). | CMAJ | 2008 | B |
| 12.9 | Fasting triglycerides > 10 mmol/L at any stage of chronic kidney disease should be treated by recommending lifestyle changes and adding gemfibrozil or niacin, as required to reduce the risk of acute pancreatitis (grade D). Current data do not support treating hypertriglyceridemia as a strategy to reduce cardiovascular risk (grade A). | CMAJ | 2008 | A |
| 12.10 | Monitoring for medication adverse effects   - Serial monitoring of creatinine kinase and alanine aminotransferase is not required for asymptomatic patients with chronic kidney disease (any stage) taking a low to moderate dose of statin (≤ 20 mg/d of simvastatin or atorvastatin, or an equivalent dose of another statin) (grade A). - Serial creatinine kinase and alanine aminotransferase should be measured every 3 months for patients with stage 4 chronic kidney disease who are taking a moderate to high dose of statin (≥ 40 mg/d of simvastatin or atorvastatin, or an equivalent dose of another statin) (grade D). - A statin and fibrate should not be coadministered to patients with stage 4 chronic kidney disease because of the risk of rhabdomyolysis (grade D). - Gemfibrozil is safe to use for patients with chronic kidney disease. Other fibrate preparations (e.g., fenofibrate) should be avoided or the dose significantly reduced for patients with stage 2–4 chronic kidney disease because of an increased risk of toxicity (grade D). | CMAJ | 2008 | - A - D - D - D |
| 12.11 | In adults aged ≥50 years with an eGFR <60 ml/min/1.73 m2 treatment with statines or a combination statine/ezetimibe is advised (graad 1A). | NfN | 2015 | 1A |
| 12.12 | In adults aged ≥50 years with CKD and an eGFR <60 ml/min/1.73 m2 treatment with statines is advised (graad 1B). | NfN | 2015 | 1B |
| 12.13 | In adults aged 18–49 years with CKD treatment with statines is suggested if one or more of the following conditions apply: (graad 2A)   - history of coronary heart disease (myocardal infarction or previous coronary revascularization) - diabetes mellitus - previous ischemic stroke - estimated 10 year risk of fatal coronary disease or non-fatal myocardial infarction >10% | NfN | 2015 | 2A |
| 12.14 | Statin should be offered to patients with chronic kidney disease for primary and secondary prevention of cardiovascular events | MSN | 2011 | A |
| 12.15 | ACP recommends that clinicians choose statin therapy to manage elevated low-density lipoprotein in patients with stage 1 to 3 chronic kidney disease. | ACP/AAFP  Annals  AAFP  CMAJ | 2013  2013  2014  2008 | Strong recommendation, moderate -quality evidence (x3)  A |
| 12.16 | Statin therapy should be considered in all patients with stage 1-3 chronic kidney disease, with a predicted 10-year cardiovascular risk ≥20%. | SIGN 103 | 2008 | B |
| 12.17 | The most recent guidelines from the NKF K/DOQI recommend treating dyslipidemia aggressively in patients with chronic kidney disease. The goals are an LDL cholesterol level <100 mg/dL (2.60 mmol per L) and a triglyceride level <200 mg/dL (2.26 mmol per L). | AAFP | 2004(b) | C |
| 12.18 | A low-density lipoprotein goal of < 100 mg/dL (2.60 mmol/L) is recommended for patients with chronic kidney disease, because these patients are statistically at highest risk for cardiovascular disease. | AAFP | 2005 | C |

| 1. **CKD Treatment: Hypertension** | | | | |
| --- | --- | --- | --- | --- |
|  | **Recommended indicator** | **Source** | **Date** | **Evidence  grading** |
| 13.1 | In patients without diabetes blood pressure should be targeted to less <130/80 mmHg (grade C) | CMAJ  AAFP | 2008  2004(b) | C  C |
| 13.2 | Target BP should be <130/80 (SBP range 120 - 129) mmHg in patients with proteinuria ≥1 g/day. (Grade A) | MSN SIGN | 2011 2008 | A  A |
| 13.3 | A blood pressure goal of 130/80 mm Hg is recommended in patients with normal urinary albumin concentrations, and a blood pressure goal of 125/75 mm Hg is recommended in patients with proteinuria ≥1 g/24 hours. | AAFP | 2005 | B |
| 13.4 | Target BP should be <130/80 (SBP range 120 - 129) mmHg in patients with diabetes. (Grade B) | MSN CMAJ | 2011  2008 | B  C-B |
| 13.5 | Strive for a systolic blood pressure between 120 en 139 mmHG in all patients with CKD and a diastolic pressure between 60 en 89 mmHg (Grade 1B). | DM | 2012 | 1B |
| 13.6 | In patients with a strongly increased albuminuria (ACR >30 mg/mmol (or >300 mg/24 uur) aim to keep the bloodpressure ≤130/80 mmHg, in which antihypertensive drugs of first choice to achieve these goals are ACE inhibitors or ARB's(graad 1B). | NfN  KDIGO | 2015  2012 | 1B  2D |
| 13.7 | We suggest that in both diabetic and non-diabetic adults with CKD and with urine albumin excretion of ⋝30 mg/24 hours (or equivalent*) whose office BP is consistently >130 mm Hg systolic or >80 mm Hg diastolic be treated with BP-lowering drugs to maintain a BP that is consistently ⋜130 mm Hg systolic and ⋜80 mm Hg diastolic. (2D) | NfN  KDIGO | 2015  20121 | 2D  2D |
| 13.8 | In people with CKD and diabetes, and also in people with ACR ≥70 mg/mmol, aim to keep the systolic blood pressure <130 mmHg (target range 120–129 mmHg) and the diastolic blood pressure <80 mmHg | NICE CG182 | 2014 (2015 update) | None |
| 13.9 | We recommend that in both diabetic and non-diabetic adults with CKD and urine albumin excretion o30 mg/ 24 hours (or equivalent*) whose office BP is consistently 4140 mm Hg systolic or 490 mm Hg diastolic be treated with BP-lowering drugs to maintain a BP that is consistently r140 mm Hg systolic and r90 mm Hg diastolic. (1B) | NfN  KDIGO  NICE CG182  MSN | 2015  2012  2014 (2015 update)  2011 | 1B  1B  None  A |
| 13.10 | Inquire about postural dizziness and check for postural hypotension regularly when treating CKD patients with BP-lowering drugs. (Not Graded) | NfN  KDIGO | 2015  2012 | None  None |
| 13.11 | Tailor BP treatment regimens in elderly patients with CKD by carefully considering age, comorbidities and other therapies, with gradual escalation of treatment and close attention to adverse events related to BP treatment, including electrolyte disorders, acute deterioration in kidney function, orthostatic hypotension and drug side effects. (Not Graded) | NfN  KDIGO | 2015  2012 | None  None |
| 13.12 | For patients without diabetes with proteinuric chronic kidney disease (urine ratio of albumin to creatinine ≥ 30 mg/mmol), antihypertensive therapy should include an ACE inhibitor (grade A) or an angiotensin-receptor blocker in cases of intolerance to ACE inhibitors (grade D). | CMAJ | 2008 | A en D |
| 13.13 | For patients without diabetes with nonproteinuric chronic kidney disease (albumin to creatinine ratio < 30 mg/mmol), antihypertensive therapy should include either an ACE inhibitor (grade B), an angiotensin-receptor blocker (grade B), a thiazide diuretic (grade B), a β-blocker (patients aged 60 years or less; grade B) or a long-acting calcium-channel blocker (grade B) | CMAJ | 2008 | B-B-B |
| 13.14 | Patients with diabetes antihypertensive therapy should include either an ACE inhibitor (grade A) or an angiotensin-receptor blocker (grade A). | CMAJ | 2008 | A |
| 13.15 | In patients with large-vessel renal vascular disease renovascular hypertension should be treated in the same manner as for nondiabetic, nonproteinuric chronic kidney disease. Caution should be taken with the use of an ACE inhibitor or an angiotensin-receptor blocker because of the risk of acute renal failure (grade D). | CMAJ | 2008 | D |
| 13.16 | Any class of antihypertensive agents can be used to treat hypertension in chronic kidney disease (CKD) patients without proteinuria. (Grade C) The choice will depend on the patient’s co-morbidity. | MSN | 2011 | C |
| 13.17 | ACP recommends that clinicians select pharmacologic therapy that includes either an ACEi (moderate-quality evidence) or an ARB (high-quality evidence) in patients with hypertension and stage 1 to 3 chronic kidney disease. | ACP/AAFP  Annals  AAFP | 2013  2013  2014 | (Grade: strong recommendation) |

| 1. **CKD Treatment: cardiovascular disease** | | | | |
| --- | --- | --- | --- | --- |
|  | **Recommended indicator** | **Source** | **Date** | **Evidence  grading** |
| 14.1 | We recommend that the level of care for ischemic heart disease offered to people with CKD should not be prejudiced by their CKD. | KDIGO | 2012 | 1A |
| 14.2 | We suggest that the level of care for heart failure offered to people with CKD should be the same as is offered to those without CKD. | KDIGO | 2012 | 2A |
| 14.3 | We suggest that adults with CKD at risk for atherosclerotic events be offered treatment with antiplatelet agents unless there is an increased bleeding risk that needs to be balanced against the possible cardiovascular benefits. | KDIGO | 2012 | 2B |
| 14.4 | In people with CKD and heart failure, any escalation in therapy and/or clinical deterioration should promptmonitoring of eGFR and serum potassium concentration. | KDIGO | 2012 | None |
| 14.5 | In people with GFR < 60 ml/min/1.73 m2 (GFR categories G3a-G5), we recommend that serum concentrations of BNP/NT-proBNP be interpreted with caution and in relation to GFR with respect to diagnosis of heart failure and assessment of volume status. | KDIGO | 2012 | 1B |
| 14.6 | In people with GFR < 60 ml/min/1.73 m2 (GFR categories G3a-G5), we recommend that serum concentrations of troponin be interpreted with caution with respect to diagnosis of acute coronary syndrome. | KDIGO | 2012 | 1B |
| 14.7 | We recommend that people with CKD presenting with chest pain should be investigated for underlying cardiac disease and other disorders according to the same local practice for people without CKD (and subsequent treatment should be initiated similarly). | KDIGO | 2012 | 1B |
| 14.8 | We suggest that clinicians are familiar with the limitations of non-invasive cardiac tests (e.g., exercise electrocardiography [ECG], nuclear imaging, echocardiography, etc.) in adults with CKD and interpret the results accordingly. | KDIGO | 2012 | 2B |
| 14.9 | We recommend that adults with CKD be regularly examined for signs of peripheral arterial disease and be considered for usual approaches to therapy. | KDIGO | 2012 | 1B |
| 14.10 | Offer antiplatelet drugs (acetylsalicylic acid) to people with CKD for the secondary prevention of cardiovascular disease, but be aware of the increased risk of bleeding. | NfN  MSN  NICE CG 182 | 2015  2011  2014 (2015 update) | 1B  B  None |
| 14.11 | There is insufficient evidence to support the use of acetylsalicylic acid or other new antiplatelet agents in primary cardiovascular prevention (not graded). | NfN | 2015 | None |
| 14.12 | Combination of clopidogrel with aspirin should be avoided in patients with CKD unless compelling indications are present. (Grade B) | MSN | 2011 | B |
| 14.13 | Consider apixaban in preference to warfarin in people with a confirmed eGFR of 30–50 ml/min/1.73 m^2^ and non-valvular atrial fibrillation who have 1 or more of the following risk factors:   - prior stroke or transient ischaemic attack - age 75 years or older - hypertension - diabetes mellitus - symptomatic heart failure | NICE CG182 | 2014 (2015 update) | None |
| 14.14 | Do not advise any to take plant stanols or sterols for the prevention of CVD in people with CKD | NICE CG181 | 2014 (2016 update) | None |
| 14.15 | Do not routinely offer fibrates for the prevention of CVD to people with CKD | NICE CG181 | 2014 (2016 update) | None |
| 14.16 | Do not offer nicotinic acid (niacin) for the prevention of CVD to people witch CKD | NICE CG181 | 2014 (2016 update) | None |
| 14.17 | Do not offer a bile acid sequestrant (anion exchange resin) for the prevention of CVD to patients with CKD | NICE CG181 | 2014 (2016 update) | None |
| 14.18 | Do not offer omega‑3 fatty acid compounds for the prevention of CVD to people with CKD | NICE CG181 | 2014 (2016 update) | None |

| 1. **CKD Treatment: anemia** | | | | |
| --- | --- | --- | --- | --- |
|  | **Recommended indicator** | **Source** | **Date** | **Evidence  grading** |
| 15.1 | Diagnose anemia in adults and children >15 years with CKD when the Hb concentration is <13.0 g/dl (<130 g/l) in males and <12.0 g/dl (<120 g/l) in females. | KDIGO | 2012 | None |
| 15.2 | Anemia is defined as a hemoglobin level of < 135 g/L for adult men and < 120 g/L for adult women (grade D, opinion). | CMAJ | 2008 | D |
| 15.3 | To identify anemia in people with CKD measure Hb concentration (Not Graded):   - when clinically indicated in people with GFR 60 ml/min/1.73 m2 (GFR categories G1-G2); - at least annually in people with GFR 30–59 ml/min/1.73 m2 (GFR categories G3a-G3b); - at least twice per year in people with GFR 30 ml/min/1.73 m2 (GFR categories G4-G5) | KDIGO | 2012 | None |
| 15.4 | To identify anemia, measure hemoglobine (Hb) concentration in patients with CKD stage 3B, 4 en 5 (GFR <45 ml/min/1,73 m2 ) If not yet determined. Determine the subsequent frequency of testing by the measured values and the clinical circumstances.(Grade 1C) | DM | 2012 | 1C |
| 15.5 | anemia in patients with stage 3-5 chronic kidney disease:  Consider testing patients with a hemoglobin level < 120 g/L for the following (grade D, opinion): hemoglobin level, leukocyte count and differential, platelet count, erythrocyte indices, absolute reticulocyte count, serum ferritin and transferrin saturation. | CMAJ | 2008 | D |
| 15.6 | If not already measured, check the haemoglobin level in people with a GFR < 45 ml/min/1.73 m^2^ (GFR category G3b, G4 or G5) to identify anaemia (haemoglobin less than 110 g/litre [11.0 g/dl]). Determine the subsequent frequency of testing by the measured value and the clinical circumstances. | NICE CG182 | 2014 (2015 update) | None |
| 15.7 | Offer EPO and iron to treat anemia of renal origin (Grade 1C) | DM | 2012 | 1C |
| 15.8 | A Erythropoiesis stimulating agents should be considered in all patients with anaemia of chronic kidney disease to improve their quality of life. | SIGN 103 | 2008 | A |
| 15.9 | Anemia in patients with stage 3-5 chronic kidney disease: For patients with anemia and adequate iron stores, erythropoiesis-stimulating agents should be initiated if their hemoglobin level falls below 100 g/L (grade D, opinion). | CMAJ | 2008 | D |
| 15.10 | Anemia in patients with stage 3-5 chronic kidney disease: For patients receiving erythropoiesis-stimulating agents, the target hemoglobin level should be 110 g/L (grade A). An acceptable hemoglobin range is 100–120 g/L. | CMAJ | 2008 | A |
| 15.11 | In patients with chronic kidney disease treated with erythropoiesis stimulating agents the haemoglobin should normally be kept between 100 g/l and 120 g/l. | SIGN 103 | 2008 | GPP |
| 15.12 | Hemoglobin goals should not exceed 11 g per dL (110g/L) in patients receiving erythropoiesis-stimulating agents due to the risk of major cardiovascular events. | AAFP | 2012 | A |
| 15.13 | It is suggested not to initiate treatment with ESA when Hb concentration ≥6,2 mmol/l (graad 2D). | NfN | 2015 | 2D |
| 15.14 | Anemia in patients with stage 3-5 chronic kidney disease: Erythropoiesis-stimulating agents should be prescribed in conjunction with a specialist with experience in prescribing these agents (grade D, opinion). | CMAJ | 2008 | D |
| 15.15 | At initiation of ESA therapy, a balance should be made of potential benefits (avoiding blood transfusion and anemia related symptoms) and risks (eg. stroke, hypertension) (graad 1B). | NfN | 2015 | 1B |
| 15.16 | ESA has to be used with great caution (or preferably avoided) in patients with active malignancy (by all means in patiens with a chance of being cured) (graad 1B), with a history of stroke (graad 1B) and with a history of malignancy (graad 2C). | NfN | 2015 | 1B  1B  2C |
| 15.17 | In patients with “ESA hyporesponsiveness” it is suggested to not more than double the dose relative to an adequate initiation dose based on weight or a preceding stable maintenance dose. | NfN | 2015 | 2D |
| 15.18 | There is “ESA hyporesponsiveness” if after treatment with a stable dose of ESA, the dose has to be more than doubled to maintain a stable Hb concentration (Not graded). | NfN | 2015 | None |
| 15.19 | In patients with stage 3-5 chronic kidney disease: For patients not receiving erythropoiesis-stimulating agents and who have a hemoglobin level < 110 g/L, iron should be administered to maintain a level of ferritin > 100 ng/mL and transferrin saturation > 20% (grade D). | CMAJ | 2008 | D |
| 15.20 | In patients with stage 3-5 chronic kidney disease: For patients receiving erythropoiesis-stimulating agents, iron should be administered to maintain a level of ferritin > 100 ng/mL and transferrin saturation > 20% (grade D). | CMAJ | 2008 | D |
| 15.21 | In patients with stage 3-5 chronic kidney disease: The oral form of iron is the preferred first-line therapy for patients with chronic kidney disease (grade D, opinion). | CMAJ | 2008 | D |
| 15.22 | In patients with stage 3-5 chronic kidney disease: Patients who do not achieve serum ferritin or transferrin saturation targets or both while taking the oral form of iron or who do not tolerate the oral form should receive the intravenous form of iron (grade D, opinion). | CMAJ | 2008 | D |
| 15.23 | When iron supplements are prescribed, benefits (avoidance of blood transfusion, treatment with ESA and decrease of anemia-related symptoms) have to be balanced against the risks (such as gastro-intestinal adverse effects, anafylactic reactions and unknown longterm effects.(Not graded). | NfN | 2015 | None |
| 15.24 | A trial of oral or IV iron is suggested to patients with anemia without iron supplementation and with/without ESA if an increase in Hb concentration is preferred without initiating or increasing ESA dosage, transferrinesaturation ≤25% and ferritine concentration<200 mg/l is (graad 2C). | NfN | 2015 | 2C |

| 1. **CKD Treatment: mineral metabolism abnormalities** | | | | |
| --- | --- | --- | --- | --- |
|  | **Recommended indicator** | **Source** | **Date** | **Evidence  grading** |
| 16.1 | We recommend measuring serum levels of calcium, phosphate, PTH, and alkaline phosphatase activity at least once in adults with GFR < 45 ml/min/1.73 m2 (GFR categories G3b-G5) in order to determine baseline values and inform prediction equations if used. | KDIGO  SIGN 103 | 2012  2008 | 1C  GPP |
| 16.2 | Serum calcium, phosphate and parathyroid hormone levels should be measured for adults with stage 4 and 5 chronic kidney disease, and for adults with stage 3 chronic kidney disease and a progressive decline in renal function (grade D, opinion). | CMAJ | 2008 | D |
| 16.3 | Measure serum calcium, phosphate and PTH concentrations in people with a GFR of less than 30 ml/min/1.73 m2 (GFR category G4 or G5). Determine the subsequent frequency of testing by the measured values and the clinical circumstances. Where doubt exists, seek specialist opinion. | DM  NICE CG182 | 2012  2014 (update 2015) | 1C  None |
| 16.4 | Do not routinely measure calcium, phosphate, parathyroid hormone (PTH) and vitamin D levels in people with a GFR of 30 ml/min/1.73 m^2^ or more (GFR category G1, G2 or G3) | NICE CG182 | 2014 (2015 update) | None |
| 16.5 | Serum calcium levels should be maintained within the normal range (grade D). | CMAJ | 2008 | D |
| 16.6 | In people with GFR o45 ml/min/1.73m2 (GFR categories G3b-G5), we suggest maintaining serum phosphate concentrations in the normal range according to local laboratory reference values. (2C) | NfN  KDIGO | 2015  2012 | 2C  2C |
| 16.7 | Serum phosphate levels should be maintained within the normal range (grade C). | CMAJ | 2008 | C |
| 16.8 | In people with GFR o45 ml/min/1.73m2 (GFR categories G3b-G5) the optimal PTH level is not known. We suggest that people with levels of intact PTH above the upper normal limit of the assay are first evaluated for hyperphosphatemia, hypocalcemia, and vitamin D deficiency. (2C) | NfN  KDIGO | 2015  2012 | 2C  2C |
| 16.9 | Intact parathyroid hormone levels may be elevated above normal values; the target level of serum intact parathyroid hormone is unknown (grade D, opinion). | CMAJ | 2008 | D |
| 16.10 | We suggest not to perform bone mineral density testing routinely in those with eGFR < 45 ml/min/1.73 m2 (GFR categories G3b-G5), as information may be misleading or unhelpful. | KDIGO | 2012 | 2B |
| 16.11 | **7.9.2 Treatment mineral metabolism abnormalities** |  |  |  |
| 16.12 | Dietary phosphate restriction should be used continuously to treat hyperphosphatemia (grade D). | CMAJ | 2008 | D |
| 16.13 | Therapy with calcium-containing phosphate binders (calcium carbonate or calcium acetate) should be initiated if dietary restriction fails to control hyperphosphatemia and if hypercalcemia is not present (grade D). | CMAJ | 2008 | D |
| 16.14 | If hypercalcemia develops, the dose of calcium-containing phosphate binders or vitamin D analogues should be reduced (grade D, opinion). | CMAJ | 2008 | D |
| 16.15 | Hypocalcemia should be corrected if the patient has symptoms or if it is associated with increasing parathyroid hormone levels (grade D, opinion). | CMAJ | 2008 | D |
| 16.16 | Consider prescribing vitamin-D analogues if serum levels of intact parathyroid hormone are > 53 pmol/L. Therapy should be discontinued if hypercalcemia or hyperphosphatemia develops or if parathyroid hormone levels are < 10.6 pmol/L. Vitamin-D analogues should be used in conjunction with a specialist with experience in prescribing these agents (grade D, opinion). | CMAJ | 2008 | D |
| 16.17 | There is insufficient evidence to recommend use of phosphate binders that do not contain calcium, novel vitamin-D analogues or calcimimetics (grade D, opinion) | CMAJ | 2008 | D |
| 16.18 | We suggest not to routinely prescribe vitamin D supplements or vitamin D analogs, in the absence of suspected or documented deficiency, to suppress elevated PTH concentrations in people with CKD not on dialysis. (2B) | NfN  KDIGO  NICE CG182 | 2015  2012  2014 (2015 update) | 2B  2B  None |
| 16.19 | Offer colecalciferol or ergocalciferol to treat vitamin D deficiency in people with CKD and vitamin D deficiency | NICE CG182 | 2014 (2015 update) | None |
| 16.20 | If vitamin D deficiency has been corrected and symptoms of CKD–mineral and bone disorders persist, offer alfacalcidol (1‑alpha‑hydroxycholecalciferol) or calcitriol (1‑25‑dihydroxycholecalciferol) to people with a GFR of less than 30 ml/min/1.73 m^2^ (GFR category G4 or G5) | NICE CG182 | 2014 (2015 update) | None |
| 16.21 | Monitor serum calcium and phosphate concentrations in people receiving alfacalcidol or calcitriol supplements | NICE CG182 | 2014 (2015 update) | None |
| 16.22 | Consider offering vitamin D if there is a 25-OH-vitamine D deficiency or an increase of PTH (Grade 2B): ∙   - Colecalciferol or ergocalciferol in patients with CKD stage 1, 2 and 3 (GFR ≥30 ml/min/1,73 m2 ) and a vitamin D deficiency; - Alfacalcidol or calcitrol in patients with CKD stage 4 and 5 (GFR <30 ml/min/1,73 m2 ) with secundary hyperparathyroidism despite treatment with colecalciferol of ergocalciferol. - Monitor serum calcium and phosphate in these cases. | DM | 2012 | 2B |
| 16.23 | General guidelines for the management of osteoporosis should be applied to patients with chronic kidney disease | SIGN 103 | 2008 | GPP |
| 16.24 | We suggest not to prescribe bisphosphonate treatment in people with GFR 30 ml/min/1.73 m2 (GFR categories G4-G5) without a strong clinical rationale. | KDIGO | 2012 | 2B |
| 16.25 | Offer bisphosphonates if indicated for the prevention and treatment of osteoporosis in people with a GFR of 30 ml/min/1.73 m^2^ or more (GFR category G1, G2 or G3) | NICE CG182 | 2014 (2015 update) | None |

| 1. **Alternations in medication and safety of the patient** | | | | |
| --- | --- | --- | --- | --- |
|  | **Recommended indicator** | **Source** | **Date** | **Evidence  grading** |
| 17.1 | In the context of drug management and patient safety, we recommend the patient to inform every health care provider of his/her CKD. | EBM | 2017 | GPP |
| 17.2 | Physicians should be aware of drugs with active metabolites that can exaggerate pharmacologic effects in patients with renal impairment. | AAFP | 2007 | C |
| 17.3 | We recommend that prescribers should take GFR into account when drug dosing. | KDIGO  SIGN 103  NfN  EBM | 2012 2008  2015  2017 | 1A  GPP  None  1A |
| 17.4 | Dosages of drugs cleared renally should be adjusted based on the patient's renal function (calculated as creatinine clearance or glomerular filtration rate); initial dosages should be determined using published guidelines and adjusted based on patient response; serum drug concentrations should be used to monitor effectiveness and toxicity when appropriate. | AAFP | 2007 | C |
| 17.5 | Where precision is required for dosing (due to narrow therapeutic or toxic range) and/or estimates may be unreliable (e.g., due to low muscle mass), we recommend methods based upon cystatin C or direct measurement of GFR. (1C) | KDIGO | 2012 | 1C |
| 17.6 | In patients with heart failure, prescribe diuretics at the lowest effective dose, slowly alter doses and only use diuretics in case of symptoms of fluid retention (Grade 1C). | DM | 2012 | 1C |
| 17.7 | In patiënts with heart failure and CKD, avoid the use of spironolactone | DM | 2012 | 2C |
| 17.8 | In patiënts with heart failure and CKD, avoid the use of digoxin due to the higher risk of intoxication. When digoxin use is required, reduced doses will be administered. | DM | 2012 | 2C |
| 17.9 | Potassium and renal function should be checked after commencing and changing the dose of angiotensin converting enzyme inhibitors and/or angiotensin receptor blockers. | SIGN 103 | 2008 | GPP |
| 17.10 | In people with CKD, measure serum potassium concentrations and estimate the GFR before starting renin–angiotensin system antagonists. Repeat these measurements between 1 and 2 weeks after starting renin–angiotensin system antagonists and after each dose increase. | NICE CG182  MSN | 2014 (2015 update)  2011 | None  B |
| 17.11 | To improve concordance, inform people who are prescribed renin–angiotensin system antagonists about the importance of:   - achieving the optimal tolerated dose of renin–angiotensin system antagonists and - monitoring eGFR and serum potassium in achieving this safely | NICE CG182 | 2014 (2015 update) | None |
| 17.12 | Observe renal function when using an ACE inhibitor. | DM | 2012 | 1C |
| 17.13 | If there is a sustained rise in creatinine levels above 30% (or estimated glomerular filtration rate reduces >25%) from the baseline or serum potassium is >5.6 mmol/l during the first two months after commencement of ACEi/ARB therapy, reduce or discontinue the ACEi/ARB after excluding other precipitating factors and refer to a nephrologist/physician. (Grade B) | MSN | 2011 | B |
| 17.14 | If there is a decrease in eGFR or increase in serum creatinine after starting or increasing the dose of renin–angiotensin system antagonists, but <25% (eGFR) or <30% (serum creatinine) of baseline, repeat the test in 1–2 weeks. Do not modify the renin–angiotensin system antagonist dose if the change in eGFR is <25% or the change in serum creatinine is <30% | NICE CG182 | 2014 (2015 update) | None |
| 17.15 | We recommend temporary discontinuation of potentially nephrotoxic and renally excreted drugs in people with a  GFR <60 ml/min/1.73 m2 (GFR categories G3a-G5) who have serious intercurrent illness that increases the risk of  AKI. These agents include, but are not limited to: RAAS blockers (including ACE-Is, ARBs, aldosterone inhibitors,  direct renin inhibitors), diuretics, NSAIDs, metformin, lithium, and digoxin. (1C) | EBM  NfN  KDIGO | 2017  2015  2012 | 1C  1C  1C |
| 17.16 | We recommend that adults with CKD seek medical or pharmacist advice before using over-the-counter medicines or  nutritional protein supplements. (1B) | EBM  KDIGO | 2017  2012 | 1B  1B |
| 17.17 | We recommend not using herbal remedies in people with CKD. (1B) | KDIGO  EBM | 2012  2017 | 1B  1B |
| 17.18 | Patients with chronic kidney disease who express an intent to use alternative or complementary therapies should be made aware of the lack of evidence to support their use and be warned of the possible nephrotoxic effects of some Chinese and Ayurvedic medicines. | SIGN 103 | 2008 | GPP |
| 17.19 | In patients with chronic kidney disease, over-the-counter and herbal medicine use should be assessed to ensure that medications are indicated; medications with toxic metabolites should be avoided, the least nephrotoxic agents should be used, and alternative medications should be used if potential drug interactions exist. | AAFP | 2007 | C |
| 17.20 | Monitor GFR at least annually in people prescribed drugs known to be nephrotoxic, such as calcineurin inhibitors (for example, cyclosporin or tacrolimus), lithium and non-steroidal anti-inflammatory drugs (NSAIDs). | NICE CG182 | 2014 (2015 update) | None |
| 17.21 | We recommend that all people taking potentially nephrotoxic agents such as lithium and calcineurin inhibitors should have their GFR, electrolytes and drug levels regularly monitored. (1A) | EBM  KDIGO | 2017  2012 | 1A  1A |
| 17.22 | People with CKD should not be denied therapies for other conditions such as cancer but there should be appropriate dose adjustment of cytotoxic drugs according to knowledge of GFR. (Not Graded) | KDIGO | 2012 | None |
| 17.23 | In people with CKD the chronic use of NSAIDs may be associated with progression and acute use is associated with a reversible decrease in GFR. Exercise caution when treating people with CKD with NSAIDs over prolonged periods of time. Monitor the effects on GFR, particularly in people with a low baseline GFR and/or in the presence of other risks for progression. | NICE CG182 | 2014 (2015 update) | None |
| 17.24 | Acetaminophen (paracetamol) is the analgesic of choice for short-term treatment of mild to moderate pain in patients with stage 3 to 5 CKD. | AAFP | 2011 | C |

| 1. **Safety: Imaging and contrast agents** | | | | |
| --- | --- | --- | --- | --- |
|  | **Recommended indicator** | **Source** | **Date** | **Evidence  grading** |
| 18.1 | Determine the eGFR before each examination with contrast agent, if no recent (last 12 months) value is known (Grade 1B) | DM | 2012 | 1B |
| 18.2 | Balance the risk of acute impairment in kidney function due to contrast agent use against the diagnostic value and therapeutic implications of the investigation. (Not Graded) | KDIGO | 2012 | None |
| 18.3 | Inform the performer of any examination with a contrast agent of the patients renal function and discuss the preventive measures that have to be taken (Grade 1B). | DM | 2012 | 1B |
| 18.4 | If possible, spread examinations in time (with a minimum of two weeks) and always check the eGFR before a re-examination (Grade 1B) | DM | 2012 | 1B |
| 18.5 | We recommend that all people with GFR < 60 ml/min/1.73 m2 (GFR categories G3a-G5) undergoing elective  investigation involving the intravascular administration of iodinated radiocontrast media should be managed  according to the KDIGO Clinical Practice Guideline for AKI including:   - Avoidance of high osmolar agents (1B); - Use of lowest possible radiocontrast dose (Not Graded); - Withdrawal of potentially nephrotoxic agents before and after the procedure (1C); - Adequate hydration with saline before, during, and after the procedure (1A); - Measurement of GFR 48–96 hours after the procedure (1C). | NfN  KDIGO | 2015  2012 | 1B None  1C  1A  1C  (idem voor KDIGO) |
| 18.6 | We recommend not using gadolinium-containing contrast media in people with GFR < 15 ml/min/1.73 m2  (GFR category G5) unless there is no alternative appropriate test. (1B) | NfN  KDIGO | 2015  2012 | 1B  1B |
| 18.7 | **Gadolinium** should be avoided in patients with a glomerular filtration rate less than 30 mL per minute per 1.73 m2, or with acute kidney injury caused by hepatorenal syndrome or in the perioperative liver transplantation period. | AAFP | 2012 | B |
| 18.8 | We suggest that people with a GFR o30 ml/min/1.73 m2 (GFR categories G4-G5) who require gadoliniumcontaining  contrast media are preferentially offered a macrocyclic chelate preparation. (2B) | NfN  KDIGO | 2015  2012 | 2B  2B |
| 18.9 | We recommend not to use **oral phosphate-containing bowel preparations** in people with a **GFR < 60** ml/min/1.73 m2 (GFR categories G3a-G5) or in those known to be at risk of phosphate nephropathy. (1A) | KDIGO | 2012 | 1A |

| 1. **Referral to specialist** | | | | |
| --- | --- | --- | --- | --- |
|  | **Recommended indicator** | **Source** | **Date** | **Evidence  grading** |
| 19.1 | The nephrologist takes care of the diagnostic work-up of high risk patients, follow-up or supervision of stage 4 and ensures tackling of and follow-up of terminal kidney disease (GPP). | EBM | 2017 | GPP |
| **We recommend referral for patients with CKD to a nephrologist or a health care professional specialized in CKD in the following cases:** | | | | |
| 19.2 | Acute kidney injury or abrupt sustained fall in GFR | NfN, KDIGO | 2015  2012 | 1B,1B |
| 19.3 | GFR <30 ml/min/1,73 m2 (GFR categorie G4 or G5), | NfN, KDIGO, AAFP, AAFP, NICE CG 182, MSN  DM | 2015,  2012,  2004 (I),  2011, 2014 (2015 update),2011  2012 | 1B, 1B, C, C, None, C  2B |
| 19.4 | Patients <75 jaar with an eGFR between 30 and 45 ml/min./1,73 m² and an ACR of 20-200mg/g for males and 30-300 mg/g for females(GRADE 2B); | EBM  DM | 2017  2012 | 2B  2B |
| 19.5 | Patients with an eGFR >45 ml/min./1,73 m² and an ACR >200 mg/g for males or 300 mg/g for females and/or a PCR >1 000 mg/g (GRADE 2B); | EBM  DM | 2017  2012 | 2B  2B |
| 19.6 | A consistent finding of significant albuminuria (ACR ⋝ 300 mg/g [⋝30 mg/mmol] or AER ⋝ 300 mg/ 24 hours, approximately equivalent to PCR ⋝ 500 mg/g [⋝50 mg/mmol] or PER ⋝500 mg/24 hours | NfN, KDIGO, EBM | 2015,2012,2017 | 1B,1B, 1B |
| 19.7 | Heavy proteinuria (urine protein ≥1 g/day or urine protein: creatinine ratio (uPCR) ≥0.1 g/mmol) unless known to be due to diabetes and optimally treated | MSN | 2011 | C |
| 19.8 | Haematuria with proteinuria (urine protein ≥0.5 g/day or uPCR ≥0.05 g/mmol) | MSN | 2011 | C |
| 19.9 | ACR 70 mg/mmol or more, unless known to be caused by diabetes and already appropriately treated | NICE CG182 | 2014 (update 2015) | None |
| 19.10 | ACR 30 mg/mmol or more (ACR category A3), together with haematuria | NICE CG182 | 2014 (update 2015) | None |
| 19.11 | Progression of CKD: confirmed decline in GFR category accompanied by a 25% or greater drop in eGFR from baseline or a sustained decline in eGFR of more than 5 ml/min/1.73 m2 /year. | NfN, KDIGO | 2015,2012 | 1B,1B |
| 19.12 | Patients with progressive CKD, being an eGFR between 30 en 45 ml/min./1,73 m² and a decline of eGFR >10 ml/min in five years time or > 5 ml/min in two years time (GRADE 2B); | EBM  DM | 2017  2012 | 2B  2B |
| 19.13 | Rapidly declining renal function (loss of glomerular filtration rate/GFR >5 ml/min/1.73m2 in one year or >10 ml/min/1.73m2 Within five years) | MSN | 2011 | C |
| 19.14 | Sustained decrease in GFR of 25% or more, and a change in GFR category or sustained decrease in GFR of 15 ml/min/1.73 m^2^ or more within 12 months | NICE (CG182) | 2014 (update 2015) | None |
| 19.15 | Dysmorphic erytrocytes in urine sedimentation (>20 pgv); | NfN, KDIGO | 2015,2012 | 1B,1B |
| 19.16 | Patiënten with hematuria of unknown origin (GRADE 1B); | EBM | 2017 | 1B |
| 19.17 | CKD combined with hypertension, which insufficiëntly responds to medical treatment with 4 or more antihypertensive drugs; | NfN, KDIGO, EBM, MSN, NICE CG182 | 2015,2012, 2017, 2011, 2014 (update 2015) | 1B,1B, 1B, C, None |
| 19.18 | Persisting serum potassium abnormalities; | NfN, KDIGO, EBM | 2015,2012,2017 | 1B,1B, 1B |
| 19.19 | Recurrent or extensive nefrolithiasis; | NfN, KDIGO, EBM | 2015,2012,2017 | 1B,1B, 1B |
| 19.20 | Hereditary kidney disease; | NfN, KDIGO, EBM, MSN, NICE CG182 | 2012,2015, 2017,2011,2014 (update 2015) | 1B,1B, 1B, C, None |
| 19.21 | When a.renalis stenosis is suspected or established; | MSN, EBM | 2011,2017 | C, GPP |
| 19.22 | For treatment of complications of kidney failure: anemia, electrolyte imbalance, vitamin D, calcium- and phosphate disturbances, uremic complaints (pruritus,…) (GPP); | EBM | 2017 | GPP |
| 19.23 | Suspected glomerular disease | MSN, NICE CG182 | 2011, 2014 (update 2015) | C, None |
| 19.24 | Pregnant or when pregnancy is planned | MSN | 2011 | C |
| 19.25 | Unclear cause of CKD. | MSN | 2011 | C |
| **Referral to other specialists** | | | | |
| 19.26 | Refer all referable patients to a nephrologist. People with CKD and renal outflow obstruction should normally be referred to urological services, unless urgent medical intervention is required – for example, for the treatment of hyperkalaemia, severe uraemia, acidosis or fluid overload (GPP). | EBM, NICE CG182  DM | 2017 2014 (update 2015)  2012 | GPP  None  Consensus |
| 19.27 | Seek specialist advice about options for treating people at high risk of CVD such as those with CKD, type 1 diabetes, type 2 diabetes or genetic dyslipidaemias, and those with CVD, who are intolerant to 3 different statins. Advice can be sought for example, by telephone, virtual clinic or referral | NICE CG181 | 2014 (2016 update) | None |
| 19.28 | In case the patient with progressive chronic kidney disease or the medical team choose not to pursue renal replacement therapies, if he/she wishes to do so, he/she should be ensured to have access to multidisciplinary support through a program with attention to medical, psychological and socio-cultural aspects.(Not graded) | NfN  CMAJ | 2015  2008 | None  D |
| 19.29 | Conservative management should be an option in people who choose not to pursue RRT and this should be supported by a comprehensive management program. (Not Graded) | KDIGO | 2012 | None |
| 19.30 | Refer high risk patiënts for inclusion in the care program. These are patients with:   - a chronic eGFR <30 ml/min./1,73 m² (eGFR categories G4-G5) (GRADE 1B); - an eGFR between 30-45 ml/min./1,73 m² and ACR >200 mg/g for males or 300 mg/g for females, and/or proteïnuria >1000 mg/24h or a protein-creatininratio (PCR) >1 000 mg/g (GRADE 2B). | EBM | 2017 | 1B  2B |

| 1. **Renal replacement therapy** | | | | |
| --- | --- | --- | --- | --- |
|  | **Recommended indicator** | **Source** | **Date** | **Evidence  grading** |
| 20.1 | Components of care prior to initiation   - If feasible, patients with an estimated GFR < 30 mL/min/m2 should receive care in a multidisciplinary setting that includes physicians, nurses, dietiticians and social workers (grade C). | CMAJ | 2008 |  |
| 20.2 | Components of care prior to initiation   - A predialysis education program should include lifestyle modification, medication management, modality selection and vascular access as well as options for renal transplantation (grade D, opinion). | CMAJ | 2008 |  |
| 20.3 | No evidence currently exists upon which to recommend a GFR at which renal replacement therapy should be initiated in the absence of complications of chronic kidney disease (grade D, opinion). | CMAJ | 2008 |  |
| 20.4 | We recommend timely referral for planning renal replacement therapy (RRT) in people with progressive CKD in whom the risk of kidney failure within 1 year is 10–20% or higherw , as determined by validated risk prediction tools. (1B) | EBM  KDIGO | 2017  2012 |  |
| 20.5 | We recommend timely referral of people with progressive CKD, for whom initiation of renal replacement therapy (RRT) is expected in one to two years, to prepare them multidisciplinarily for RRT through a structured care program. (Grade 1B) | NfN | 2015 |  |
| 20.6 | Patients with an estimated GFR < 20 mL/min/m2 may require initiation of renal replacement therapy if any of the following are present:   - Symptoms of uremia (after excluding other causes), - Refractory metabolic complications (hyperkalemia, acidosis), - Volume overload (manifesting as resistant edema or hypertension) - A decline in nutritional status (as measured by serum albumin, lean body mass or Subjective Global Assessment) that is refractory to dietary intervention (grade D, opinion). | CMAJ | 2008 | Grade D, opinion |
| 20.7 | We suggest that dialysis be initiated when one or more of the following are present:   - symptoms or signs attributable to kidney failure (serositis, acidbase or electrolyte abnormalities, pruritus); - inability to control volume status or blood pressure; - a progressive deterioration in nutritional status refractory to dietary intervention; - cognitive impairment. This often but not invariably occurs in the GFR range between 5 and 10 ml/min/1.73 m2. (2B) | EBM  KDIGO | 2017  2012 |  |
| 20.8 | Living donor preemptive renal transplantation in adults should be considered when the GFR is o20 ml/min/1.73 m2 , and there is evidence of progressive and irreversible CKD over the preceding 6-12 months.(Not Graded) | EBM  KDIGO  CMAJ | 2017  2012  2008 | Not graded  ?  ? |
| 20.9 | Concerning patients who underwent kidney transplantation:  frequent follow-up during the first year posttransplantation is necessary and is best planned in the transplant centre during the first three months. Afterwards, the treating nephrologist can continue nephrologic follow-up.(GPP) | EBM | 2017 | GPP |
| 20.10 | Concerning patients who underwent kidney transplantation: After 1 year, frequent nephrological follow-up remains necessary, ideally every 2 to 3 months including laboratory check-ups. This is performed by the treating nephrologist. Minimally once a year there is a follow-up contact in the specialized nephrological transplant centre. Transplant pathology is complex, specific and demands special care. Dosing of immunosuppressants and knowledge of interactions demand some experience. | EBM | 2017 |  |
| 20.11 | Concerning patients who underwent kidney transplantation:  Good appointments and good communication between the specialized transplant centre, the treating nephrologist and the general practitioner are essential. Within the context of a transplanted patient, the general practitioner should smoothly tackle problems specific for primary care, including overall prevention. During follow-up, attention to therapy compliance is crucial.(GPP) | EBM | 2017 | GPP |
| 20.12 | Concerning patients who underwent kidney transplantation: all involved doctors (transplant centre, treating nephrologist and general practitioner) should have attention for (GPP):   - timely detection and follow-up of cardiovascular disease, hypertension in particular, - detection of diabetes mellitus, - lifestyle advice such as to quit smoking, - vaccination policy, - timely treating infection, - malignancies and medical interactions of immunosuppressants | EBM | 2017 | GPP |
| 20.13 | Conservative management should be an option in people who choose not to pursue RRT and this should be supported by a comprehensive management program. (Not Graded) | EBM  NfN  KDIGO | 2017  2015  2012 | 1C  None |
| 20.14 | Patients with an estimated GFR < 20 mL/min/m2 may require initiation of renal replacement therapy if any of the following are present:   - Symptoms of uremia (after excluding other causes), - Refractory metabolic complications (hyperkalemia, acidosis), - Volume overload (manifesting as resistant edema or hypertension) - A decline in nutritional status (as measured by serum albumin, lean body mass or Subjective Global Assessment) that is refractory to dietary intervention (grade D, opinion). | CMAJ | 2008 |  |
| 20.15 | We suggest that dialysis be initiated when one or more of the following are present:   - symptoms or signs attributable to kidney failure (serositis, acidbase or electrolyte abnormalities, pruritus); - inability to control volume status or blood pressure; - a progressive deterioration in nutritional status refractory to dietary intervention; - cognitive impairment. This often but not invariably occurs in the GFR range between 5 and 10 ml/min/1.73 m2. (2B) | EBM  KDIGO | 2017  2012 |  |
| 20.16 | Living donor preemptive renal transplantation in adults should be considered when the GFR is o20 ml/min/1.73 m2 , and there is evidence of progressive and irreversible CKD over the preceding 6-12 months.(Not Graded) | EBM  KDIGO  CMAJ | 2017  2012  2008 |  |
| 20.17 | Concerning patients who underwent kidney transplantation:  frequent follow-up during the first year posttransplantation is necessary and is best planned in the transplant centre during the first three months. Afterwards, the treating nephrologist can continue nephrologic follow-up.(GPP) | EBM | 2017 |  |
| 20.18 | Concerning patients who underwent kidney transplantation: After 1 year, frequent nephrological follow-up remains necessary, ideally every 2 to 3 months including laboratory check-ups. This is performed by the treating nephrologist. Minimally once a year there is a follow-up contact in the specialized nephrological transplant centre. Transplant pathology is complex, specific and demands special care. Dosing of immunosuppressants and knowledge of interactions demand some experience. | EBM | 2017 |  |
| 21.19 | Concerning patients who underwent kidney transplantation:  Good appointments and good communication between the specialized transplant centre, the treating nephrologist and the general practitioner are essential. Within the context of a transplanted patient, the general practitioner should smoothly tackle problems specific for primary care, including overall prevention. During follow-up, attention to therapy compliance is crucial.(GPP) | EBM | 2017 |  |
| 20.20 | Concerning patients who underwent kidney transplantation: all involved doctors (transplant centre, treating nephrologist and general practitioner) should have attention for (GPP):   - timely detection and follow-up of cardiovascular disease, hypertension in particular, - detection of diabetes mellitus, - lifestyle advice such as to quit smoking, - vaccination policy, - timely treating infection, - malignancies and medical interactions of immunosuppressants | EBM | 2017 | GPP |
| 20.21 | Conservative management should be an option in people who choose not to pursue RRT and this should be supported by a comprehensive management program. (Not Graded) | EBM  NfN  KDIGO | 2017  2015  2012 | 1C  None |

| 1. **Role of the GP and care program** | | | | |
| --- | --- | --- | --- | --- |
|  | **Recommended indicator** | **Source** | **Date** | **Evidence  grading** |
| 21.1 | Among the general practitioner’s tasks is screening and detecting chronic kidney disease, ensuring follow-up of patients with low or intermediary risk at terminal kidney disease, handling the follow-up list, giving and supporting lifestyle advice and tracking down critical moments. | EBM  DM | 2017  2012 | None  Consensus |
| 21.2 | We suggest that people with progressive CKD should be managed in a multidisciplinary care setting. (2B) | KDIGO EBM | 2012  2017 | 2B  2B |
| 21.3 | Coordinated end-of-life care should be available to people and families through either primary care or specialist care as local circumstances dictate. (Not Graded) | EBM  KDIGO  CMAJ | 2017  2012  2008 | GPP  None  D |
| 21.4 | We suggest that people with progressive CKD should be managed in a multidisciplinary care setting. (2B) The multidisciplinary team should include or have access to dietary counseling, education and counseling about different RRT modalities, transplant options, vascular access surgery, and ethical, psychological, and social care. (Not Graded) | EBM  KDIGO | 2017  2012 | GPP  None |
| 21.5 | The comprehensive conservative management program should include protocols for symptom and pain management, psychological care, spiritual care, and culturally sensitive care for the dying patient and their family (whether at home, in a hospice or a hospital setting), followed by the provision of culturally appropriate bereavement support. (Not Graded) | EBM  KDIGO  CMAJ | 2017  2012  2008 | GPP  None  D |
| 21.6 | Once a referral has been made and a plan jointly agreed (between the person with CKD or their carer and the healthcare professional), it may be possible for routine follow-up to take place at the patient's GP surgery rather than in a specialist clinic. If this is the case, criteria for future referral or re-referral should be specified. | EBM  NICE CG182 | 2017  2014(2015 update) | GPP None |
| 21.7 | Performant IT (Electronic medical record**,** electronic patient file, MHealth) can offer a strong support to identify patients with a high or intermediary risk (as defined in question 2) with help of built-in calculators and algorythms in the EMR (GPP). | EBM | 2017 | GPP |
