# Supplementary material for: Developing quality indicators for Chronic Kidney Disease in primary care, extractable from the Electronic Medical Record. A Rand-modified Delphi method
Source: BMC Nephrol. 2020 May 5;21:161. doi: 10.1186/s12882-020-01788-8 (PMC7201612; doi:10.1186/s12882-020-01788-8)
Supplement: Supplementary file 2 — Additional file 2. Addendum 2: Questionnaire for the professionals [file 12882_2020_1788_MOESM2_ESM.docx]

**Addendum 2: Letter and questionnaire professional panelmembers**

Dear Sir / Madam

First of all, we would like to thank you for your willingness to participate in our panel of experts. This study, in which the panel plays an important role, is being conducted at the Academic Centre for General Practice in Leuven. The main goal of our study is to develop a selection of quality indicators for chronic kidney disease (CKD). A specific requirement for the selection of indicators involves their straightforward and automatic extractability from the electronic medical record (EMR). The final objective of this study is to provide general practitioners with automatic feedback concerning their treatment of patients with CKD. In this way, we strive to ensue an improvement of the quality of care for patients with chronic kidney disease in primary care.

You are invited to participate in the selection of quality indicators for CKD because of your knowledge and expertise concerning the subject. As it was already explained in the invitation letter that you received, we use the Rand modified Delphi method to develop a selection of quality indicators. This consensus type method encompasses three stages. In the first stage, we ask you to score a list of recommendations according to their importance, usefulness and relevance in primary care. The purpose of this stage is to draw up a short and powerful set of recommendations, which eventually will be translated to a core set of quality indicators after the whole process has been undertaken. This document includes the questionnaire with comprehensive instructions. It takes approximately 30-40 minutes of your time to complete the questionnaire. We kindly ask you to send us back the completed questionnaire before 12th March 2018.

In the second phase, the results of the questionnaires will be analysed. A face to face discussion with all the panel members will take place to reach a consensus about the acceptance, exclusion or the reformulation of the potential indicators. This meeting, of which you will be informed about the date in the future, will take approximately two hours of your time.

In the third and last phase, the final written list of indicators will be presented to all panel members in order to achieve a definitive permission for publication. This phase will take no longer than fifteen minutes of your time. The exact date of this final step will also be announced in the future.

If you have any questions or remarks, please do not hesitate to contact us ([steve.vandenbulck@-kuleuven.be](mailto:steve.vandenbulck@-kuleuven.be)).

Yours faithfully,

The project group existing of:

Steve Van den Bulck, GP, PhD student

Professor Patrik Vankrunkelsven, KU Leuven, promotor

Professor Rosella Hermens, Radboud University, copromotor

Professor Geert Goderis, KU Leuven, copromotor

**Indicators for chronic kidney disease**

**From recommendation to indicator**

For the selection of recommendations in this document, we based our search on national and international guidelines concerning the subject. We started to draw up a list of all the recommendations of all the guidelines we consulted. However, the selection of a core set of recommendations based on such an extensive list, would be an infeasible task. Therefore, we decided to select only those recommendations that were useful in a primary care setting and that were automatically extractable from the electronic medical record. Those are namely two important conditions in the frame of our study. In addition, we selected the recommendations that met the "SMART" principles, i.e. **S**pecific, **M**easurable, **A**chievable, **R**elevant and **T**ime bound. Nevertheless, the amount of recommendations was still too extensive, so we eventually decided to reserve the recommendations based on guidelines not older than 3 year. The result is a basic set of recommendations as enclosed in this document.

**Instructions for completing the questionnaire**

In this questionnaire, you will find a list of 75 recommendations, subdivided into the following categories: definition and classification, screening, diagnosis and etiology, management: follow-up, vaccination, treatment: patient education and information, lifestyle and diet, diabetes and glycemic control, dyslipidemia, hypertension, cardiovascular disease, anemia, mineral metabolism abnormalities, drugs and patient safety, imaging and contrast agents, referral to a specialist, renal replacement therapy, role of the general practitioner and care program. We kindly ask you to judge each recommendation on its importance to measure the quality of care for patients with type 2 diabetes mellitus in primary care using a 9-point Likert scale. This is a scale with numbers ranging from 1 to 9, with 1 being the lowest score (bad recommendation to measure the quality of care) and 9 being the highest score (excellent recommendation to measure the quality of care).

You can base your judgement of whether the recommendation is a good measure of quality of care on the following criteria:

- The recommendation is relevant in the care process of first line health care.

- The recommendation extends the (disease free) survival of the patient.

- The recommendation improves the quality of life of the patient.

- The recommendation improves the effectiveness of care for the patient.

- The recommendation can be translated to an indicator that is automatically extract- able from the electronic medical record.

Besides giving a score on the 9 point Likert scale, we also ask you to draw up a top rating of recommendations per category, based on their adequateness for measuring the quality of care. You will find these columns with a top 1, 3 or 5 score listing below each main category,

Finally, we ask you to indicate which criterium was decisive when scoring the recommendation: the fact that the recommendation is easily and automatically extractable from the electronic medical record (EMR), or the fact that the recommendation itself is an excellent measure for the quality of care for patients with chronic kidney disease?

You have the opportunity to formulate remarks, to adjust recommendations or to write down own recommendations which are not listed in the questionnaire. Please use the empty documents at the end of this questionnaire for this purpose.

On the next page, you will find an example of the lay-out of the questionnaire.

**Questionnaire lay-out**

| 1. ***Category*** | | | | | | |
| --- | --- | --- | --- | --- | --- | --- |
| *To what extent are the following recommendations of importance for measuring the quality of care in the first line health care for patients with chronic kidney disease?* | | | | | | |
|  | **Recommendation** | **Source** | **Year** | **Grade of evidence** | **Your judgement** | **Decisive factor in your judgement** |
|  | The recommended medical act. | .... | .... | .... | **1 2 3 4 5 6 7 8 9**  **[----------------------------------------------------------------------------]**  Poor Excellent  *□ Not judgeable* |  Importance   EMR extractability |

The abbreviation of the consulted national or international guideline.

Here you can declare which factor was decisive in your judgement, namely the importance of the recommendation for the measurement of the quality of care, or the automatic extractability from the electronic medical record (EMR).

The year of publication or last update of the guideline.

The grade of evidence as denoted in the original guideline. For a legend of each guideline: see attachment 1.

Score of 1-9. If you deem yourself uncapable of judging the recommendation, you have the possibility to tick off the box with "not judgeable".

Here you can declare which factor was decisive in your judgement, namely the importance of the recommendation for the measurement of the quality of care, or the automatic extractability from the electronic medical record (EMR).

**Sources**

Below you will find a list of the guidelines that were consulted to draw up the list of recommendations as presented in this questionnaire. Also noted are the abbreviations of the guidelines as they are used in the questionnaire (between brackets), the year of publication or the last update of the guideline and the country of origin.

- American College of Physicians (ACP)
  - American Academy for Family Physicians (AAFP): Verenigde Staten
    - Chronic Kidney Disease: Detection and Evaluation. 2011
    - Update on the Management of Chronic Kidney Disease. 2012
    - ACP Releases Guideline on Screening, Monitoring, and Treatment of Stage 1 to 3 Chronic Kidney Disease. 2014
  - Annals of Internal Medicine
    - Screening, Monitoring, and Treatment of Stage 1 to 3 Chronic Kidney Disease: A Clinical Practice Guideline From the American College of Physicians. 2013
- Canadian Medical Association Journal (CMAJ): Guidelines for the management of chronic kidney disease. 2008; Canada
- Domus medica (DM): Chronische nierinsufficiëntie. 2012; België
- Evidence Based Medicine Practice Net (EBM): Multidisciplinaire richtlijn Chronisch nierlijden (CNI) - Aanvulling op de richtlijn ‘Chronische nierinsufficiëntie’ van Domus Medica, 2012. 2017; België
- Kidney Disease Improving Global Outcomes (KDIGO): Clinical Practice Guideline for the Evaluation and Management of Chronic Kidney Disease. 2012; Verenigde Staten
- Malaysian Society of Nephrology (MSN) Management of Chronic Kidney Disease in Adults. Clinical Practice Guidelines. 2011; Maleisië
- National Institute for Health and Care Excellence (NICE); Verenigd Koninkrijk
  - Clinical Guideline (CG) 182: Chronic kidney disease in adults: assessment and management. 2015 update
- Nederlandse Federatie voor Nefrologie (NFN): Diagnostiek en Behandeling van Patiënten met Chronische Nierschade;  2015 herziening; Nederland
- Scottish Intercollegiate Guidelines Network (SIGN) 103: Diagnosis and management of chronic kidney disease. 2008; Schotland

For more extensive information concerning the level of evidence stated by each guideline, see attachment 1.

**Informed consent**

Title of the study: The electronic medical record and the quality of care for chronic kidney
 disease

Institution: Academic Centre for General Practice, University of Leuven, Leuven (Belgium)

Project group: Steve Van den Bulck, GP, PhD student

Professor Patrik Vankrunkelsven, KU Leuven, promotor

Professor Rosella Hermens, Radboud University, copromotor

Professor Geert Goderis, KU Leuven, copromotor

I hearby declare to be informed in a comprehensible way about the nature, the method and the purpose of this study. My questions were answered to my satisfaction.

I agree voluntary to the participation in this study.

I am informed about the fact that participation in this study doesn't bring with it any additional costs and that there is no financial advantage to be gained.

I preserve the right to withdraw my consent of participation in this study, without any declaration and without it being of any influence to my personal or professional life.

My personal details cannot be obtained by a third person without my explicit permission.

If I wish to obtain more information about the study, now or in the future, I can apply to Steve Van den Bulck ([steve.vandenbulck@kuleuven.be](mailto:steve.vandenbulck@kuleuven.be)).

Read and approved,

Name of the participant: ………………………………………………………………………………

Date: ………………………………………………………………………………

Signature: ……………………………………………………………………………….

**Front page**

Name: ................................................................................................................

Function: ................................................................................................................

Hospital or GP practice: ................................................................................................................

Department: ................................................................................................................

Please send back the questionnaire **including the informed consent and front page** to:

Steve Van den Bulck

Academisch Centrum voor Huisartsgeneeskunde

Kapucijnenvoer 33 blok J - bus 7001

3000 Leuven.

**Questionnaire chronic kidney disease**

| 1. ***Definition and classification*** | | | | | |
| --- | --- | --- | --- | --- | --- |
| *To which extent are the following potential indicators important to be included in the registration for measuring the quality of care of patients with CKD?* | | | | | |
| **Recommendation** | | **Source** | **Date** | **Evidence grading** | **Rating** |
| 1 | In people with GFR < 60 ml/min/1.73 m^2^ (GFR categories G3a-G5) or markers of kidney damage, review past history and previous measurements to determine duration of kidney disease.   - If duration is >3 months, CKD is confirmed. Follow recommendations for CKD. - If duration is not >3 months or unclear, CKD is not confirmed. Patients may have CKD or acute kidney diseases (including AKI) or both and tests should be repeated accordingly. | KDIGO  AAFP  DM | 2012  2004  2012 | None  C  Consensus | **1 2 3 4 5 6 7 8 9  [---------------------------------------------------------]**  Poor Excellent *□Not judgeable*  **Rating based on:** *□ EMR extractability □ Relevance recommendation* |
| 2 | Classification of chronic kidney disease (CKD) should be based on the existing NKF-KDOQI* staging (refer to Table 3). (the KDOQI exists out of 6 GFR categories and 3 albuminuria categories) | MSN SIGN 103 NfN | 2011 2008  2015 | C GPP  None | **1 2 3 4 5 6 7 8 9  [---------------------------------------------------------]**  Poor Excellent *□Not judgeable*  **Rating based on:** *□ EMR extractability □ Relevance recommendation* |
| 3 | The suffix (p) should be added to denote the presence of proteinuria when staging CKD. | MSN | 2011 | C | **1 2 3 4 5 6 7 8 9  [---------------------------------------------------------]**  Poor Excellent *□Not judgeable*  **Rating based on:** *□ EMR extractability □ Relevance recommendation* |
| 4 | Use the person's GFR and ACR categories to indicate their **risk of adverse outcomes** (for example, CKD progression, acute kidney injury, all-cause mortality and cardiovascular events) and discuss this with them | NICE CG182 | 2014 (2015 update) | None | **1 2 3 4 5 6 7 8 9  [---------------------------------------------------------]**  Poor Excellent *□Not judgeable*  **Rating based on:** *□ EMR extractability □ Relevance recommendation* |

**Top 3 recommendations**

Which recommendations for definition and classification of chronic renal insufficiency do you find most suitable for measuring the quality of care?

| Top 3 recommendations regarding definition and classification. | | |
| --- | --- | --- |
| Position | Number of recommendation | Motivation |
| 1st position |  |  |
| 2nd position |  |  |
| 3rd position |  |  |

| 1. ***CKD Screening, diagnosis and etiology*** | | | | | |
| --- | --- | --- | --- | --- | --- |
| 5 | Do not test for **proteinuria** in adults with or without diabetes who are currently taking an **angiotensin-converting** enzyme **inhibitor** or an angiotensin II–receptor blocker. | ACP/AAFP Annals AAFP | 2013  2013 2014 | (Grade: weak recommendation, low-quality evidence) | **1 2 3 4 5 6 7 8 9  [---------------------------------------------------------]**  Poor Excellent *□Not judgeable*  **Rating based on:** *□ EMR extractability □ Relevance recommendation* |
| 6 | Confirm a positive test strip (1+ or more) with a quantitative measurement and express it as a ratio to creatinine (ACR or PCR). | NfN  KDIGO  AAFP  MSN | 2015  2012  2004 (II)  2011 | None  None  C  C | **1 2 3 4 5 6 7 8 9  [---------------------------------------------------------]**  Poor Excellent *□Not judgeable*  **Rating based on:** *□ EMR extractability □ Relevance recommendation* |
| 7 | Screening for **proteinuria** should be performed for all patients who are at high risk of kidney disease (patients with diabetes, hypertension, vascular disease, autoimmune disease, eGFR < 60 mL/min/1.73m2 or edema). | CMAJ  NICE CG 182 | 2008  2014 (2015 update) | D  None | **1 2 3 4 5 6 7 8 9  [---------------------------------------------------------]**  Poor Excellent *□Not judgeable*  **Rating based on:** *□ EMR extractability □ Relevance recommendation* |
| 8 | To detect and **identify proteinuria**, use urine ACR in preference to protein:creatinine ratio (PCR), because it has greater sensitivity than PCR for low levels of proteinuria. For quantification and **monitoring** of levels of proteinuria of ACR 70 mg/mmol or more, PCR can be used as an alternative.  ACR is the recommended method for people with diabetes. | NICE CG182  CMAJ | 2014 (2015 update) 2008 | None  B | **1 2 3 4 5 6 7 8 9  [---------------------------------------------------------]**  Poor Excellent *□Not judgeable*  **Rating based on:** *□ EMR extractability □ Relevance recommendation* |
| 9 | For the initial detection of proteinuria, if the ACR is between 3 mg/mmol and 70 mg/mmol, this should be confirmed by a subsequent early morning sample. If the initial ACR is 70 mg/mmol or more, a repeat sample need not be tested . | EBM NICE CG182  NfN KDIGO | 2017 2014 (2015 update) 2015 2012 | 1B None  None None | **1 2 3 4 5 6 7 8 9  [---------------------------------------------------------]**  Poor Excellent *□Not judgeable*  **Rating based on:** *□ EMR extractability □ Relevance recommendation* |
| 10 | Assess **GFR and albuminuria** at least **annually** in people with CKD. Assess GFR and albuminuria more often for individuals at higher risk of progression, and/or where measurement will impact therapeutic decisions. | KDIGO | 2012 | None | **1 2 3 4 5 6 7 8 9  [---------------------------------------------------------]**  Poor Excellent *□Not judgeable*  **Rating based on:** *□ EMR extractability □ Relevance recommendation* |
| 11 | During treatment, aim to keep albumin concentrations <30 mg / mmol (or <300 mg / 24 hours, or proteinuria concentrations ​​<0.5 g / 24 hours), independent of blood pressure. This can be achieved by increasing the dose renin–angiotensin system antagonists  or Angiotensin receptor Blockers, or combining these with dietary salt restriction of 3 to 5 gram and / or a (thiazide) diuretic. In this case, determine feasibility individually. | NfN | 2015 | 2D | **1 2 3 4 5 6 7 8 9  [---------------------------------------------------------]**  Poor Excellent *□Not judgeable*  **Rating based on:** *□ EMR extractability □ Relevance recommendation* |
| 12 | In patients with risk factors for developing CKD, such as hypertension or diabetes mellitus or a history of cardiovascular disease, it is desirable to measure eGFR and albuminuria once a year. | NfN MSN SIGN 103 DM | 2015 2011 2008  2012 | Niet  C GPP  2C | **1 2 3 4 5 6 7 8 9  [---------------------------------------------------------]**  Poor Excellent *□Not judgeable*  **Rating based on:** *□ EMR extractability □ Relevance recommendation* |

**Top 5 recommendations**

Which recommendations for screening, diagnosis and etiology of chronic renal insufficiency do you find most suitable for measuring the quality of care?

| Top 5 recommendations regarding screening, diagnosis and etiology. | | |
| --- | --- | --- |
| Position | Number of recommendation | Motivation |
| 1st position |  |  |
| 2nd position |  |  |
| 3rd position |  |  |
| 4th position |  |  |
| 5th position |  |  |

| 1. ***Management: follow-up*** | | | | | |
| --- | --- | --- | --- | --- | --- |
| 13 | Identify the **rate of progression of CKD:** Obtain a minimum of 3 GFR estimations over a period of not less than 90 days. | NICE CG182  DM | 2014 (2015 update)  2012 | None  Consensus | **1 2 3 4 5 6 7 8 9  [---------------------------------------------------------]**  Poor Excellent *□Not judgeable*  **Rating based on:** *□ EMR extractability □ Relevance recommendation* |
| 14 | Identify the **rate of progression of CKD** in people with a new finding of reduced GFR, repeat the GFR within 2 weeks to exclude causes of acute deterioration of GFR – for example, acute kidney injury or starting [renin–angiotensin system antagonist](http://www.nice.org.uk/guidance/cg182/chapter/recommendations#terms-used-in-this-guideline) therapy | NICE CG182 | 2014 (2015 update) | None | **1 2 3 4 5 6 7 8 9  [---------------------------------------------------------]**  Poor Excellent *□Not judgeable*  **Rating based on:** *□ EMR extractability □ Relevance recommendation* |
| 15 | Use the following table to guide the frequency of GFR monitoring for people with, or at risk of CKD:   \| **Stage** \| **GFR** \| **Test** \| **Frequency** \| \| --- \| --- \| --- \| --- \| \| 1 and 2 \| >60 \| eGFR \| Annually \| \| 3A \| 45-59 \| eGFR \| Every six months \| \| 3B \| 30-44 \| eGFR \| Every six months \| \| 4 \| 15-29 \| eGFR \| Min. Every tree months \|  - Tailor the frequency depending on the presence of other risk factors on the development of terminal renal failure and the progression of the eGFR - Let the frequency of detecting proteinuria and complications suspend upon the eGFR, the progression of the eGFR and drug therapy | DM | 2012 | Consensus | **1 2 3 4 5 6 7 8 9  [---------------------------------------------------------]**  Poor Excellent *□Not judgeable*  **Rating based on:** *□ EMR extractability □ Relevance recommendation* |
| 16 | Metabolic complications of kidney failure: Measure serum potassium, calcium, phosphate, PTH and bicarbonate levels, and Hb in patiënts with CKD with a moderate (code orange) to strongly (code red) increased risk. In case of increased PTH, also measure vitamin D, and in the case of reduced Hb also measure ferritin and transferrin saturation. The frequency of these measurements depends on the degree of kidney damage. | NfN | 2015 | none | **1 2 3 4 5 6 7 8 9  [---------------------------------------------------------]**  Poor Excellent *□Not judgeable*  **Rating based on:** *□ EMR extractability □ Relevance recommendation* |

**Top 3 recommendation**

Which recommendations for ‘managment: follow-up’ of chronic renal insufficiency do you find most suitable for measuring the quality of care?

| Top 3 recommendations regarding managment: follow-up | | |
| --- | --- | --- |
| Position | Number of recommendation | Motivation |
| 1st position |  |  |
| 2nd position |  |  |
| 3rd position |  |  |

| 1. ***Management: Vaccination*** | | | | | |
| --- | --- | --- | --- | --- | --- |
| 17 | We recommend that all adults with CKD are offered annual **vaccination** with **influenza** vaccine, unless contraindicated. | KDIGO NfN | 2012 2015 | 1B 1B | **1 2 3 4 5 6 7 8 9  [---------------------------------------------------------]**  Poor Excellent *□Not judgeable*  **Rating based on:** *□ EMR extractability □ Relevance recommendation* |
| 18 | We recommend that all adults with eGFR < 30 ml/min/1.73 m2 (GFR categories G4-G5) and those at high risk of **pneumococcal infection** (e.g., nephrotic syndrome, diabetes, or those receiving immunosuppression) receive vaccination with polyvalent pneumococcal vaccine unless contraindicated. | KDIGO | 2012 | 1B | **1 2 3 4 5 6 7 8 9  [---------------------------------------------------------]**  Poor Excellent *□Not judgeable*  **Rating based on:** *□ EMR extractability □ Relevance recommendation* |
| 19 | We recommend that all adults who are at high risk of progression of CKD and have GFR < 30 ml/min/1.73 m2 (GFR categories G4-G5) be immunized against **hepatitis B** and the response confirmed by appropriate serological testing. (1B) | NfN KDIGO | 2015 2012 | 1B 1B | **1 2 3 4 5 6 7 8 9  [---------------------------------------------------------]**  Poor Excellent *□Not judgeable*  **Rating based on:** *□ EMR extractability □ Relevance recommendation* |

**Top recommendation**

Which recommendation for vaccination of chronic renal insufficiency do you find most suitable for measuring the quality of care?

| Top recommendation regarding vaccination. | | |
| --- | --- | --- |
| Position | Number of recommendation | Motivation |
| 1st position |  |  |

| 1. ***Treatment of CKD*** | | | | | |
| --- | --- | --- | --- | --- | --- |
| 20 | In patients with CKD, an ARB or an ACE inhibitor should be used in case of a strongly increased albuminuria (> 30 mg / mmol or> 300 mg / 24 hours) (grade 1B) and preferably also in case of moderately increased albuminuria (3-30 mg / mmol or 30-300 mg / 24 hours). | NfN | 2015 | 1B- 2D | **1 2 3 4 5 6 7 8 9  [---------------------------------------------------------]**  Poor Excellent *□Not judgeable*  **Rating based on:** *□ EMR extractability □ Relevance recommendation* |
| 21 | Treat all patients with a corrected proteinuria >  900 mg / g (100 mg / mmol) with an ACE-I regardless of blood pressure. | DM | 2012 | 1B | **1 2 3 4 5 6 7 8 9  [---------------------------------------------------------]**  Poor Excellent *□Not judgeable*  **Rating based on:** *□ EMR extractability □ Relevance recommendation* |
| 22 | An ACE inhibitor (ACE-I) is the preferred antihypertensive agent in all diabetic patients with CKD and in all patients with a corrected proteinuria > 270 mg / g (30 mg / mmol). | DM | 2012 | 2B | **1 2 3 4 5 6 7 8 9  [---------------------------------------------------------]**  Poor Excellent *□Not judgeable*  **Rating based on:** *□ EMR extractability □ Relevance recommendation* |
| 23 | Treat all diabetic patients with a corrected albuminuria > 20 mg / g (2.5 mg / mmol) in men and > 30 mg / g (3.5 mg / mmol) in women with an ACE-I regardless of blood pressure. | DM | 2012 | 2B | **1 2 3 4 5 6 7 8 9  [---------------------------------------------------------]**  Poor Excellent *□Not judgeable*  **Rating based on:** *□ EMR extractability □ Relevance recommendation* |
| 24 | Monitor serum potassium before and after initiating treatment with an ACE-I or ARB. In case of hyperpotassemia, first rule out any medical cause and then consider a diet to limit potassium intake. | DM | 2012 | 1C | **1 2 3 4 5 6 7 8 9  [---------------------------------------------------------]**  Poor Excellent *□Not judgeable*  **Rating based on:** *□ EMR extractability □ Relevance recommendation* |
| 25 | It is suggested to prevent serum potassium from exceeding >5.5 mmol / l to prevent the occurrence of cardiac arrhythmia. Both increased potassium (> 5.5 mmol / l) and reduced / low normal potassium (<4.0 mmol / l) are associated with mortality and cardiovascular endpoints. | NfN | 2015 | 2B | **1 2 3 4 5 6 7 8 9  [---------------------------------------------------------]**  Poor Excellent *□Not judgeable*  **Rating based on:** *□ EMR extractability □ Relevance recommendation* |
| 26 | To reduce the potassium levels the following measures are advised:   - a dietary potassium limitation between 2000-3000 mg [50-75 mmol] per day - adjustment of potassium-increasing medication - correction of metabolic acidosis - the use of potassium binders (preferably non-sodium binders) (if necessary) | NfN | 2015 | 1C | **1 2 3 4 5 6 7 8 9  [---------------------------------------------------------]**  Poor Excellent *□Not judgeable*  **Rating based on:** *□ EMR extractability □ Relevance recommendation* |
| 27 | In patients with CKD and a serum bicarbonate <20 mmol / L treatment should be initiated using an oral bicarbonate to keep the serum bicarbonate level within the normal range. | NfN  KDIGO | 2015  2012 | 2B  2B | **1 2 3 4 5 6 7 8 9  [---------------------------------------------------------]**  Poor Excellent *□Not judgeable*  **Rating based on:** *□ EMR extractability □ Relevance recommendation* |
| **5.1. Treatment: Patient education and information** | | | | | |
| 28 | Offer tailored education and support programs in the self-management of CKD patients (GPP). Referral to a specialized nurse is recommended in order to ameliorate understanding of their condition, to ameliorate compliance to lifestyle changes and drug treatment (GRADE 1C). These measures are taken to stabilize parameters and to preserve renal function as long as possible (GPP). | EBM | 2017 | GPP 1C GPP | **1 2 3 4 5 6 7 8 9  [---------------------------------------------------------]**  Poor Excellent *□Not judgeable*  **Rating based on:** *□ EMR extractability □ Relevance recommendation* |
| 29 | Provide the following support: information for patients with CKD about their condition, a program for 'shared decision making', support for self-management (eg blood pressure, smoking cessation, exercise, diet and medication) and support in making a well informed choice. | EBM | 2017 | 2B | **1 2 3 4 5 6 7 8 9  [---------------------------------------------------------]**  Poor Excellent *□Not judgeable*  **Rating based on:** *□ EMR extractability □ Relevance recommendation* |
| **5.2. Treatment: Lifestyle and diet** | | | | | |
| 30 | Patients with CKD should be encouraged to (GRADE 1B):   - Undertake physical activity compatible with cardiovascular health and tolerance (aiming for at least 30 minutes 5 times per week) - Stop smoking - Obtain or maintain a healthy weight   - (BMI 20 to 25, depending on country-specific demographics).   - Waist circumference:     - Waist circumference ≥94 cm in men or ≥80 cm in women (SIGN 103: GPP)     - Waist circumference < 102 cm for men, < 88 cm for women (CMAJ 2008: D) - Limit the alcohol intake (only mentioned in NfN, CMAJ) | EBM NICE CG182  SIGN 103 KDIGO NfN MSN DM  CMAJ | 2017 2014 (2015 update) 2008  2012 2015 2011 2012  2008 | 1B None  GPP  1D 1D B 1B-1C (roken)-1B D-D-D-B | **1 2 3 4 5 6 7 8 9  [---------------------------------------------------------]**  Poor Excellent *□Not judgeable*  **Rating based on:** *□ EMR extractability □ Relevance recommendation* |
| 31 | For CNI stage 1 to 3, no specific dietary advice other than the healthy diet recommended to the general population is required, except for patients with hypertension (low salt) or hypercholesterolemia (low saturated fatty acids). | EBM | 2017 | GPP | **1 2 3 4 5 6 7 8 9  [---------------------------------------------------------]**  Poor Excellent *□Not judgeable*  **Rating based on:** *□ EMR extractability □ Relevance recommendation* |
| 32 | Individuals with CKD at high risk should receive expert dietary advice and information in the context of an education program, tailored to severity of CKD and the need to intervene on salt, phosphate, potassium, and protein intake where indicated. | NfN  KDIGO  EBM | 2015  2012  2017 | 1B  1B  1B | **1 2 3 4 5 6 7 8 9  [---------------------------------------------------------]**  Poor Excellent *□Not judgeable*  **Rating based on:** *□ EMR extractability □ Relevance recommendation* |
| 33 | Complex diets for some patients with severe CKD require specialized guidance by a dietician to prevent dietary errors and/or malnutrition. Dietary advice about potassium and phosphate intake tailored to CKD stage is made by an appropriately qualified **dietitian**. | EBM | 2017 | 1B | **1 2 3 4 5 6 7 8 9  [---------------------------------------------------------]**  Poor Excellent *□Not judgeable*  **Rating based on:** *□ EMR extractability □ Relevance recommendation* |
| **5.3. Treatment: Diabetes and glycemic control** | | | | | |
| 34 | We recommend a target hemoglobin A1c (HbA1c) of ~7.0% (53 mmol/mol) to prevent or delay progression of the microvascular complications of diabetes, including diabetic kidney disease. | NfN  KDIGO | 2015  2012 | 1A  1A | **1 2 3 4 5 6 7 8 9  [---------------------------------------------------------]**  Poor Excellent *□Not judgeable*  **Rating based on:** *□ EMR extractability □ Relevance recommendation* |
| 35 | An HbA1c level of <53 mmol / mol (<7.0%) should not be strived for in the following circumstances:   - patients at risk of hypoglycaemia (grade 1B) - people with co-morbidity or limited life expectancy and risk of hypoglycaemia (2C) | NfN  KDIGO | 2015  2012 | 1B/2C  1B/2C | **1 2 3 4 5 6 7 8 9  [---------------------------------------------------------]**  Poor Excellent *□Not judgeable*  **Rating based on:** *□ EMR extractability □ Relevance recommendation* |
| 36 | In the context of drug management and patient safety, we recommend the use of metformin in patients with an eGFR >45ml/min./1.73m²; Metformin use has to be evaluated if eGFR is between 30-44ml/min./1.73m². Metformin has to be avoided with an eGFR <30 ml/min./1.73m². | DM | 2012 | 1C | **1 2 3 4 5 6 7 8 9  [---------------------------------------------------------]**  Poor Excellent *□Not judgeable*  **Rating based on:** *□ EMR extractability □ Relevance recommendation* |
| **5.4. Treatment dyslipidemia** | | | | | |
| 37 | In adults with CKD it is recommended to determine a one time lipid profile (total cholesterol, LDL cholesterol, HDL cholesterol, triglycerides). | NfN | 2015 | 1C | **1 2 3 4 5 6 7 8 9  [---------------------------------------------------------]**  Poor Excellent *□Not judgeable*  **Rating based on:** *□ EMR extractability □ Relevance recommendation* |
| 38 | Offer **atorvastatin 20 mg** for the primary or secondary prevention of **CVD** to people with CKD.   - Increase the dose if a greater than 40% reduction in non‑HDL cholesterol is not achieved (see recommendation 1.3.28) and eGFR is 30 ml/min/1.73 m^2^ or more. - Agree the use of higher doses with a renal specialist if eGFR is less than 30 ml/min/1.73 m^2^ | NICE CG181 | 2014 (2016 update) | None | **1 2 3 4 5 6 7 8 9  [---------------------------------------------------------]**  Poor Excellent *□Not judgeable*  **Rating based on:** *□ EMR extractability □ Relevance recommendation* |
| 39 | In adults aged ≥50 years with an **eGFR <60** ml/min/1.73 m2 treatment with **statines or a combination** statine/ezetimibe is advised. | NfN | 2015 | 1A | **1 2 3 4 5 6 7 8 9  [---------------------------------------------------------]**  Poor Excellent *□Not judgeable*  **Rating based on:** *□ EMR extractability □ Relevance recommendation* |
| 40 | In adults aged ≥50 years with CKD and an **eGFR <60** ml/min/1.73 m2  treatment with statines is advised. | NfN | 2015 | 1B | **1 2 3 4 5 6 7 8 9  [---------------------------------------------------------]**  Poor Excellent *□Not judgeable*  **Rating based on:** *□ EMR extractability □ Relevance recommendation* |
| 41 | In adults aged 18–49 years with CKD treatment with **statines** is suggested if one or more of the following conditions apply:   - history of coronary heart disease (myocardal infarction or previous coronary revascularization) - diabetes mellitus - previous ischemic stroke - estimated 10 year risk of fatal coronary disease or non-fatal myocardial infarction >10% | NfN | 2015 | 2A | **1 2 3 4 5 6 7 8 9  [---------------------------------------------------------]**  Poor Excellent *□Not judgeable*  **Rating based on:** *□ EMR extractability □ Relevance recommendation* |
| 42 | Offer **atorvastatin 20 mg** for the primary or secondary prevention of **CVD** to people with CKD.   - Increase the dose if a greater than 40% reduction in non‑HDL cholesterol is not achieved (see recommendation 1.3.28) and eGFR is 30 ml/min/1.73 m^2^ or more. - Agree the use of higher doses with a renal specialist if eGFR is less than 30 ml/min/1.73 m^2^ | NICE CG181 | 2014 (2016 update) | None | **1 2 3 4 5 6 7 8 9  [---------------------------------------------------------]**  Poor Excellent *□Not judgeable*  **Rating based on:** *□ EMR extractability □ Relevance recommendation* |

| **5.5. Treatment of hypertension** | | | | | |
| --- | --- | --- | --- | --- | --- |
| 43 | Target BP should be **<130/80** (SBP range 120 - 129) mmHg   - In patients with proteinuria ≥1 g/day. (MSN, SIGN Grade A) - In patients with normal urinary albumin concentrations (AAFP Grade B) - In patients with diabetes. (MSN Grade B, CMAJ grade B) - In patients with a strongly increased albuminuria, first choice antihypertensive drugs to achieve these goals are ACE inhibitors or ARB's (NfN grade 1B, KDIGO grade 2D) - in people with **ACR**  **≥70** mg/mmol (NICE CG182, none) | MSN SIGN AAFP CMAJ NfN KDIGONICE CG182 | 2011 2008 2005 2008 2015 2012 2014 (2015 upd) | A A  B B | **1 2 3 4 5 6 7 8 9  [---------------------------------------------------------]**  Poor Excellent *□Not judgeable*  **Rating based on:** *□ EMR extractability □ Relevance recommendation* |
| 44 | We recommend that in both diabetic and non-diabetic adults with CKD and urine albumin excretion <30 mg/ 24 hours (or <3mg/mmol) whose office BP is consistently >140 mm Hg systolic or >90 mm Hg diastolic be treated with BP-lowering drugs to maintain a BP that is consistently **≤**140 mm Hg systolic and **≤**90 mm Hg diastolic. (1B) | NfN KDIGO NICE CG182  MSN DM | 2015 2012 2014 (2015 update) 2011 2012 | 1B 1B None  A 1B | **1 2 3 4 5 6 7 8 9  [---------------------------------------------------------]**  Poor Excellent *□Not judgeable*  **Rating based on:** *□ EMR extractability □ Relevance recommendation* |
| 45 | Any class of **antihypertensive** agents can be used to treat hypertension in chronic kidney disease (CKD) patients without proteinuria. The choice will depend on the patient’s co-morbidity. | MSN | 2011 | C | **1 2 3 4 5 6 7 8 9  [---------------------------------------------------------]**  Poor Excellent *□Not judgeable*  **Rating based on:** *□ EMR extractability □ Relevance recommendation* |
| 46 | ACP recommends that clinicians select pharmacologic therapy that includes either an **ACEi** (moderate-quality evidence) or an **ARB** (high-quality evidence) in patients with **hypertension** and stage 1 to 3 chronic kidney disease. | ACP/AAFP AnnalsAAFP | 2013  2013 2014 | strong recommendation | **1 2 3 4 5 6 7 8 9  [---------------------------------------------------------]**  Poor Excellent *□Not judgeable*  **Rating based on:** *□ EMR extractability □ Relevance recommendation* |
| **5.6. Treatment of cardiovascular disease** | | | | | |
| 47 | We suggest that adults with CKD at risk for **atherosclerotic events** be offered treatment with **antiplatelet agents** unless there is an increased bleeding risk that needs to be balanced against the possible cardiovascular benefits. | KDIGO | 2012 | 2B | **1 2 3 4 5 6 7 8 9  [---------------------------------------------------------]**  Poor Excellent *□Not judgeable*  **Rating based on:** *□ EMR extractability □ Relevance recommendation* |
| 48 | In people with **CKD and heart failure**, any escalation in therapy and/or clinical deterioration should promptmonitoring of eGFR and serum potassium concentration. | KDIGO | 2012 | None | **1 2 3 4 5 6 7 8 9  [---------------------------------------------------------]**  Poor Excellent *□Not judgeable*  **Rating based on:** *□ EMR extractability □ Relevance recommendation* |
| 49 | Offer antiplatelet drugs (acetylsalicylic acid) to people with CKD for the secondary prevention of cardiovascular disease, but be aware of the increased risk of bleeding. | NfN  MSN  NICE CG 182 | 2015  2011  2014 (2015 update) | 1B  B  None | **1 2 3 4 5 6 7 8 9  [---------------------------------------------------------]**  Poor Excellent *□Not judgeable*  **Rating based on:** *□ EMR extractability □ Relevance recommendation* |
| 50 | Combination of **clopidogrel with aspirin** should be avoided in patients with CKD (unless compelling indications are present). (Grade B) | MSN | 2011 | B | **1 2 3 4 5 6 7 8 9  [---------------------------------------------------------]**  Poor Excellent *□Not judgeable*  **Rating based on:** *□ EMR extractability □ Relevance recommendation* |
| 51 | Consider **apixaban** in preference to **warfarin** in people with a confirmed eGFR of 30–50 ml/min/1.73 m^2^ and non-valvular atrial fibrillation who have 1 or more of the following risk factors:   - prior stroke or transient ischaemic attack - age 75 years or older - hypertension - diabetes mellitus - symptomatic heart failure | NICE CG182 | 2014 (2015 update) | None | **1 2 3 4 5 6 7 8 9  [---------------------------------------------------------]**  Poor Excellent *□Not judgeable*  **Rating based on:** *□ EMR extractability □ Relevance recommendation* |
| **5.7. Treatment anemia** | | | | | |
| 52 | If not already measured, check the haemoglobin level in people with a GFR < 45 ml/min/1.73 m^2^ (GFR category G3b, G4 or G5) to identify anaemia (haemoglobin less than 110 g/litre [11.0 g/dl]). Determine the subsequent frequency of testing by the measured value and the clinical circumstances. | NICE CG182  DM | 2014 (2015 update) 2012 | None  1C | **1 2 3 4 5 6 7 8 9  [---------------------------------------------------------]**  Poor Excellent *□Not judgeable*  **Rating based on:** *□ EMR extractability □ Relevance recommendation* |
| 53 | Offer EPO and iron to treat anemia of renal origin. | DM | 2012 | 1C | **1 2 3 4 5 6 7 8 9  [---------------------------------------------------------]**  Poor Excellent *□Not judgeable*  **Rating based on:** *□ EMR extractability □ Relevance recommendation* |
| 54 | A trial of oral or IV iron is suggested to patients with anemia without iron supplementation and with/without ESA if an increase in Hb concentration is preferred without initiating or increasing ESA dosage, transferrinesaturation ≤25% and ferritine concentration<200 mg/l is. | NfN | 2015 | 2C | **1 2 3 4 5 6 7 8 9  [---------------------------------------------------------]**  Poor Excellent *□Not judgeable*  **Rating based on:** *□ EMR extractability □ Relevance recommendation* |
| **5.8. Treatment mineral metabolism abnormalities** | | | | | |
| 55 | Measure serum calcium, phosphate and PTH concentrations in people with a GFR of less than 30 ml/min/1.73 m2 (GFR category G4 or G5). Determine the subsequent frequency of testing by the measured values and the clinical circumstances. Where doubt exists, seek specialist opinion. | DM  NICE CG182 | 2012  2014 (update 2015) | 1C  None | **1 2 3 4 5 6 7 8 9  [---------------------------------------------------------]**  Poor Excellent *□Not judgeable*  **Rating based on:** *□ EMR extractability □ Relevance recommendation* |
| 56 | Do not routinely measure calcium, phosphate, parathyroid hormone (PTH) and vitamin D levels in people with a GFR of 30 ml/min/1.73 m^2^ or more (GFR category G1, G2 or G3). | NICE CG182 | 2014 (2015 update) | None | **1 2 3 4 5 6 7 8 9  [---------------------------------------------------------]**  Poor Excellent *□Not judgeable*  **Rating based on:** *□ EMR extractability □ Relevance recommendation* |
| 57 | Consider offering **vitamin D** if there is a 25-OH-vitamine D deficiency or an increase of PTH (Grade 2B): ∙   - **Colecalciferol** or **ergocalciferol** in patients with CKD stage 1, 2 and 3 (GFR ≥30 ml/min/1,73 m2 ) and a vitamin D deficiency; - **Alfacalcidol** or **calcitrol in patients with CKD stage** 4 and 5 (GFR <30 ml/min/1,73 m2 ) with secundary hyperparathyroidism despite treatment with colecalciferol of ergocalciferol. - Monitor serum calcium and phosphate in these cases. | DM  NICE CG 182 | 2012  2014 (2015 update) | 2B  None | **1 2 3 4 5 6 7 8 9  [---------------------------------------------------------]**  Poor Excellent *□Not judgeable*  **Rating based on:** *□ EMR extractability □ Relevance recommendation* |
| 58 | Do not prescribe **bisphosphonate treatment** in people with GFR 30 ml/min/1.73 m2 (GFR categories G4-G5) without a strong clinical rationale. | KDIGO | 2012 | 2B | **1 2 3 4 5 6 7 8 9  [---------------------------------------------------------]**  Poor Excellent *□Not judgeable*  **Rating based on:** *□ EMR extractability □ Relevance recommendation* |
| 59 | Offer **bisphosphonates** if indicated for the prevention and treatment of osteoporosis in people with a GFR of 30 ml/min/1.73 m^2^ or more (GFR category G1, G2 or G3) | NICE CG182 | 2014 (2015 update) | None | **1 2 3 4 5 6 7 8 9  [---------------------------------------------------------]**  Poor Excellent *□Not judgeable*  **Rating based on:** *□ EMR extractability □ Relevance recommendation* |

**Top 5 recommendations**

Which recommendations for the treatment of chronic renal insufficiency do you find most suitable for measuring the quality of care?

| Top 5 recommendations regarding treatment. | | |
| --- | --- | --- |
| Position | Number of recommendation | Motivation |
| 1st position |  |  |
| 2nd position |  |  |
| 3rd position |  |  |
| 4th position |  |  |
| 5th position |  |  |

| 1. ***Medication and patient safety*** | | | | | |
| --- | --- | --- | --- | --- | --- |
| 60 | In patients with heart failure, prescribe diuretics at the lowest effective dose, slowly alter doses and only use diuretics in case of symptoms of fluid retention. | DM | 2012 | 1C | **1 2 3 4 5 6 7 8 9  [---------------------------------------------------------]**  Poor Excellent *□Not judgeable*  **Rating based on:** *□ EMR extractability □ Relevance recommendation* |
| 61 | In patiënts with heart failure and CKD, avoid the use of spironolactone. | DM | 2012 | 2C | **1 2 3 4 5 6 7 8 9  [---------------------------------------------------------]**  Poor Excellent *□Not judgeable*  **Rating based on:** *□ EMR extractability □ Relevance recommendation* |
| 62 | In patients with heart failure and CKD, avoid the use of digoxin due to the higher risk of intoxication. When digoxin use is required, reduced doses will be administered. | DM | 2012 | 2C | **1 2 3 4 5 6 7 8 9  [---------------------------------------------------------]**  Poor Excellent *□Not judgeable*  **Rating based on:** *□ EMR extractability □ Relevance recommendation* |
| 63 | In people with CKD, measure serum **potassium** concentrations and estimate the **GFR before** starting renin–angiotensin system antagonists. Repeat these measurements between 1 and **2 weeks** after starting renin–angiotensin system antagonists and after each dose increase. | NICE CG182  MSN | 2014 (2015 update)  2011 | None  B | **1 2 3 4 5 6 7 8 9  [---------------------------------------------------------]**  Poor Excellent *□Not judgeable*  **Rating based on:** *□ EMR extractability □ Relevance recommendation* |
| 64 | If there is a sustained **rise** in **creatinine** levels above 30% (or estimated glomerular filtration rate reduces >25%) from the baseline or serum **potassium is >5.6 mmol/l** during the first two months after commencement of ACEi/ARB therapy, reduce or **discontinue** the **ACEi/ARB** after excluding other precipitating factors and refer to a nephrologist/physician. (Grade B) | MSN | 2011 | B | **1 2 3 4 5 6 7 8 9  [---------------------------------------------------------]**  Poor Excellent *□Not judgeable*  **Rating based on:** *□ EMR extractability □ Relevance recommendation* |
| 65 | If there is a decrease in eGFR or increase in serum creatinine after starting or increasing the dose of renin–angiotensin system antagonists, but **<25%** (eGFR) or **<30%** (serum creatinine) of baseline, **repeat** the **test** in 1–2 weeks. Do not modify the renin–angiotensin system antagonist dose if the change in eGFR is <25% or the change in serum creatinine is <30%. | NICE CG182 | 2014 (2015 update) | None | **1 2 3 4 5 6 7 8 9  [---------------------------------------------------------]**  Poor Excellent *□Not judgeable*  **Rating based on:** *□ EMR extractability □ Relevance recommendation* |
| 66 | We recommend temporary discontinuation of potentially nephrotoxic and renally excreted drugs in people with a GFR <60 ml/min/1.73 m2 (GFR categories G3a-G5) who have serious intercurrent illness that increases the risk of AKI. These agents include, but are not limited to: RAAS blockers (including ACE-Is, ARBs, aldosterone inhibitors, direct renin inhibitors), diuretics, NSAIDs, metformin, lithium, and digoxin. | EBM  NfN  KDIGO | 2017  2015  2012 | 1C  1C  1C | **1 2 3 4 5 6 7 8 9  [---------------------------------------------------------]**  Poor Excellent *□Not judgeable*  **Rating based on:** *□ EMR extractability □ Relevance recommendation* |
| 67 | We recommend not using fytotherapy in people with CKD. (1B) | EBM | 2017 | 1B | **1 2 3 4 5 6 7 8 9**  **[---------------------------------------------------------]**  Poor Excellent *□Not judgeable*  **Rating based on:** *□ EMR extractability □ Relevance recommendation* |

| **6.1. Safety: imaging and contrast agents** | | | | | |
| --- | --- | --- | --- | --- | --- |
| 68 | Determine the eGFR before each examination with contrast agent, if no recent (last 12 months) value is known. | DM | 2012 | 1B | **1 2 3 4 5 6 7 8 9  [---------------------------------------------------------]**  Poor Excellent *□Not judgeable*  **Rating based on:** *□ EMR extractability □ Relevance recommendation* |
| 69 | Inform the performer of any examination with a contrast agent of the patients renal function and discuss the preventive measures that have to be taken. | DM | 2012 | 1B | **1 2 3 4 5 6 7 8 9  [---------------------------------------------------------]**  Poor Excellent *□Not judgeable*  **Rating based on:** *□ EMR extractability □ Relevance recommendation* |
| 70 | Do not use gadolinium-containing contrast media in people with GFR < 15 ml/min/1.73 m2 (GFR category G5) unless there is no alternative appropriate test. | NfN  KDIGO | 2015  2012 | 1B  1B | **1 2 3 4 5 6 7 8 9  [---------------------------------------------------------]**  Poor Excellent *□Not judgeable*  **Rating based on:** *□ EMR extractability □ Relevance recommendation* |
| 71 | Do not use **oral phosphate-containing bowel preparations** in people with a **GFR < 60** ml/min/1.73 m2 (GFR categories G3a-G5) or in those known to be at risk of phosphate nephropathy. | KDIGO | 2012 | 1A | **1 2 3 4 5 6 7 8 9  [---------------------------------------------------------]**  Poor Excellent *□Not judgeable*  **Rating based on:** *□ EMR extractability □ Relevance recommendation* |

**Top 5 recommendations**

Which recommendations for medication and safety of the patient of chronic renal insufficiency do you find most suitable for measuring the quality of care?

| Top 5 recommendations regarding medication and safety of the patient. | | |
| --- | --- | --- |
| Position | Number of recommendation | Motivation |
| 1st position |  |  |
| 2nd position |  |  |
| 3rd position |  |  |
| 4th position |  |  |
| 5th position |  |  |

| 1. ***Referral to specialist*** | | | | | |
| --- | --- | --- | --- | --- | --- |
| **For patients with CKD, referral to a nephrologist or specialist with specific knowledge of CKD is advised in the following cases:** | | | | | |
| 71.1 | Acute kidney injury or abrupt sustained fall in GFR. | NfN, KDIGO | 2015 2012 | 1B 1B | **1 2 3 4 5 6 7 8 9  [---------------------------------------------------------]**  Poor Excellent *□Not judgeable*  **Rating based on:** *□ EMR extractability □ Relevance recommendation* |
| 71.2 | GFR <30 ml/min/1,73 m2 (GFR categorie G4 or G5). | NfN, KDIGOAAFP AAFP NICE CG 182  MSN DM | 2015 2012 2004 (I) 2011 2014 (2015 update) 2011 2012 | 1B 1B C C none   C 2B | **1 2 3 4 5 6 7 8 9  [---------------------------------------------------------]**  Poor Excellent *□Not judgeable*  **Rating based on:** *□ EMR extractability □ Relevance recommendation* |
| 71.3 | Patients <75 jaar with an eGFR between 30 and 45 ml/min./1,73 m² and an ACR of 20-200mg/g for males and 30-300 mg/g for females. | EBM  DM | 2017  2012 | 2B  2B | **1 2 3 4 5 6 7 8 9  [---------------------------------------------------------]**  Poor Excellent *□Not judgeable*  **Rating based on:** *□ EMR extractability □ Relevance recommendation* |
| 71.4 | Patients with an eGFR >45 ml/min./1,73 m² and an ACR >200 mg/g for males or 300 mg/g for females and/or a PCR >1 000 mg/g. | EBM  DM | 2017  2012 | 2B  2B | **1 2 3 4 5 6 7 8 9  [---------------------------------------------------------]**  Poor Excellent *□Not judgeable*  **Rating based on:** *□ EMR extractability □ Relevance recommendation* |

| 71.5 | A consistent finding of significant albuminuria (ACR ≥300 mg/g [≥30 mg/mmol] or AER ≥ 300 mg/ 24 hours, approximately equivalent to PCR ≥ 500 mg/g [≥50 mg/mmol] or PER ≥500 mg/24 hours | NfN, KDIGO, EBM | 2015 2012 2017 | 1B 1B 1B | **1 2 3 4 5 6 7 8 9  [---------------------------------------------------------]**  Poor Excellent *□Not judgeable*  **Rating based on:** *□ EMR extractability □ Relevance recommendation* |
| --- | --- | --- | --- | --- | --- |
| 71.6 | Heavy proteinuria (urine protein ≥1 g/day or urine protein: creatinine ratio (uPCR) ≥0.1 g/mmol) unless known to be due to diabetes and optimally treated | MSN | 2011 | C | **1 2 3 4 5 6 7 8 9  [---------------------------------------------------------]**  Poor Excellent *□Not judgeable*  **Rating based on:** *□ EMR extractability □ Relevance recommendation* |
| 71.7 | Haematuria with proteinuria (urine protein ≥0.5 g/day or uPCR ≥0.05 g/mmol) | MSN | 2011 | C | **1 2 3 4 5 6 7 8 9  [---------------------------------------------------------]**  Poor Excellent *□Not judgeable*  **Rating based on:** *□ EMR extractability □ Relevance recommendation* |
| 71.8 | ACR ≥ 70 mg/mmol, unless known to be caused by diabetes and already appropriately treated | NICE CG182 | 2014 (update 2015) | None | **1 2 3 4 5 6 7 8 9  [---------------------------------------------------------]**  Poor Excellent *□Not judgeable*  **Rating based on:** *□ EMR extractability □ Relevance recommendation* |
| 71.9 | ACR ≥ 30 mg/mmol or more (ACR category A3), together with haematuria | NICE CG182 | 2014 (update 2015) | None | **1 2 3 4 5 6 7 8 9  [---------------------------------------------------------]**  Poor Excellent *□Not judgeable*  **Rating based on:** *□ EMR extractability □ Relevance recommendation* |
| 71.10 | Progression of CKD: confirmed decline in GFR category accompanied by a 25% or greater drop in eGFR from baseline or a sustained decline in eGFR of more than 5 ml/min/1.73 m2 /year. | NfN, KDIGO | 2015 2012 | 1B 1B | **1 2 3 4 5 6 7 8 9  [---------------------------------------------------------]**  Poor Excellent *□Not judgeable*  **Rating based on:** *□ EMR extractability □ Relevance recommendation* |
| 71.11 | Patients with progressive CKD, being an eGFR between 30 en 45 ml/min./1,73 m² and a decline of eGFR >10 ml/min in five years time or > 5 ml/min in two years time. | EBM  DM | 2017  2012 | 2B  2B | **1 2 3 4 5 6 7 8 9  [---------------------------------------------------------]**  Poor Excellent *□Not judgeable*  **Rating based on:** *□ EMR extractability □ Relevance recommendation* |
| 71.12 | Rapidly declining renal function (loss of glomerular filtration rate/GFR >5 ml/min/1.73m2 in one year or >10 ml/min/1.73m2 Within five years) | MSN | 2011 | C | **1 2 3 4 5 6 7 8 9  [---------------------------------------------------------]**  Poor Excellent *□Not judgeable*  **Rating based on:** *□ EMR extractability □ Relevance recommendation* |
| 71.13 | Sustained decrease in GFR of 25% or more, and a change in GFR category or sustained decrease in GFR of 15 ml/min/1.73 m^2^ or more within 12 months | NICE CG182 | 2014 (update 2015) | none | **1 2 3 4 5 6 7 8 9  [---------------------------------------------------------]**  Poor Excellent *□Not judgeable*  **Rating based on:** *□ EMR extractability □ Relevance recommendation* |
| 71.14 | Dysmorphic  erytrocytes in urine sedimentation (>20 pgv). | NfN, KDIGO | 2015,2012 | 1B,1B | **1 2 3 4 5 6 7 8 9  [---------------------------------------------------------]**  Poor Excellent *□Not judgeable*  **Rating based on:** *□ EMR extractability □ Relevance recommendation* |
| 71.15 | Patients with hematuria of unknown origin (GRADE 1B); | EBM | 2017 | 1B | **1 2 3 4 5 6 7 8 9  [---------------------------------------------------------]**  Poor Excellent *□Not judgeable*  **Rating based on:** *□ EMR extractability □ Relevance recommendation* |
| 71.16 | CKD combined with hypertension, which insufficiëntly responds to medical treatment with 4 or more antihypertensive drugs. | NfN KDIGO EBM MSN NICE CG182 | 2015 2012 2017 2011 2014 (update 2015) | 1B 1B 1B C none | **1 2 3 4 5 6 7 8 9  [---------------------------------------------------------]**  Poor Excellent *□Not judgeable*  **Rating based on:** *□ EMR extractability □ Relevance recommendation* |
| 71.17 | Persisting serum potassium abnormalities. | NfN KDIGO EBM | 2015 2012 2017 | 1B 1B 1B | **1 2 3 4 5 6 7 8 9  [---------------------------------------------------------]**  Poor Excellent *□Not judgeable*  **Rating based on:** *□ EMR extractability □ Relevance recommendation* |
| 71.18 | Recurrent or extensive nefrolithiasis. | NfN KDIGO EBM | 2015 2012 2017 | 1B 1B 1B | **1 2 3 4 5 6 7 8 9  [---------------------------------------------------------]**  Poor Excellent *□Not judgeable*  **Rating based on:** *□ EMR extractability □ Relevance recommendation* |
| 71.19 | Hereditary kidney disease. | NfN KDIGO EBM MSN NICE CG182 | 2012 2015 2017 2011 2014 (update 2015) | 1B 1B 1B C none | **1 2 3 4 5 6 7 8 9  [---------------------------------------------------------]**  Poor Excellent *□Not judgeable*  **Rating based on:** *□ EMR extractability □ Relevance recommendation* |
| 71.20 | When a.renalis stenosis is suspected or established. | MSN EBM | 2011 2017 | C GPP | **1 2 3 4 5 6 7 8 9  [---------------------------------------------------------]**  Poor Excellent *□Not judgeable*  **Rating based on:** *□ EMR extractability □ Relevance recommendation* |
| 71.21 | For treatment of complications of kidney failure: anemia, electrolyte imbalance, vitamin D, calcium- and phosphate disturbances, uremic complaints (pruritus,…). | EBM | 2017 | GPP | **1 2 3 4 5 6 7 8 9  [---------------------------------------------------------]**  Poor Excellent *□Not judgeable*  **Rating based on:** *□ EMR extractability □ Relevance recommendation* |
| 71.22 | Suspected glomerular disease. | MSN NICE CG182 | 2011 2014 (update 2015) | C none | **1 2 3 4 5 6 7 8 9  [---------------------------------------------------------]**  Poor Excellent *□Not judgeable*  **Rating based on:** *□ EMR extractability □ Relevance recommendation* |
| 71.23 | Pregnant or when pregnancy is planned. | MSN | 2011 | C | **1 2 3 4 5 6 7 8 9  [---------------------------------------------------------]**  Poor Excellent *□Not judgeable*  **Rating based on:** *□ EMR extractability □ Relevance recommendation* |
| 71.24 | Unclear cause of CKD. | MSN | 2011 | C | **1 2 3 4 5 6 7 8 9  [---------------------------------------------------------]**  Poor Excellent *□Not judgeable*  **Rating based on:** *□ EMR extractability □ Relevance recommendation* |
| 72 | Refer high risk patiënts for inclusion in the care program. These are patients with:   - a chronic eGFR <30 ml/min./1,73 m² (eGFR categories G4-G5) (GRADE 1B); - an eGFR between 30-45 ml/min./1,73 m² and ACR >200 mg/g for males or 300 mg/g for females, and/or proteïnuria >1000 mg/24h or a protein-creatininratio (PCR) >1 000 mg/g (GRADE 2B). | EBM | 2017 | 1B-2B | **1 2 3 4 5 6 7 8 9  [---------------------------------------------------------]**  Poor Excellent *□Not judgeable*  **Rating based on:** *□ EMR extractability □ Relevance recommendation* |
| **7.*1. Renal replacement therapy*** | | | | | |
| 73 | We recommend timely referral for planning renal replacement therapy (RRT) in people with progressive CKD in whom the risk of kidney failure within 1 year is 10–20% or higher , as determined by validated risk prediction tools. (1B) | EBM  KDIGO | 2017  2012 | 1B  1B | **1 2 3 4 5 6 7 8 9  [---------------------------------------------------------]**  Poor Excellent *□Not judgeable*  **Rating based on:** *□ EMR extractability □ Relevance recommendation* |

**Top 5 recommendation**

Which recommendations for refferal to the specialist of chronic renal insufficiency do you find most suitable for measuring the quality of care?

| Top 5 recommendation regarding refferal to the specialist. | | |
| --- | --- | --- |
| Position | Number of recommendation | Motivation |
| 1st position |  |  |
| 2nd position |  |  |
| 3rd position |  |  |
| 4th position |  |  |
| 5th position |  |  |

| 1. ***Role of the general practitioner and care program*** | | | | | |
| --- | --- | --- | --- | --- | --- |
| 74 | We suggest that people with progressive CKD should be managed in a multidisciplinary care setting. (2B) The multidisciplinary team should include or have access to dietary counseling, education and counseling about different RRT modalities, transplant options, vascular access surgery, and ethical, psychological, and social care. | EBM KDIGO | 2017 2012 | 2B – GPP None | **1 2 3 4 5 6 7 8 9  [---------------------------------------------------------]**  Poor Excellent *□Not judgeable*  **Rating based on:** *□ EMR extractability □ Relevance recommendation* |
| 75 | The comprehensive conservative management program should include protocols for symptom and pain management, psychological care, spiritual care, and culturally sensitive care for the dying patient and their family (whether at home, in a hospice or a hospital setting), followed by the provision of culturally appropriate bereavement support. | EBM  KDIGO  CMAJ | 2017  2012  2008 | GPP  None  D | **1 2 3 4 5 6 7 8 9  [---------------------------------------------------------]**  Poor Excellent *□Not judgeable*  **Rating based on:** *□ EMR extractability □ Relevance recommendation* |

**Top recommendation**

Which recommendations for the role of the general practitioner and care program of chronic renal insufficiency do you find most suitable for measuring the quality of care?

| Top recommendation regarding the role of the general practitioner and care program. | | |
| --- | --- | --- |
| Position | Number of recommendation | Motivation |
| 1st position |  |  |

**Suggestions**

If you have any suggestions for recommendations which you think are missing in the foregoing list, please write them down in this table.

| Position | Recommendation | Motivation |
| --- | --- | --- |
| 1) |  |  |
| 2) |  |  |
| 3) |  |  |
| 4) |  |  |
| 5) |  |  |
| 6) |  |  |
| 7) |  |  |
| 8) |  |  |
| 9) |  |  |
| 10) |  |  |

**Appendix 1: Explanation of the grading systems**

This appendix explains the different grading systems used in the guidelines. The National Institute for Health and Care Excellence (NICE) don’t use a grading system.

**Domus Medica**

The level of evidence is represented by the letters A, B and C; A has the highest level of evidence, C the lowest. The numbers 1 and 2 are associated with the letters; 1 has a high and 2 a low grade of recommendation.

|  | Level of evidence | Benefits versus disadvantages |
| --- | --- | --- |
| 1A | Strong recommendation  High level of evidence | Benefits > disadvantages and risks |
| 1B | Moderate level of evidence |  |
| 1C | Low level of evidence |  |
| 2A | Weak recommendation, high level of evidence | Benefits = disadvantages (in balance) |
| 2B | Weak recommendation, moderate level of evidence |  |
| 2C | Weak recommendation, low level of evidence | Balance or uncertainty about benefits and disadvantages. |
| GPP | "Good Practice Point"  (consensus) |  |

**Scottish Intercollegiate Guidelines Network (SIGN)**

The authors of the SIGN guideline divide the used sources according to the level of evidence. They use the level of evidence to assign a certain degree to each recommendation.

| Levels of evidence | |
| --- | --- |
| Level | Explanation |
| 1++ | High quality meta-analyses, systematic reviews or RCTs, or RCTs with a very low risk of bias |
| 1+ | Well conducted meta-analyses, systematic reviews, or RCTs with a low risk of bias |
| 1- | Meta-analyses, systematic reviews, or RCTs with a high risk of bias |
| 2++ | High quality systematic reviews of case control or cohort studies  High quality case control or cohort studies with a very low risk of confounding or bias and a high probability that the relationship is causal |
| 2+ | Well conducted case control or cohort studies with a low risk of confouding or bias and a moderate probability that the relationship is causal |
| 2- | Case control or cohort studies with a high risk of confounding or bias and a significant risk that the relationship is not causal |
| 3 | Non-analytic studies, eg case reports, case series |
| 4 | Expert opinion |
| Grades of recommendation | |
| Grade | Explanation |
| A | At least one meta-analysis, systematic review, or RCT rated as 1++, and directly applicable to the target population  OR  A body of evidence consisting principally of studies rated as 1+, directly applicable to the target population, and demonstrating overall consistency of results |
| B | A body of evidence including studies rated as 2++, directly applicable to the target population, and demonstrating overall consistency of results  OR  Extrapolated evidence from studies rated as 1++ or 1+ |
| C | A body of evidence including studies rated as 2+, directly applicable to the target population and demonstrating overall consistency of results  OR  Extrapolated evidence from studies rated as 2++ |
| D | Evidence level 3 or 4  OR  Extrapolated evidence from studies rated as 2+ |
| Good practice points | Recommended best practice based on the clinical experience of the guideline development group. |

**Malaysian Society of Nephrology (MSN)**

|  | Grades of recommendation |
| --- | --- |
| A | At least one meta analysis, systematic review, or RCT, or evidence rated as good and directly apllicable to the target population. |
| B | Evidence from well conducted clinical trials, directly applicable to the target population, and demonstrating overall consistency of results; or evidence extrapolated from meta analysis, systematic review, or RCT. |
| C | Evidence from expert committee reports, or opinions and/or clinical experiences of respected authorities; inidcates absence of directly applicable clinical studies of good quality. |

**Canadian medical association journal (CMAJ)**

|  | Level of evidence |
| --- | --- |
| A | Reflects highly valid, precise and applicable studies |
| B | Reflects studies of lesser degrees of validity, including surrogate outcomes or extrapolation of study results to other populations. |
| C |  |
| D | Reflects lower level evidence and expert opinion |

[**Kidney Disease Improving Global Outcomes (KDIGO)**](https://www.theisn.org/kidney-disease-improving-global-outcomes-kdigo) **The ‘Nederlandse federatie voor Nefrologie (NfN)’ uses a grading system based on the grading system of the KDIGO**

| **Step 1:** Starting grade for quality of evidence based on study design | **Step 2**: Reduce grade | **Stap 3**: Raise grade | **Finale grade for quality of evidence and definition** |
| --- | --- | --- | --- |
| Randomized trials - **High**  Observational study - **Low**  Any other evidence – **Very low** | *Study quality:*  -1 level if serious limitation  -2 levels if very serious limitations  *Consistency:*  -1 level if important inconsistency  *Directness:*  -1 level if some uncertainty  -2 levels if major uncertainty  *Other:*  -1 level if sparse or imprecise data  -1 level if high probability of reporting bias | *Strength of association:*  +1 level is strong, no plausible confounders  +2 levels if very strong, no major threats to validity  *Other:*  +1 level if evidence of a dose-response gradient  +1 level if all residual plausible confouders would have reduced the observed effect | **High** – Further research is unlikely to change confidence in the estimate of the effect  **Moderate** – Further research is likely to have ani important impact on confidence in the estimate of effect, and may change the estimate  **Low** – Further research is verly likely to have an important impact on confidence in the estimate and may change the estimate  **Very low** – Any estimate of effect is very uncertain |

|  | Quality of evidence | Meaning |
| --- | --- | --- |
| A | High | We are confident that the true effect lies close tot hat of the estimate of the effect |
| B | Moderate | The true effect is lekely to be close to the estimate of the effect, but there is a possibility that it is substantially different. |
| C | Low | The true effect may be substantially different from the estimate of the effect. |
| D | Very low | The estimate of effect is very uncertain, and often will be far from the truth. |

**Implications of the GRADE system for patient and doctor**

|  | Patient | Doctor |
| --- | --- | --- |
| Grade 1 ‘’recommendation’’ | Most patients want the measure to be applied | Most patients can apply the measure |
| Grade 2  ‘’suggestion’’ | Most patients want the measure to be applied, but some don’t | Different choices are possible. The doctor and patient decide together if they will aplly the recommended measure. |

[**American Academy of Family Physicians**](https://www.aafp.org/home.html) **(AAFP )**

|  | Level of evidence |
| --- | --- |
| A | Consistent, good-quality patient-oriented evidence |
| B | Inconsistent or limited-quality patient-oriented evidence |
| C | Consensus, disease-oriented evidence, usual practice, expert opinion, or case series. |

**EBM practice net**

| **Level of evidence** | | **Benefits versus**  **disadvantages and**  **risks** | **Methodological**  **quality of the**  **studies** | **Implications** |
| --- | --- | --- | --- | --- |
| 1A | Strong recommendation, high level of evidence | Benefits > disadvantages and risks | RCTs without limitations or strong evidence of observational studies | Strong recommendation, can be applied to most patients and in most circumstances |
| 1B | Strong recommendation, moderate level of evidence | Benefits > disadvantages and risks | RCTs with limitations or strong evidence of observational studies | Strong recommendation, can be applied to most patients and in most circumstances |
| 1C | Strong recommendation,  low or very low level of evidence | Benefits > disadvantages and risks | Observational studies or case studies | Strong recommendation, but this can change if a higher level of evidence becomes available |
| 2A | Weak recommendation, high level of evidence | Benefits = disadvantages and risks | RCTs without limitations or strong evidence of observational studies | Weak recommendation,  the best action can  vary depending on the circumstances,  patients or  social  values |
| 2B | Weak recommendation, moderate level of evidence | Benefits = disadvantages and risks | RCTs with limitations or strong evidence of observational studies | Weak recommendation,  the best action can  vary depending on the circumstances,  patients or  social  values |
| 2C | Weak recommendation, low or very low level of evidence | Benefits = disadvantages and risks | Observational studies or case studies | Very weak recommendation, alternatives may be equally justifiable |
