# Supplementary material for: Developing quality indicators for Chronic Kidney Disease in primary care, extractable from the Electronic Medical Record. A Rand-modified Delphi method
Source: BMC Nephrol. 2020 May 5;21:161. doi: 10.1186/s12882-020-01788-8 (PMC7201612; doi:10.1186/s12882-020-01788-8)
Supplement: Supplementary file 3 — Additional file 3. Addendum 3: Questionnaire for the patients [file 12882_2020_1788_MOESM3_ESM.docx]

**Addendum 3: Letter and questionnaire patient panelmembers**

Dear Sir/Madam

First of all, we would like to thank you for your willingness to participate in our study. The Academic Centre of General Practice, a department of the University of Leuven in Belgium, is carrying out a study for the improvement of the quality of care for people with chronic kidney disease (CKD). The purpose of this study is to develop a list of medical interventions carried out by the general practictioner (called **quality indicators**). Using these quality indicators, the quality of care can be measured. All medical details of every patient are nowadays being stored in the so called Electronic Medical Record (EMR). Well, these quality indicators will be compared to the details that can be consulted directly from the EMR. The general practitioner can receive feedback afterwards: what has he/she done well, what can possibly be improved? In this way, we hope to dramatically improve the quality of care for patients with CKD.

You are invited to participate in this study due to your experience with CKD. In a first step, we will fill in a questionnaire that you can find in attachment to this document. The completion of the questionnaire will take approximately 30-40 minutes of your time. We will contact you by telefone and we will go through the questionnaire together. You can expect a telefone in the upcoming week.

In the second step, we try to reach a consensus about the acceptance, rejection or reformulation of the quality indicators, during a panel meeting in which doctors as well as patients will participate. This meeting, of which the date will be anounced later, will take approximately 2 hours of your time.

In the third and last step, we will present a written list of the final quality indicators to all the participants in order to achieve a definitive permission for publication. This step will take no longer than 15 minutes. We will contact you by telephone in the future to complete this final step.

For the time being, you don't have to undertake any actions. You can already go through the questionnaire, but wait for us to contact you to fill it in together!

If you have any questions or remarks, please do not hesitate to contact us ([steve.vandenbulck@-kuleuven.be](mailto:steve.vandenbulck@-kuleuven.be)).

Yours faithfully,

The project group existing of:

Steve Van den Bulck, GP, PhD student

Professor Patrik Vankrunkelsven, KU Leuven promotor

Professor Rosella Hermens, Radboud university, copromotor

Professor Geert Goderis, KU Leuven, copromotor

# Indicators for chronic kidney disease

**From recommendation to indicator**

For the selection of recommendations in this document, we based our search on national and international guidelines concerning the subject. The purpose for you is to score each recommendation according to its importance in measuring the quality of care for patients with chronic kidney disease.

**Instructions for completing the questionnaire**

In this questionnaire, you will find 24 recommendations, divided into the following categories: management: follow-up; management vaccination; treatment: patient education and information, lifestyle and diet, hypertension, cardiovascular disease, anemia; drugs and patient safety; referral to a specialist; role of the general practitioner and care program. The questionnaire that is sent to doctors (both GP's and specialists) is more extensive. Certain recommendations are specifically aimed towards doctors and were therefore excluded in this questionnaire. We ask you to score each recommendation according to its importance in measuring the quality of care for patients with chronic kidney disease in the first line setting. Each recommendation should be scored on a 9 point scale, with 1 being the lowest score (bad recommendation to measure the quality of care) and 9 being the highest score (excellent recommendation to measure the quality of care).

You can base your judgement of whether the recommendation is a good measure of quality of care on the following criteria:

- The recommendation is relevant in the care process of first line health care.

- The recommendation extends the (disease free) survival of the patient.

- The recommendation improves the quality of life of the patient.

- The recommendation improves the effectiveness of care for the patient (the advan tages clearly outway the costs.

Besides giving a score from 1 to 9, we also ask you to draw up a top 5 of recommendations per category, based on their adequateness for measuring the quality of care.

You have the opportunity to formulate remarks, to adjust recommendations or to write down recommendations which are not listed in the questionnaire. Please use the empty documents at the end of this questionnaire for this purpose.

On the next page, you will find an example of how the questionnaire is drawn up.

Questionnaire lay-out

| 1. ***Category*** | | | | | | |
| --- | --- | --- | --- | --- | --- | --- |
| *To what extent are the following recommendations of importance for measuring the quality of care in the first line health care for patients with chronic kidney disease?* | | | | | | |
|  | **Recommendation** | **Source** | **Year** | **Grade of evidence** | **Your judgement** | **Decisive factor in your judgement** |
|  | The recommended medical act. | .... | .... | .... | **1 2 3 4 5 6 7 8 9**  **[----------------------------------------------------------------------------]**  Poor Excellent  *□ Not judgeable* |  Importance   EMR extractability |

Here you can declare which factor was decisive in your judgement, namely the importance of the recommendation for the measurement of the quality of care, or the automatic extractability from the electronic medical record (EMR).

The abbreviation of the consulted national or international guideline.

The year of publication or last update of the guideline.

The grade of evidence as denoted in the original guideline. For a legend of each guideline: see attachment 1.

Here you can declare which factor was decisive in your judgement, namely the importance of the recommendation for the measurement of the quality of care, or the automatic extractability from the electronic medical record (EMR).

Score of 1-9. If you deem yourself uncapable of judging the recommendation, you have the possibility to tick off the box with "not judgeable".

**Sources**

Below you will find a list of the guidelines we consulted to make up the list of recommendations as presented in this questionnaire. Also noted are the abbreviations as we used in the questionnaire (between brackets), the year of publication or the last update of the guideline and the country of origin.

- American College of Physicians (ACP)
  - American Academy for Family Physicians (AAFP): Verenigde Staten
    - Chronic Kidney Disease: Detection and Evaluation. 2011
    - Update on the Management of Chronic Kidney Disease. 2012
    - ACP Releases Guideline on Screening, Monitoring, and Treatment of Stage 1 to 3 Chronic Kidney Disease. 2014
  - Annals of Internal Medicine
    - Screening, Monitoring, and Treatment of Stage 1 to 3 Chronic Kidney Disease: A Clinical Practice Guideline From the American College of Physicians. 2013
- Canadian Medical Association Journal (CMAJ): Guidelines for the management of chronic kidney disease. 2008; Canada
- Domus medica (DM): Chronische nierinsufficiëntie. 2012; België
- Evidence Based Medicine Practice Net (EBM): Multidisciplinaire richtlijn Chronisch nierlijden (CNI) - Aanvulling op de richtlijn ‘Chronische nierinsufficiëntie’ van Domus Medica, 2012. 2017; België
- Kidney Disease Improving Global Outcomes (KDIGO): Clinical Practice Guideline for the Evaluation and Management of Chronic Kidney Disease. 2012; Verenigde Staten
- Malaysian Society of Nephrology (MSN) Management of Chronic Kidney Disease in Adults. Clinical Practice Guidelines. 2011; Maleisië
- National Institute for Health and Care Excellence (NICE); Verenigd Koninkrijk
  - Clinical Guideline (CG) 182: Chronic kidney disease in adults: assessment and management. 2015 update
- Nederlandse Federatie voor Nefrologie (NFN): Diagnostiek en Behandeling van Patiënten met Chronische Nierschade;  2015 herziening; Nederland
- Scottish Intercollegiate Guidelines Network (SIGN) 103: Diagnosis and management of chronic kidney disease. 2008; Schotland

For an explanation about the grade of evidence used by each guideline, see attachment 1.

# Informed consent

Title of the study: The electronic medical record and the quality of care chronic kidney disease.

Institution: Academic Centre for General Practice, University of Leuven, Leuven (Belgium)

Project group: Steve Van den Bulck, GP, PhD student

Professor Patrik Vankrunkelsven, KU Leuven promotor

Professor Rosella Hermens, Radboud university, copromotor

Professor Geert Goderis, KU Leuven, copromotor

I hearby declare to be informed in a comprehensible way about the nature, the method and the purpose of this study. My questions were answered to my satisfaction.

I agree voluntary to the participation in this study.

I am informed about the fact that participation in this study doesn't bring with it any additional costs and that there is no financial advantage to be gained.

I preserve the right to withdraw my consent of participation in this study, without any declaration and without being of any influence to my personal or professional life.

My personal details cannot be obtained by a third person without my explicit permission.

If I wish to obtain more information about the study, now or in the future, I can apply to Steve Van den Bulck ([steve.vandenbulck@kuleuven.be](mailto:steve.vandenbulck@kuleuven.be)).

Read and approved,

Name of the participant: ………………………………………………………………………………

Date: ………………………………………………………………………………

Signature: ……………………………………………………………………………….

# **Front page**

Name......................:............................................................................................................................

Age:........... Sex:...........

City/town of recidence...................:....................................................................................................

Years since diagnosis of chronic kidney disease:.............

**Questionnaire chronic kidney disease**

| 1. ***Management: follow-up*** | | | | | |
| --- | --- | --- | --- | --- | --- |
| 1 | Identify the **rate of progression of CKD:** Obtain a minimum of 3 GFR estimations over a period of not less than 90 days. | NICE CG182  DM | 2014 (2015 update)  2012 | None  Consensus | **1 2 3 4 5 6 7 8 9**  **[---------------------------------------------------------]**  Poor Excellent *□Not judgeable*  **Rating based on:** *□ EMR extractability □ Relevance recommendation* |
| 2 | Identify the **rate of progression of CKD** in people with a new finding of reduced GFR, repeat the GFR within 2 weeks to exclude causes of acute deterioration of GFR – for example, acute kidney injury or starting [renin–angiotensin system antagonist](http://www.nice.org.uk/guidance/cg182/chapter/recommendations#terms-used-in-this-guideline) therapy | NICE CG182 | 2014 (2015 update) | None | **1 2 3 4 5 6 7 8 9**  **[---------------------------------------------------------]**  Poor Excellent *□Not judgeable*  **Rating based on:** *□ EMR extractability □ Relevance recommendation* |
| 3 | Use the following table to guide the frequency of GFR monitoring for people with, or at risk of CKD:   \| **Stage** \| **GFR** \| **Test** \| **Frequency** \| \| --- \| --- \| --- \| --- \| \| 1 and 2 \| >60 \| eGFR \| Annually \| \| 3A \| 45-59 \| eGFR \| Every six months \| \| 3B \| 30-44 \| eGFR \| Every six months \| \| 4 \| 15-29 \| eGFR \| Min. Every tree months \|  - Tailor the frequency depending on the presence of other risk factors on the development of terminal renal failure and the progression of the eGFR - Let the frequency of detecting proteinuria and complications suspend upon the eGFR, the progression of the eGFR and drug therapy | DM | 2012 | Consensus | **1 2 3 4 5 6 7 8 9**  **[---------------------------------------------------------]**  Poor Excellent *□Not judgeable*  **Rating based on:** *□ EMR extractability □ Relevance recommendation* |
| 4 | Metabolic complications of kidney failure: Measure serum potassium, calcium, phosphate, PTH and bicarbonate levels, and Hb in patiënts with CKD with a moderate (code orange) to strongly (code red) increased risk. In case of increased PTH, also measure vitamin D, and in the case of reduced Hb also measure ferritin and transferrin saturation. The frequency of these measurements depends on the degree of kidney damage. | NfN | 2015 | none | **1 2 3 4 5 6 7 8 9**  **[---------------------------------------------------------]**  Poor Excellent *□Not judgeable* **Rating based on:** *□ EMR extractability □ Relevance recommendation* |

**Top 3 recommendation**

Which recommendations for ‘managment: follow-up’ of chronic renal insufficiency do you find most suitable for measuring the quality of care?

| Top 3 recommendations regarding managment: follow-up | | |
| --- | --- | --- |
| Position | Number of recommendation | Motivation |
| 1st position |  |  |
| 2nd position |  |  |
| 3rd position |  |  |

| 1. ***Management: Vaccination*** | | | | | |
| --- | --- | --- | --- | --- | --- |
| 5 | We recommend that all adults with CKD are offered annual **vaccination** with **influenza** vaccine, unless contraindicated. | KDIGO NfN | 2012 2015 | 1B 1B | **1 2 3 4 5 6 7 8 9**  **[---------------------------------------------------------]**  Poor Excellent *□Not judgeable*  **Rating based on:** *□ EMR extractability □ Relevance recommendation* |
| 6 | We recommend that all adults with eGFR < 30 ml/min/1.73 m2 (GFR categories G4-G5) and those at high risk of **pneumococcal infection** (e.g., nephrotic syndrome, diabetes, or those receiving immunosuppression) receive vaccination with polyvalent pneumococcal vaccine unless contraindicated. | KDIGO | 2012 | 1B | **1 2 3 4 5 6 7 8 9**  **[---------------------------------------------------------]**  Poor Excellent *□Not judgeable* **Rating based on:** *□ EMR extractability □ Relevance recommendation* |
| 7 | We recommend that all adults who are at high risk of progression of CKD and have GFR < 30 ml/min/1.73 m2 (GFR categories G4-G5) be immunized against **hepatitis B** and the response confirmed by appropriate serological testing. (1B) | NfN KDIGO | 2015 2012 | 1B 1B | **1 2 3 4 5 6 7 8 9**  **[---------------------------------------------------------]**  Poor Excellent *□Not judgeable*  **Rating based on:** *□ EMR extractability □ Relevance recommendation* |

**Top recommendation**

Which recommendation for vaccination of chronic renal insufficiency do you find most suitable for measuring the quality of care?

| Top recommendation regarding vaccination. | | |
| --- | --- | --- |
| Position | Number of recommendation | Motivation |
| 1st position |  |  |

| 1. ***Treatment of CKD*** | | | | | |
| --- | --- | --- | --- | --- | --- |
| **3.1. Treatment: Patient education and information** | | | | | |
| 8 | Offer tailored education and support programs in the self-management of CKD patients (GPP). Referral to a specialized nurse is recommended in order to ameliorate understanding of their condition, to ameliorate compliance to lifestyle changes and drug treatment (GRADE 1C). These measures are taken to stabilize parameters and to preserve renal function as long as possible (GPP). | EBM | 2017 | GPP 1C GPP | **1 2 3 4 5 6 7 8 9**  **[---------------------------------------------------------]**  Poor Excellent *□Not judgeable* **Rating based on:** *□ EMR extractability □ Relevance recommendation* |
| 9 | Provide the following support: information for patients with CKD about their condition, a program for 'shared decision making', support for self-management (eg blood pressure, smoking cessation, exercise, diet and medication) and support in making a well informed choice. | EBM | 2017 | 2B | **1 2 3 4 5 6 7 8 9**  **[---------------------------------------------------------]**  Poor Excellent *□Not judgeable*  **Rating based on:** *□ EMR extractability □ Relevance recommendation* |
| **3.2. Treatment: Lifestyle and diet** | | | | | |
| 10 | Patients with CKD should be encouraged to (GRADE 1B):   - Undertake physical activity compatible with cardiovascular health and tolerance (aiming for at least 30 minutes 5 times per week) - Stop smoking - Obtain or maintain a healthy weight   - (BMI 20 to 25, depending on country-specific demographics).   - Waist circumference:     - Waist circumference ≥94 cm in men or ≥80 cm in women (SIGN 103: GPP)     - Waist circumference < 102 cm for men, < 88 cm for women (CMAJ 2008: D) - Limit the alcohol intake (only mentioned in NfN, CMAJ) | EBM NICE CG182  SIGN 103 KDIGO NfN MSN DM  CMAJ | 2017 2014 (2015 update) 2008  2012 2015 2011 2012  2008 | 1B None  GPP  1D 1D B 1B-1C (roken)-1B D-D-D-B | **1 2 3 4 5 6 7 8 9**  **[---------------------------------------------------------]**  Poor Excellent *□Not judgeable*  **Rating based on:** *□ EMR extractability □ Relevance recommendation* |
| 11 | For CNI stage 1 to 3, no specific dietary advice other than the healthy diet recommended to the general population is required, except for patients with hypertension (low salt) or hypercholesterolemia (low saturated fatty acids). | EBM | 2017 | GPP | **1 2 3 4 5 6 7 8 9**  **[---------------------------------------------------------]**  Poor Excellent *□Not judgeable*  **Rating based on:** *□ EMR extractability □ Relevance recommendation* |
| 12 | Individuals with CKD at high risk should receive expert dietary advice and information in the context of an education program, tailored to severity of CKD and the need to intervene on salt, phosphate, potassium, and protein intake where indicated. | NfN  KDIGO  EBM | 2015  2012  2017 | 1B  1B  1B | **1 2 3 4 5 6 7 8 9**  **[---------------------------------------------------------]**  Poor Excellent *□Not judgeable*  **Rating based on:** *□ EMR extractability □ Relevance recommendation* |
| 13 | Complex diets for some patients with severe CKD require specialized guidance by a dietician to prevent dietary errors and/or malnutrition. Dietary advice about potassium and phosphate intake tailored to CKD stage is made by an appropriately qualified **dietitian**. | EBM | 2017 | 1B | **1 2 3 4 5 6 7 8 9**  **[---------------------------------------------------------]**  Poor Excellent *□Not judgeable* **Rating based on:** *□ EMR extractability □ Relevance recommendation* |
| **3.3. Treatment of hypertension** | | | | | |
| 14 | Target BP should be **<130/80** (SBP range 120 - 129) mmHg   - In patients with proteinuria ≥1 g/day. (MSN, SIGN Grade A) - In patients with normal urinary albumin concentrations (AAFP Grade B) - In patients with diabetes. (MSN Grade B, CMAJ grade B) - In patients with a strongly increased albuminuria, first choice antihypertensive drugs to achieve these goals are ACE inhibitors or ARB's (NfN grade 1B, KDIGO grade 2D) - In people with **ACR**  **≥70** mg/mmol (NICE CG182, none) | MSN SIGN AAFP CMAJ NfN KDIGONICE CG182 | 2011 2008 2005 2008 2015 2012 2014 (2015 upd) | A A  B B | **1 2 3 4 5 6 7 8 9**  **[---------------------------------------------------------]**  Poor Excellent *□Not judgeable* **Rating based on:** *□ EMR extractability □ Relevance recommendation* |
| 15 | We recommend that in both diabetic and non-diabetic adults with CKD and urine albumin excretion <30 mg/ 24 hours (or <3mg/mmol) whose office BP is consistently >140 mm Hg systolic or >90 mm Hg diastolic be treated with BP-lowering drugs to maintain a BP that is consistently **≤**140 mm Hg systolic and **≤**90 mm Hg diastolic. (1B) | NfN KDIGO NICE CG182  MSN DM | 2015 2012 2014 (2015 update) 2011 2012 | 1B 1B None  A 1B | **1 2 3 4 5 6 7 8 9**  **[---------------------------------------------------------]**  Poor Excellent *□Not judgeable*  **Rating based on:** *□ EMR extractability □ Relevance recommendation* |
| **3.4. Treatment of cardiovascular disease** | | | | | |
| 16 | We suggest that adults with CKD at risk for **atherosclerotic events** be offered treatment with **antiplatelet agents** unless there is an increased bleeding risk that needs to be balanced against the possible cardiovascular benefits. | KDIGO | 2012 | 2B | **1 2 3 4 5 6 7 8 9**  **[---------------------------------------------------------]**  Poor Excellent *□Not judgeable* **Rating based on:** *□ EMR extractability □ Relevance recommendation* |
| 17 | In people with **CKD and heart failure**, any escalation in therapy and/or clinical deterioration should promptmonitoring of eGFR and serum potassium concentration. | KDIGO | 2012 | None | **1 2 3 4 5 6 7 8 9**  **[---------------------------------------------------------]**  Poor Excellent *□Not judgeable*  **Rating based on:** *□ EMR extractability □ Relevance recommendation* |
| **3.5. Treatment anemia** | | | | | |
| 18 | If not already measured, check the haemoglobin level in people with a GFR < 45 ml/min/1.73 m^2^ (GFR category G3b, G4 or G5) to identify anaemia (haemoglobin less than 110 g/litre [11.0 g/dl]). Determine the subsequent frequency of testing by the measured value and the clinical circumstances. | NICE CG182  DM | 2014 (2015 update) 2012 | None  1C | **1 2 3 4 5 6 7 8 9**  **[---------------------------------------------------------]**  Poor Excellent *□Not judgeable* **Rating based on:** *□ EMR extractability □ Relevance recommendation* |

**Top 5 recommendations**

Which recommendations for the treatment of chronic renal insufficiency do you find most suitable for measuring the quality of care?

| Top 5 recommendations regarding treatment. | | |
| --- | --- | --- |
| Position | Number of recommendation | Motivation |
| 1st position |  |  |
| 2nd position |  |  |
| 3rd position |  |  |
| 4th position |  |  |
| 5th position |  |  |

| 1. ***Medication and patient safety*** | | | | | |
| --- | --- | --- | --- | --- | --- |
| 19 | We recommend not using fytotherapy in people with CKD. | EBM | 2017 | 1B | **1 2 3 4 5 6 7 8 9**  **[---------------------------------------------------------]**  Poor Excellent *□Not judgeable*  **Rating based on:** *□ EMR extractability □ Relevance recommendation* |

**Top recommendation**

Which recommendations for medication and safety of the patient of chronic renal insufficiency do you find most suitable for measuring the quality of care?

| Top 5 recommendations regarding medication and safety of the patient. | | |
| --- | --- | --- |
| Position | Number of recommendation | Motivation |
| 1st position |  |  |

| 1. ***Referral to specialist*** | | | | | |
| --- | --- | --- | --- | --- | --- |
| **5.1 For patients with CKD, referral to a nephrologist or specialist with specific knowledge of CKD is advised in the following cases:** | | | | | |
| 20.1 | Acute kidney injury or abrupt sustained fall in GFR. | NfN, KDIGO | 2015 2012 | 1B 1B | **1 2 3 4 5 6 7 8 9**  **[---------------------------------------------------------]**  Poor Excellent *□Not judgeable*  **Rating based on:** *□ EMR extractability □ Relevance recommendation* |
| 20.2 | GFR <30 ml/min/1,73 m2 (GFR categorie G4 or G5). | NfN, KDIGOAAFP AAFP NICE CG 182  MSN DM | 2015 2012 2004 (I) 2011 2014 (2015 update) 2011 2012 | 1B 1B C C none   C 2B | **1 2 3 4 5 6 7 8 9**  **[---------------------------------------------------------]**  Poor Excellent *□Not judgeable* **Rating based on:** *□ EMR extractability □ Relevance recommendation* |
| 20.3 | Patients <75 jaar with an eGFR between 30 and 45 ml/min./1,73 m² and an ACR of 20-200mg/g for males and 30-300 mg/g for females. | EBM  DM | 2017  2012 | 2B  2B | **1 2 3 4 5 6 7 8 9**  **[---------------------------------------------------------]**  Poor Excellent *□Not judgeable*  **Rating based on:** *□ EMR extractability □ Relevance recommendation* |
| 20.4 | Patients with an eGFR >45 ml/min./1,73 m² and an ACR >200 mg/g for males or 300 mg/g for females and/or a PCR >1 000 mg/g. | EBM  DM | 2017  2012 | 2B  2B | **1 2 3 4 5 6 7 8 9**  **[---------------------------------------------------------]**  Poor Excellent *□Not judgeable*  **Rating based on:** *□ EMR extractability □ Relevance recommendation* |
| 20.5 | A consistent finding of significant albuminuria (ACR ≥300 mg/g [≥30 mg/mmol] or AER ≥ 300 mg/ 24 hours, approximately equivalent to PCR ≥ 500 mg/g [≥50 mg/mmol] or PER ≥500 mg/24 hours | NfN, KDIGO, EBM | 2015 2012 2017 | 1B 1B 1B | **1 2 3 4 5 6 7 8 9**  **[---------------------------------------------------------]**  Poor Excellent *□Not judgeable*  **Rating based on:** *□ EMR extractability □ Relevance recommendation* |
| 20.6 | Heavy proteinuria (urine protein ≥1 g/day or urine protein: creatinine ratio (uPCR) ≥0.1 g/mmol) unless known to be due to diabetes and optimally treated | MSN | 2011 | C | **1 2 3 4 5 6 7 8 9**  **[---------------------------------------------------------]**  Poor Excellent *□Not judgeable*  **Rating based on:** *□ EMR extractability □ Relevance recommendation* |
| 20.7 | Haematuria with proteinuria (urine protein ≥0.5 g/day or uPCR ≥0.05 g/mmol) | MSN | 2011 | C | **1 2 3 4 5 6 7 8 9**  **[---------------------------------------------------------]**  Poor Excellent *□Not judgeable*  **Rating based on:** *□ EMR extractability □ Relevance recommendation* |
| 20.8 | ACR ≥ 70 mg/mmol, unless known to be caused by diabetes and already appropriately treated | NICE CG182 | 2014 (update 2015) | None | **1 2 3 4 5 6 7 8 9**  **[---------------------------------------------------------]**  Poor Excellent *□Not judgeable*  **Rating based on:** *□ EMR extractability □ Relevance recommendation* |
| 20.9 | ACR ≥ 30 mg/mmol or more (ACR category A3), together with haematuria | NICE CG182 | 2014 (update 2015) | None | **1 2 3 4 5 6 7 8 9**  **[---------------------------------------------------------]**  Poor Excellent *□Not judgeable*  **Rating based on:** *□ EMR extractability □ Relevance recommendation* |
| 20.10 | Progression of CKD: confirmed decline in GFR category accompanied by a 25% or greater drop in eGFR from baseline or a sustained decline in eGFR of more than 5 ml/min/1.73 m2 /year. | NfN, KDIGO | 2015 2012 | 1B 1B | **1 2 3 4 5 6 7 8 9**  **[---------------------------------------------------------]**  Poor Excellent *□Not judgeable*  **Rating based on:** *□ EMR extractability □ Relevance recommendation* |
| 20.11 | Patients with progressive CKD, being an eGFR between 30 en 45 ml/min./1,73 m² and a decline of eGFR >10 ml/min in five years time or > 5 ml/min in two years time. | EBM  DM | 2017  2012 | 2B  2B | **1 2 3 4 5 6 7 8 9**  **[---------------------------------------------------------]**  Poor Excellent *□Not judgeable*  **Rating based on:** *□ EMR extractability □ Relevance recommendation* |
| 20.12 | Rapidly declining renal function (loss of glomerular filtration rate/GFR >5 ml/min/1.73m2 in one year or >10 ml/min/1.73m2 Within five years) | MSN | 2011 | C | **1 2 3 4 5 6 7 8 9**  **[---------------------------------------------------------]**  Poor Excellent *□Not judgeable*  **Rating based on:** *□ EMR extractability □ Relevance recommendation* |
| 20.13 | Sustained decrease in GFR of 25% or more, and a change in GFR category or sustained decrease in GFR of 15 ml/min/1.73 m^2^ or more within 12 months | NICE CG182 | 2014 (update 2015) | none | **1 2 3 4 5 6 7 8 9**  **[---------------------------------------------------------]**  Poor Excellent *□Not judgeable*  **Rating based on:** *□ EMR extractability □ Relevance recommendation* |
| 20.14 | Dysmorphic  erytrocytes in urine sedimentation (>20 pgv). | NfN, KDIGO | 2015,2012 | 1B,1B | **1 2 3 4 5 6 7 8 9**  **[---------------------------------------------------------]**  Poor Excellent *□Not judgeable*  **Rating based on:** *□ EMR extractability □ Relevance recommendation* |
| 20.15 | Patients with hematuria of unknown origin (GRADE 1B); | EBM | 2017 | 1B | **1 2 3 4 5 6 7 8 9**  **[---------------------------------------------------------]**  Poor Excellent *□Not judgeable* **Rating based on:** *□ EMR extractability □ Relevance recommendation* |
| 20.16 | CKD combined with hypertension, which insufficiëntly responds to medical treatment with 4 or more antihypertensive drugs. | NfN KDIGO EBM MSN NICE CG182 | 2015 2012 2017 2011 2014 (update 2015) | 1B 1B 1B C none | **1 2 3 4 5 6 7 8 9**  **[---------------------------------------------------------]**  Poor Excellent *□Not judgeable*  **Rating based on:** *□ EMR extractability □ Relevance recommendation* |
| 20.17 | Persisting serum potassium abnormalities. | NfN KDIGO EBM | 2015 2012 2017 | 1B 1B 1B | **1 2 3 4 5 6 7 8 9**  **[---------------------------------------------------------]**  Poor Excellent *□Not judgeable*  **Rating based on:** *□ EMR extractability □ Relevance recommendation* |
| 20.18 | Recurrent or extensive nefrolithiasis. | NfN KDIGO EBM | 2015 2012 2017 | 1B 1B 1B | **1 2 3 4 5 6 7 8 9**  **[---------------------------------------------------------]**  Poor Excellent *□Not judgeable*  **Rating based on:** *□ EMR extractability □ Relevance recommendation* |
| 20.19 | Hereditary kidney disease. | NfN KDIGO EBM MSN NICE CG182 | 2012 2015 2017 2011 2014 (update 2015) | 1B 1B 1B C none | **1 2 3 4 5 6 7 8 9**  **[---------------------------------------------------------]**  Poor Excellent *□Not judgeable*  **Rating based on:** *□ EMR extractability □ Relevance recommendation* |
| 20.20 | When a.renalis stenosis is suspected or established. | MSN EBM | 2011 2017 | C GPP | **1 2 3 4 5 6 7 8 9**  **[---------------------------------------------------------]**  Poor Excellent *□Not judgeable* **Rating based on:** *□ EMR extractability □ Relevance recommendation* |
| 20.21 | For treatment of complications of kidney failure: anemia, electrolyte imbalance, vitamin D, calcium- and phosphate disturbances, uremic complaints (pruritus,…). | EBM | 2017 | GPP | **1 2 3 4 5 6 7 8 9**  **[---------------------------------------------------------]**  Poor Excellent *□Not judgeable*  **Rating based on:** *□ EMR extractability □ Relevance recommendation* |
| 20.22 | Suspected glomerular disease. | MSN NICE CG182 | 2011 2014 (update 2015) | C none | **1 2 3 4 5 6 7 8 9**  **[---------------------------------------------------------]**  Poor Excellent *□Not judgeable*  **Rating based on:** *□ EMR extractability □ Relevance recommendation* |
| 20.23 | Pregnant or when pregnancy is planned. | MSN | 2011 | C | **1 2 3 4 5 6 7 8 9**  **[---------------------------------------------------------]**  Poor Excellent *□Not judgeable*  **Rating based on:** *□ EMR extractability □ Relevance recommendation* |
| 20.24 | Unclear cause of CKD. | MSN | 2011 | C | **1 2 3 4 5 6 7 8 9**  **[---------------------------------------------------------]**  Poor Excellent *□Not judgeable*  **Rating based on:** *□ EMR extractability □ Relevance recommendation* |
| 21 | Refer high risk patiënts for inclusion in the care program. These are patients with:   - a chronic eGFR <30 ml/min./1,73 m² (eGFR categories G4-G5) (GRADE 1B); - an eGFR between 30-45 ml/min./1,73 m² and ACR >200 mg/g for males or 300 mg/g for females, and/or proteïnuria >1000 mg/24h or a protein-creatininratio (PCR) >1 000 mg/g (GRADE 2B). | EBM | 2017 | 1B-2B | **1 2 3 4 5 6 7 8 9**  **[---------------------------------------------------------]**  Poor Excellent *□Not judgeable* **Rating based on:** *□ EMR extractability □ Relevance recommendation* |
| **5.2 Renal replacement therapy** | | | | | |
| 22 | We recommend timely referral for planning renal replacement therapy (RRT) in people with progressive CKD in whom the risk of kidney failure within 1 year is 10–20% or higher , as determined by validated risk prediction tools. | EBM  KDIGO | 2017  2012 | 1B  1B | **1 2 3 4 5 6 7 8 9**  **[---------------------------------------------------------]**  Poor Excellent *□Not judgeable*  **Rating based on:** *□ EMR extractability □ Relevance recommendation* |

**Top 5 recommendation**

Which recommendations for refferal to the specialist of chronic renal insufficiency do you find most suitable for measuring the quality of care?

| Top 5 recommendation regarding refferal to the specialist. | | |
| --- | --- | --- |
| Position | Number of recommendation | Motivation |
| 1st position |  |  |
| 2nd position |  |  |
| 3rd position |  |  |
| 4th position |  |  |
| 5th position |  |  |

| 1. ***Role of the general practitioner and care program*** | | | | | |
| --- | --- | --- | --- | --- | --- |
| 23 | We suggest that people with progressive CKD should be managed in a multidisciplinary care setting. (2B) The multidisciplinary team should include or have access to dietary counseling, education and counseling about different RRT modalities, transplant options, vascular access surgery, and ethical, psychological, and social care. | EBM KDIGO | 2017 2012 | 2B – GPP None | **1 2 3 4 5 6 7 8 9**  **[---------------------------------------------------------]**  Poor Excellent *□Not judgeable*  **Rating based on:** *□ EMR extractability □ Relevance recommendation* |
| 24 | The comprehensive conservative management program should include protocols for symptom and pain management, psychological care, spiritual care, and culturally sensitive care for the dying patient and their family (whether at home, in a hospice or a hospital setting), followed by the provision of culturally appropriate bereavement support. | EBM  KDIGO  CMAJ | 2017  2012  2008 | GPP  None  D | **1 2 3 4 5 6 7 8 9**  **[---------------------------------------------------------]**  Poor Excellent *□Not judgeable*  **Rating based on:** *□ EMR extractability □ Relevance recommendation* |

**Top recommendation**

Which recommendations for the role of the general practitioner and care program of chronic renal insufficiency do you find most suitable for measuring the quality of care?

| Top recommendation regarding the role of the general practitioner and care program. | | |
| --- | --- | --- |
| Position | Number of recommendation | Motivation |
| 1st position |  |  |

**Suggestions**

If you have any suggestions for recommendations which you think are missing in the foregoing list, please write them down in this table.

| Position | Recommendation | Motivation |
| --- | --- | --- |
| 1) |  |  |
| 2) |  |  |
| 3) |  |  |
| 4) |  |  |
| 5) |  |  |
| 6) |  |  |
| 7) |  |  |
| 8) |  |  |
| 9) |  |  |
| 10) |  |  |

**Appendix 1: Explanation of the grading systems**

This appendix explains the different grading systems used in the guidelines. The National Institute for Health and Care Excellence (NICE) don’t use a grading system.

**Domus Medica**

The level of evidence is represented by the letters A, B and C; A has the highest level of evidence, C the lowest. The numbers 1 and 2 are associated with the letters; 1 has a high and 2 a low grade of recommendation.

|  | Level of evidence | Benefits versus disadvantages |
| --- | --- | --- |
| 1A | Strong recommendation  High level of evidence | Benefits > disadvantages and risks |
| 1B | Moderate level of evidence |  |
| 1C | Low level of evidence |  |
| 2A | Weak recommendation, high level of evidence | Benefits = disadvantages (in balance) |
| 2B | Weak recommendation, moderate level of evidence |  |
| 2C | Weak recommendation, low level of evidence | Balance or uncertainty about benefits and disadvantages. |
| GPP | "Good Practice Point"  (consensus) |  |

**Scottish Intercollegiate Guidelines Network (SIGN)**

The authors of the SIGN guideline divide the used sources according to the level of evidence. They use the level of evidence to assign a certain degree to each recommendation.

| Levels of evidence | |
| --- | --- |
| Level | Explanation |
| 1++ | High quality meta-analyses, systematic reviews or RCTs, or RCTs with a very low risk of bias |
| 1+ | Well conducted meta-analyses, systematic reviews, or RCTs with a low risk of bias |
| 1- | Meta-analyses, systematic reviews, or RCTs with a high risk of bias |
| 2++ | High quality systematic reviews of case control or cohort studies  High quality case control or cohort studies with a very low risk of confounding or bias and a high probability that the relationship is causal |
| 2+ | Well conducted case control or cohort studies with a low risk of confouding or bias and a moderate probability that the relationship is causal |
| 2- | Case control or cohort studies with a high risk of confounding or bias and a significant risk that the relationship is not causal |
| 3 | Non-analytic studies, eg case reports, case series |
| 4 | Expert opinion |
| Grades of recommendation | |
| Grade | Explanation |
| A | At least one meta-analysis, systematic review, or RCT rated as 1++, and directly applicable to the target population  OR  A body of evidence consisting principally of studies rated as 1+, directly applicable to the target population, and demonstrating overall consistency of results |
| B | A body of evidence including studies rated as 2++, directly applicable to the target population, and demonstrating overall consistency of results  OR  Extrapolated evidence from studies rated as 1++ or 1+ |
| C | A body of evidence including studies rated as 2+, directly applicable to the target population and demonstrating overall consistency of results  OR  Extrapolated evidence from studies rated as 2++ |
| D | Evidence level 3 or 4  OR  Extrapolated evidence from studies rated as 2+ |
| Good practice points | Recommended best practice based on the clinical experience of the guideline development group. |

**Malaysian Society of Nephrology (MSN)**

|  | Grades of recommendation |
| --- | --- |
| A | At least one meta analysis, systematic review, or RCT, or evidence rated as good and directly apllicable to the target population. |
| B | Evidence from well conducted clinical trials, directly applicable to the target population, and demonstrating overall consistency of results; or evidence extrapolated from meta analysis, systematic review, or RCT. |
| C | Evidence from expert committee reports, or opinions and/or clinical experiences of respected authorities; inidcates absence of directly applicable clinical studies of good quality. |

**Canadian medical association journal (CMAJ)**

|  | Level of evidence |
| --- | --- |
| A | Reflects highly valid, precise and applicable studies |
| B | Reflects studies of lesser degrees of validity, including surrogate outcomes or extrapolation of study results to other populations. |
| C |  |
| D | Reflects lower level evidence and expert opinion |

[**Kidney Disease Improving Global Outcomes (KDIGO)**](https://www.theisn.org/kidney-disease-improving-global-outcomes-kdigo) **The ‘Nederlandse federatie voor Nefrologie (NfN)’ uses a grading system based on the grading system of the KDIGO**

| **Step 1:** Starting grade for quality of evidence based on study design | **Step 2**: Reduce grade | **Stap 3**: Raise grade | **Finale grade for quality of evidence and definition** |
| --- | --- | --- | --- |
| Randomized trials - **High**  Observational study - **Low**  Any other evidence – **Very low** | *Study quality:*  -1 level if serious limitation  -2 levels if very serious limitations  *Consistency:*  -1 level if important inconsistency  *Directness:*  -1 level if some uncertainty  -2 levels if major uncertainty  *Other:*  -1 level if sparse or imprecise data  -1 level if high probability of reporting bias | *Strength of association:*  +1 level is strong, no plausible confounders  +2 levels if very strong, no major threats to validity  *Other:*  +1 level if evidence of a dose-response gradient  +1 level if all residual plausible confouders would have reduced the observed effect | **High** – Further research is unlikely to change confidence in the estimate of the effect  **Moderate** – Further research is likely to have ani important impact on confidence in the estimate of effect, and may change the estimate  **Low** – Further research is verly likely to have an important impact on confidence in the estimate and may change the estimate  **Very low** – Any estimate of effect is very uncertain |

|  | Quality of evidence | Meaning |
| --- | --- | --- |
| A | High | We are confident that the true effect lies close tot hat of the estimate of the effect |
| B | Moderate | The true effect is lekely to be close to the estimate of the effect, but there is a possibility that it is substantially different. |
| C | Low | The true effect may be substantially different from the estimate of the effect. |
| D | Very low | The estimate of effect is very uncertain, and often will be far from the truth. |

**Implications of the GRADE system for patient and doctor**

|  | Patient | Doctor |
| --- | --- | --- |
| Grade 1 ‘’recommendation’’ | Most patients want the measure to be applied | Most patients can apply the measure |
| Grade 2  ‘’suggestion’’ | Most patients want the measure to be applied, but some don’t | Different choices are possible. The doctor and patient decide together if they will aplly the recommended measure. |

[**American Academy of Family Physicians**](https://www.aafp.org/home.html) **(AAFP)**

|  | Level of evidence |
| --- | --- |
| A | Consistent, good-quality patient-oriented evidence |
| B | Inconsistent or limited-quality patient-oriented evidence |
| C | Consensus, disease-oriented evidence, usual practice, expert opinion, or case series. |

**EBM practice net**

| **Level of evidence** | | **Benefits versus**  **disadvantages and**  **risks** | **Methodological**  **quality of the**  **studies** | **Implications** |
| --- | --- | --- | --- | --- |
| 1A | Strong recommendation, high level of evidence | Benefits > disadvantages and risks | RCTs without limitations or strong evidence of observational studies | Strong recommendation, can be applied to most patients and in most circumstances |
| 1B | Strong recommendation, moderate level of evidence | Benefits > disadvantages and risks | RCTs with limitations or strong evidence of observational studies | Strong recommendation, can be applied to most patients and in most circumstances |
| 1C | Strong recommendation,  low or very low level of evidence | Benefits > disadvantages and risks | Observational studies or case studies | Strong recommendation, but this can change if a higher level of evidence becomes available |
| 2A | Weak recommendation, high level of evidence | Benefits = disadvantages and risks | RCTs without limitations or strong evidence of observational studies | Weak recommendation,  the best action can  vary depending on the circumstances,  patients or  social  values |
| 2B | Weak recommendation, moderate level of evidence | Benefits = disadvantages and risks | RCTs with limitations or strong evidence of observational studies | Weak recommendation,  the best action can  vary depending on the circumstances,  patients or  social  values |
| 2C | Weak recommendation, low or very low level of evidence | Benefits = disadvantages and risks | Observational studies or case studies | Very weak recommendation, alternatives may be equally justifiable |
