# Supplementary material for: Developing quality indicators for Chronic Kidney Disease in primary care, extractable from the Electronic Medical Record. A Rand-modified Delphi method
Source: BMC Nephrol. 2020 May 5;21:161. doi: 10.1186/s12882-020-01788-8 (PMC7201612; doi:10.1186/s12882-020-01788-8)
Supplement: Supplementary file 4 — Additional file 4. Addendum 4: Table of recommendations switched from category [file 12882_2020_1788_MOESM4_ESM.docx]

**Addendum 4: Tables of post-panel recommendations in-or exclusion category switching**

**4.1. Recommendations kept in ‘selection’ category
4.2. Recommendations switched from ‘discussion’ to ‘selection’ category
4.3. Recommendations switched from ‘no selection’ to ‘selection’ category
4.4. Recommendations switched from ‘selection’ to ‘no selection’ category
4.5. Recommendations switched from ‘discussion’ to ‘no selection’ category**

| **4.1. Recommendations kept in ‘selection’ category** | |
| --- | --- |
| Nr | Description |
| 1 | In people with GFR < 60 ml/min/1.73 m^2^ (GFR categories G3a-G5) or markers of kidney damage, review past history and previous measurements to determine duration of kidney disease.   - If duration is >3 months, CKD is confirmed. Follow recommendations for CKD. - If duration is not >3 months or unclear, CKD is not confirmed. Patients may have CKD or acute kidney diseases (including AKI) or both and tests should be repeated accordingly. |
| 2 | Classification of chronic kidney disease (CKD) should be based on the existing NKF-KDOQI* staging (refer to Table 3). (the KDOQI exists out of 6 GFR categories and 3 albuminuria categories) |
| 6 | Confirm a positive test strip (1+ or more) with a quantitative measurement and express it as a ratio to creatinine (ACR or PCR). |
| 10 | Assess GFR, albuminuria and total protein at least annually in people with CKD. Assess GFR, albuminuria and total protein more often for individuals at higher risk of progression, and/or where measurement will impact therapeutic decisions.* |
| 12 | In patients with risk factors for developing CKD, such as hypertension or diabetes mellitus or a history of cardiovascular disease, it is desirable to measure eGFR and albuminuria once a year. |
| 15 | Use the following table to guide the frequency of GFR monitoring for people with, or at risk of CKD:  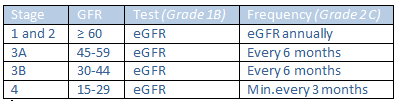   - Tailor the frequency depending on the presence of other risk factors on the development of terminal renal failure and the progression of the eGFR - Let the frequency of detecting proteinuria and complications suspend upon the eGFR, the progression of the eGFR and drug therapy |
| 16 | Metabolic complications of kidney failure: Measure serum potassium (on a fresh blood sample), calcium, phosphate, PTH and bicarbonate levels, and Hb in patiënts with CKD with a moderate (code orange) to strongly (code red) increased risk. In case of increased PTH, also measure vitamin D, and in the case of reduced Hb also measure ferritin and transferrin saturation. The frequency of these measurements depends on the degree of kidney damage.* |
| 17 | We recommend that all adults with CKD are offered annual vaccination with influenza vaccine, unless contraindicated. |
| 29 | Provide the following support: information for patients with CKD about their condition, a program for 'shared decision making', support for self-management (eg blood pressure, smoking cessation, exercise, diet and medication) and support in making a well informed choice. |
| 30 | Patients with CKD should be encouraged to:   - Undertake physical activity compatible with cardiovascular health and tolerance (aiming for at least 30 minutes 5 times per week) - Stop smoking - Obtain or maintain a healthy weight   - BMI 20 to 25   - Waist circumference ≥94 cm in men or ≥80 cm in women* - Limit alcohol |
| 32 | Individuals with CKD at high risk should receive expert dietary advice and information in the context of an education program, tailored to severity of CKD and the need to intervene on salt, phosphate, potassium, and protein intake where indicated. |
| 36 | In the context of drug management and patient safety, we recommend the use of metformin in patients with an eGFR >45ml/min./1.73m²; Metformin use has to be evaluated if eGFR is between 30-44ml/min./1.73m². Metformin has to be avoided with an eGFR <30 ml/min./1.73m². |
| 44 | We recommend that in both diabetic and non-diabetic adults with CKD and urine albumin excretion <30 mg/ 24 hours (or <30mg/g) whose office BP is consistently >140 mm Hg systolic or >90 mm Hg diastolic be treated with BP-lowering drugs to maintain a BP that is consistently ≤140 mm Hg systolic and ≤90 mm Hg diastolic. |
| 63 | In people with CKD, measure serum potassium concentrations and estimate the GFR before starting renin–angiotensin system antagonists and spironolactone.* Repeat these measurements between 1 and 2 weeks after starting renin–angiotensin system antagonists and after each dose increase. |
| 65 | If there is a decrease in eGFR or increase in serum creatinine after starting or increasing the dose of renin–angiotensin system antagonists, but <25% (eGFR) or <30% (serum creatinine) of baseline, repeat the test in 1–2 weeks. Do not modify the renin–angiotensin system antagonist dose if the change in eGFR is <25% or the change in serum creatinine is <30%. |
| 66 | We recommend temporary discontinuation of potentially nephrotoxic and renally excreted drugs in people with a GFR <60 ml/min/1.73 m2 (GFR categories G3a-G5) who have serious intercurrent illness (e.g. vomiting, diarrhea, limited fluid intake) and/or interfering condition (e.g. pneumonia)) that increases the risk of AKI.* These agents include, but are not limited to: RAAS blockers (including ACE-Is, ARBs, aldosterone inhibitors, direct renin inhibitors), diuretics, NSAIDs, metformin, lithium, and digoxin. |
| 68 | Determine the eGFR before each examination with contrast agent, if no recent (last 12 months) value is known. |
| 71 | Do not use oral phosphate-containing bowel preparations in people with a GFR < 60 ml/min/1.73 m2 (GFR categories G3a-G5) or in those known to be at risk of phosphate nephropathy. |
| 71.1 | Acute kidney injury or abrupt sustained fall in GFR. |
| 71.2 | GFR <30 ml/min/1,73 m2 (GFR categorie G4 or G5). |
| 71.16 | CKD combined with hypertension, which insufficiëntly responds to medical treatment with 4 or more antihypertensive drugs. |
| 71.23 | A CKD patient who is pregnant or when pregnancy is planned. |
| 72 | Refer high risk patiënts for inclusion in the care program. These are patients with:   - a chronic eGFR <30 ml/min./1,73 m² (eGFR categories G4-G5);   an eGFR between 30-45 ml/min./1,73 m² and ACR >200 mg/g for males or 300 mg/g for females, and/or proteïnuria >1000 mg/24h or a protein-creatininratio (PCR) >1 000 mg/g. |
| 74 | We suggest that people with progressive CKD should be managed in a multidisciplinary care setting. (2B) The multidisciplinary team should include or have access to dietary counseling, education and counseling about different RRT modalities, transplant options, vascular access surgery, and ethical, psychological, and social care. |

| **4.2. Recommendations switched from ‘discussion’ to ‘selection’ category** | |
| --- | --- |
| Nr | Description |
| 7 | Screening for proteinuria (ACR and/or protein/creatinine ratio) should be performed for all patients who are at high risk of kidney disease (patients with diabetes, hypertension, vascular disease, autoimmune disease, eGFR < 60 mL/min/1.73m2 or edema).* |
| 14 | Identify the rate of progression of CKD in people with a new finding of reduced GFR, repeat the GFR within 2 weeks to exclude causes of acute deterioration of GFR – for example, acute kidney injury or starting [renin–angiotensin system antagonist](http://www.nice.org.uk/guidance/cg182/chapter/recommendations#terms-used-in-this-guideline) therapy. |
| 18 | All CNI patients should have pneumococcal vaccination, unless contraindicated.* |
| 19 | All CNI patients should have hepatitis B vaccination unless contraindicated.* |
| 20 | In patients with CKD, an ARB or an ACE inhibitor should be used in case of a strongly increased albuminuria (> 300 mg / g or> 300 mg / 24 hours) (grade 1B) and preferably also in case of moderately increased albuminuria (30-300 mg / g or 30-300 mg / 24 hours). |
| 25 | The general practitioner should check for increased serum potassium in laboratory or hospital setting when real hyperpotassemia is probable.* |
| 33 | Complex diets for some patients with severe CKD require specialized guidance by a dietician to prevent dietary errors and/or malnutrition. Dietary advice about potassium and phosphate intake tailored to CKD stage is made by an appropriately qualified dietitian (Grade 1B). |
| 37 | In adults with CKD it is recommended to determine a one time lipid profile (total cholesterol, LDL cholesterol, HDL cholesterol, triglycerides). |
| 38 | Offer atorvastatin 20 mg for the primary or secondary prevention of CVD to people with CKD.   - Increase the dose if a greater than 40% reduction in non‑HDL cholesterol is not achieved (see recommendation 1.3.28) and eGFR is 30 ml/min/1.73 m^2^ or more. - Agree the use of higher doses with a renal specialist if eGFR is less than 30 ml/min/1.73 m^2^ |
| 43 | Target BP should be <130/80 (SBP range 120 - 129) mmHg*   - In patients with proteinuria ≥1 g/day. - In patients with normal urinary albumin concentrations. - In patients with diabetes. - In patients with a strongly increased albuminuria, first choice antihypertensive drugs to achieve these goals are ACE inhibitors or ARB's - In people with ACR  ≥70 mg/mmol |
| 48 | In people with CKD and heart failure, any escalation in therapy and/or clinical deterioration should prompt monitoring of eGFR and serum potassium concentration within 2 weeks.* |
| 64 | If there is a sustained rise in creatinine levels above 30% (or estimated glomerular filtration rate reduces >25%) from the baseline or serum potassium is >5.6 mmol/l during the first two months after commencement of ACEi/ARB therapy, reduce or discontinue the ACEi/ARB after excluding other precipitating factors and refer to a nephrologist/physician. |
| 69 | Inform the performer of any examination with a contrast agent of the patients’ renal function and mention CKD as an active element of the medical history. Also discuss the preventive measures that have to be taken.* |
| 71.5 | A consistent finding of significant albuminuria (ACR ≥300 mg/g or AER ≥ 300 mg/ 24 hours, approximately equivalent to PCR ≥ 500 mg/g or PER ≥500 mg/24 hours). |
| 71.7 | Haematuria with proteinuria (urine protein ≥0.5 g/day or uPCR ≥500 mg/g). |
| 71.9 | ACR ≥ 30 mg/mmol or more (ACR category A3), together with haematuria. |
| 71.10 | Progression of CKD: confirmed decline in GFR category accompanied by a 25% or greater drop in eGFR from baseline or a sustained decline in eGFR of more than 5 ml/min/1.73 m2 /year. |
| 71.15 | Patients with persisting microscopic hematuria of unknown origin.* |
| 71.17 | Persisting serum potassium abnormalities. |
| 71.18 | Recurrent or extensive nefrolithiasis. |
| 71.19 | Hereditary kidney disease. |
| 71.20 | When a.renalis stenosis is suspected or established. |
| 71.24 | Unclear cause of CKD. |

| **4.3. Recommendations switched from ‘no selection’ to ‘selection’ category** | |
| --- | --- |
| Nr | Description |
| 4 | Use the person's GFR and ACR categories to indicate their risk of adverse outcomes (for example, CKD progression, acute kidney injury, all-cause mortality and cardiovascular events) and discuss this with them |
| 11 | During treatment, aim to keep albumin concentrations <30 mg / mmol (or <300 mg / 24 hours, or proteinuria concentrations ​​<0.5 g / 24 hours), independent of blood pressure. This can be achieved by increasing the dose renin–angiotensin system antagonists  or Angiotensin receptor Blockers, or combining these with dietary salt restriction of 3 to 5 gram and / or a (thiazide) diuretic. In this case, determine feasibility individually. |
| 27 | In patients with CKD who have a reduced serum bicarbonate, treatment should be initiated using an oral bicarbonate to keep the serum bicarbonate level within the normal range.* |
| 54 | A trial of oral or IV iron is suggested to patients with anemia without iron supplementation and with/without ESA if an increase in Hb concentration is preferred without initiating or increasing ESA dosage, transferrinesaturation ≤25% and ferritine concentration<200 mg/l is. |
| 57 | In case of Vit D deficiency in a patient with CKD, Vit D should be substituted.* |
| 58 | Do not prescribe bisphosphonate treatment in people with GFR 30 ml/min/1.73 m2 (GFR categories G4-G5) without a strong clinical rationale. |

| **4.4. Recommendations switched from ‘selection’ to ‘no selection’ category** | |
| --- | --- |
| Nr | Description |
| 28 | Offer tailored education and support programs in the self-management of CKD patients. Referral to a specialized nurse is recommended in order to ameliorate understanding of their condition, to ameliorate compliance to lifestyle changes and drug treatment. These measures are taken to stabilize parameters and to preserve renal function as long as possible. |
| 62 | In patients with heart failure and CKD, avoid the use of digoxin due to the higher risk of intoxication. When digoxin use is required, reduced doses will be administered. |

| **4.5. Recommendations switched from ‘discussion’ to ‘no selection’ category** | |
| --- | --- |
| Nr | Description |
| 21 | Treat all patients with a corrected proteinuria >  900 mg / g (100 mg / mmol) with an ACE-I regardless of blood pressure. |
| 22 | An ACE inhibitor (ACE-I) is the preferred antihypertensive agent in all diabetic patients with CKD and in all patients with a corrected proteinuria > 270 mg/g (30 mg/mmol). |
| 34 | We recommend a target hemoglobin A1c (HbA1c) of ~7.0% (53 mmol/mol) to prevent or delay progression of the microvascular complications of diabetes, including diabetic kidney disease. |
| 35 | An HbA1c level of <53 mmol / mol (<7.0%) should not be strived for in the following circumstances:   - patients at risk of hypoglycemia - people with co-morbidity or limited life expectancy and risk of hypoglycemia |
| 39 | In adults aged ≥50 years with an eGFR <60 ml/min/1.73 m2 treatment with statins or a combination statin/ezetimibe is advised. |
| 40 | In adults aged ≥50 years with CKD and an eGFR <60 ml/min/1.73 m2  treatment with statins is advised. |
| 49 | Offer antiplatelet drugs (acetylsalicylic acid) to people with CKD for the secondary prevention of cardiovascular disease, but be aware of the increased risk of bleeding. |
| 50 | Combination of clopidogrel with aspirin should be avoided in patients with CKD (unless compelling indications are present). |
| 51 | Consider apixaban in preference to warfarin in people with a confirmed eGFR of 30–50 ml/min/1.73 m^2^ and non-valvular atrial fibrillation who have 1 or more of the following risk factors:   - prior stroke or transient ischaemic attack - age > 75 year or older - hypertension - diabetes mellitus - symptomatic heart failure |
| 52 | If not already measured, check the haemoglobin level in people with a GFR < 45 ml/min/1.73 m^2^ (GFR category G3b, G4 or G5) to identify anaemia (haemoglobin less than 110 g/L [11.0 g/dl]). Determine the subsequent frequency of testing by the measured value and the clinical circumstances. |
| 55 | Measure serum calcium, phosphate and PTH concentrations in people with a GFR of less than 30 ml/min/1.73 m2 (GFR category G4 or G5). Determine the subsequent frequency of testing by the measured values and the clinical circumstances. Where doubt exists, seek specialist opinion. |
| 59 | Offer bisphosphonates if indicated for the prevention and treatment of osteoporosis in people with a GFR of 30 ml/min/1.73 m^2^ or more (GFR category G1, G2 or G3). |
| 60 | In patients with heart failure, prescribe diuretics at the lowest effective dose, slowly alter doses and only use diuretics in case of symptoms of fluid retention. |
| 61 | In patiënts with heart failure and CKD, avoid the use of spironolactone. |
| 71.11 | Patients with progressive CKD, being an eGFR between 30 and 45 ml/min./1,73 m² and a decline of eGFR >10 ml/min in five years time or > 5 ml/min in two years time. |
| 71.12 | Rapidly declining renal function (loss of glomerular filtration rate/GFR >5 ml/min/1.73m2 in one year or >10 ml/min/1.73m2 Within five years) |
| 71.13 | Sustained decrease in GFR of 25% or more, and a change in GFR category or sustained decrease in GFR of 15 ml/min/1.73 m^2^ or more within 12 months |
| 71.14 | Dysmorphic  erytrocytes in urine sedimentation (>20 pgv). |
| 71.22 | Suspected glomerular disease. |
| 73 | We recommend timely referral for planning renal replacement therapy (RRT) in people with progressive CKD in whom the risk of kidney failure within 1 year is 10–20% or higher , as determined by validated risk prediction tools. |
